# Assessing the risk of performance and detection bias in Cochrane reviews as a joint domain is less accurate compared to two separate domains

**DOI:** 10.1186/s12874-021-01339-1

**Published:** 2021-07-18

**Authors:** Ognjen Barcot, Matija Boric, Svjetlana Dosenovic, Livia Puljak

**Affiliations:** 1 Department of Surgery, University Hospital Split, Split, Croatia; 2 Department of Anesthesiology and Intensive Care, University Hospital Split, Split, Croatia; 3 Center for Evidence-Based Medicine and Health Care, Catholic University of Croatia, Ilica 242, 10000 Zagreb, Croatia

**Keywords:** Risk of bias, Cochrane, Systematic reviews, Performance bias, Detection bias

## Abstract

**Background:**

Initially, the Cochrane risk of bias (RoB) tool had a domain for “blinding of participants, personnel and outcome assessors”. In the 2011 tool, the assessment of blinding was split into two domains: blinding of participants and personnel (performance bias) and blinding of outcome assessors (detection bias). The aims of this study were twofold; first, to analyze the frequency of usage of the joint blinding domain (a single domain for performance and detection bias), and second, to assess the proportion of adequate assessments made in the joint versus single RoB domains for blinding by comparing whether authors’ RoB judgments were supported by explanatory comments in line with the Cochrane Handbook recommendations.

**Methods:**

We extracted information about the assessment of blinding from RoB tables (judgment, comment, and whether it was specified which outcome type; e.g., objective, subjective) of 729 Cochrane reviews published in 2015-2016. In the Cochrane RoB tool, judgment (low, unclear or high risk) needs to be accompanied by a transparent comment, in which authors provide a summary justifying RoB judgment, to ensure transparency in how these judgments were reached. We reassessed RoB based on the supporting comments reported in Cochrane RoB tables, in line with instructions from the Cochrane Handbook. Then, we compared our new assessments to judgments made by Cochrane authors. We compared the frequency of adequate judgments in reviews with two separate domains for blinding versus those with a joint domain for blinding.

**Results:**

The total number of assessments for performance bias was 6918, with 8656 for detection bias and 3169 for the joint domain. The frequency of adequate assessments was 74% for performance bias, 78% for detection bias, and 59% for the joint domain. The lowest frequency of adequate assessments was found when Cochrane authors judged low risk – 47% in performance bias, 62% in detection bias, and 31% in the joint domain. The joint domain and detection bias domain had a similar proportion of specified outcome types (17% and 18%, respectively).

**Conclusions:**

Splitting joint RoB assessment about blinding into two domains was justified because the frequency of adequate judgments was higher in separate domains. Specification of outcome types in RoB domains should be further scrutinized.

**Supplementary Information:**

The online version contains supplementary material available at 10.1186/s12874-021-01339-1.

## Background

Risk of bias (RoB) assessment is a crucial methodological aspect of systematic reviews and an obligatory part of Cochrane reviews [1]. A 2008 Cochrane RoB tool [2] had six domains, and one of them assessed “blinding of participants, personnel and outcome assessors” [3]. In the 2011 Cochrane RoB tool [4], this joint domain was split into two domains, one for blinding of participants and personnel (performance bias) and one for blinding of outcome assessors (detection bias) [1]. A new version of the Cochrane Handbook was published in 2019 [5], including the RoB 2 tool – where the assessment of blinding of the three key groups of individuals is split into three separate assessments [6]. With the evolution of the research methods, it is important to compare the revised versions with the previous versions, to ensure that the revised versions are indeed a step forward.

We have shown previously that Cochrane reviews authors frequently made inadequate RoB judgments using the 2011 RoB tool [7–10]. More specifically, in the performance bias domain, the overall proportion of RoB judgments following the recommendations from Cochrane Handbook (adequate judgments) was 73.6%, and the main error in reported RoB judgments was the presumption of healthcare providers being adequately blinded [11]. In the detection bias domain, the frequency of adequate judgments was 77.9%, and the main error was the improper categorization of outcomes (subjective vs. objective) [12]. Furthermore, we noticed that Cochrane authors still frequently use the joint domain for blinding of key individuals by making modifications to the 2011 Cochrane RoB tool, even though the tool contains two distinct blinding domains.

The aims of this study were twofold: first, to analyze the frequency of usage of the joint blinding domain, and second, to assess the proportion of adequate assessments made in the joint versus single RoB domains for blinding in Cochrane reviews by comparing whether authors’ RoB judgments were supported by explanatory comments in line with the Cochrane Handbook recommendations.

## Methods

### Study design

This was a primary methodological study in which we analyzed the methodology of Cochrane reviews published in the Cochrane Database of Systematic Reviews (CDSR). The study protocol was prepared a priori, but the protocol was not published. Raw data generated in this study are available on the Open Science Framework project page on the link https://osf.io/fmjxz/.

### Inclusion and exclusion criteria

CDSR was searched for all reviews of randomized controlled trials (RCTs) of interventions (or both RCTs and non-randomized studies, but we analyzed RoB assessments only for RCTs) published from July 2015 to June 2016. This was a large, one-year convenient sample based on our previous studies [8, 11, 12], four years after the introduction of the 2011 RoB tool when it is expected from the review authors to have adopted the new methodology (tool). An advanced search option was used to limit results to content type and publication date. We excluded diagnostic Cochrane reviews, overviews of systematic reviews, empty or withdrawn reviews, and other Cochrane reviews containing no RCTs about interventions.

### Screening for study eligibility

Titles and abstracts of Cochrane reviews were screened for eligibility by the first author (OB) and verified by another author (SD). The second author was verifying that no reviews were erroneously included/excluded. A list of analyzed Cochrane systematic reviews and studies included is presented in Supplementary file 1. The final unit of assessment was the risk of bias judgments for performance and detection bias of all the trials included in the eligible reviews.

### Data extraction

The first author (OB) wrote series of macro-instructions in Visual Basic for Applications (VBA, Microsoft, Redmond, WA, USA) to automate data scraping of all the CSRs included in the study from The Cochrane Library webpage to Microsoft Excel 2010 (Microsoft, Redmond, WA, USA) workbook. The automatic extraction of RoB tables for every eligible Cochrane review was then done with a new set of coded instructions, as in our previous studies (https://osf.io/fmjxz/) [8]. Errors during data extraction were logged and checked manually by the lead author.

During error checkup and manual search for missing data, in two separate analyses of the domain for blinding of participants and personnel [11] and for blinding of outcome assessment [12], it was noticed that there is a subgroup of Cochrane reviews which used a joint domain for blinding of participants, personnel and outcome assessors. This particular subgroup of Cochrane reviews has been marked, selected, extracted, and used for this study, and it was not a part of any past analysis. The results of the previous analyses of the domain for blinding of participants and personnel and of the blinding of outcome assessment served in this work as comparators [11, 12]. The dataset used in this work was not a part of the previous analyses.

In our previous study [8], the first author (OB) developed a specific user interface (MS Excel VBA User Form) to facilitate parsing. This interface, for filling the MS Excel table, simply helped the authors with the transformation of natural language text (comments, citations) to ordinal or nominal variables for further analysis. The interface did not, in any way, change, calculate, or suggest the decision of the authors, i.e. the decisions were made by the authors and not automated.

Pilot tests (adjustments of the tool) were done in the studies mentioned above by most experienced authors (OB, SD and MB) on samples of 500 RCTs each. These authors used the same tool in this study, with no changes in appearance or coding.

### Assessment of adequacy for joint blinding domain

The Cochrane Handbook explicitly instructs authors: ‘*The support for judgement provides a succinct summary from which judgements of risk of bias can be made and aims to ensure transparency in how these judgements are reached*.’. These supporting comments should be sufficiently informative for making a judgment. Thus, we assessed whether Cochrane authors’ RoB judgments were supported by the comments provided by authors in RoB tables.

In the first step of assessing judgments’ adequacy we made a new assessment of RoB based on supporting comments from Cochrane reviews, based on instructions from the Cochrane Handbook. In the second step, we compared these de novo judgments with judgments published in Cochrane reviews.

The new assessment of RoB for the joint domain was made for RCTs in which Cochrane authors provided both a judgment (risk of bias is low, high, or unclear) and accompanying comment. The only source for these assessments was the accompanying comment from the RoB table and the description of the intervention provided by the Cochrane authors, not the full texts of the original studies. The mentioned user interface was used just to enhance the visualization of the mentioned data and to ease the fulfilling of the MS Excel table. No full texts of the primary studies were analyzed. We followed instructions for rating detection bias from the Cochrane Handbook (Sects. 8.11.2 and 8.12.2) [13] and defined that four main questions need to be correctly answered to assess the blinding bias. Question #1: who was blinded? – to identify subjects (participants, personnel, and outcome assessors). Question #2: was blinding achieved and complete for relevant subjects? – because subjects have overlapping roles (e.g., participants can be self-assessors). Question #3: what was the outcome category? – to identify outcomes susceptible to bias. Question #4: can this outcome be influenced by lack of blinding – because not all outcomes are equally prone to performance and detection bias. All of the authors were experienced in RoB assessments as well as being clinicians (OB—senior surgeon, MB – experienced surgeon, and SD – anesthesiologist) considering the expertise in clinical aspects of outcome categorization.

Two authors (MB, SD) reassessed the RoB for their respective half of the sample. Due to the redundancy of the questions (Q#1 vs Q#3 and Q#2 vs Q#4) the lead author checked for the discrepancies and eventually corrected the assessment in about 20% of the cases. Lastly, we compared our new RoB assessments with the assessments made by the Cochrane authors. The proportion of RoB assessments by Cochrane authors matching the reassessment adhering to the Cochrane Handbook was termed – adequacy. The opposite term, inadequacy, does not necessarily mean the original judgment is incorrect but simply not justified by the supporting comment [14, 15].

### Primary outcomes

RoB judgments, for the joint blinding domain, assigned by Cochrane authors were analyzed by number and adequacy (the proportion of judgments adhering to the Cochrane Handbook in all reassessed judgments). The definition standard in our assessment was the Cochrane Handbook, as specified in Table 8.4.d [16]. We considered that Cochrane authors' judgment was inadequate if it did not completely adhere to the Cochrane Handbook guidance (based on answers if blinding was achieved and whether the outcome was susceptible to bias). We compared adequacy in this joint domain to adequacy for the two individual domains – i.e., blinding of participants and personnel domain, and blinding of outcome assessor domain, based on results from our past works [11, 12].

### Secondary outcomes

We analyzed the distribution of different types of outcomes (i.e., proportions of types of outcomes in all assessed judgments) in the performance bias domain, detection bias domain, and the joint domain. Primarily, our user interface offered a variety of pre-specified outcomes: all outcomes, not specified, objective (e.g., lab results, mortality, overall survival), outcomes rated/related/reported (RRR) by the clinician (e.g., complications such as occurrence if wound infection, adverse events such as pulmonary embolism, assessor/clinician related such as eye background description), patient-rated/related/reported or patient RRR (e.g., private phenomena such as the presence of fear, behavioral) and subjective in general. Due to overlap of characteristics of some types of outcomes and relatively small numbers, we used and analyzed a reduced list of outcomes: (i) all outcomes or not specified, (ii) objective outcomes (or subject independent), and (iii) subjective outcomes (including both clinician RRR and patient RRR). “Not specified” outcomes were the ones with a cell left blank in the RoB table (by default meaning all outcomes when inquired through RevMan interface) and thus were grouped. We also compared the distribution of severity of reassessed judgments (low, unclear, or high) for all three domains (performance bias domain, detection bias domain, and joint blinding domain).

Apart from the analysis of the whole joint blinding domain and comparison between two separate standard blinding domains, we performed analyses of subsamples when the joint domain for performance and detection bias was split into multiple subdomains according to the various outcomes. Here, we compared distribution (judgments of high, unclear, or low risk of bias) and adequacy of judgments to the whole sample.

### Statistics

We presented descriptive data as frequencies and percentages. We used type I error α = 0.05 and type II error β = 0.2 for all statistical tests. Statistical analyses were performed using MedCalc for Windows, version 12.5.0.0 (MedCalc Software, Ostend, Belgium). Kolmogorov–Smirnov test was used to assess normality for all the datasets. For comparison of independent samples of non-parametric data, the Mann–Whitney test was used, and the Wilcoxon test was used for paired samples. A chi-squared test was utilized to asset the difference in proportions. Tukey fences were used for suspected outliers. Hypotheses, outcome measures, statistical tests used, and results are logged in Supplementary file 2.

## Results

**Fig. 1:**
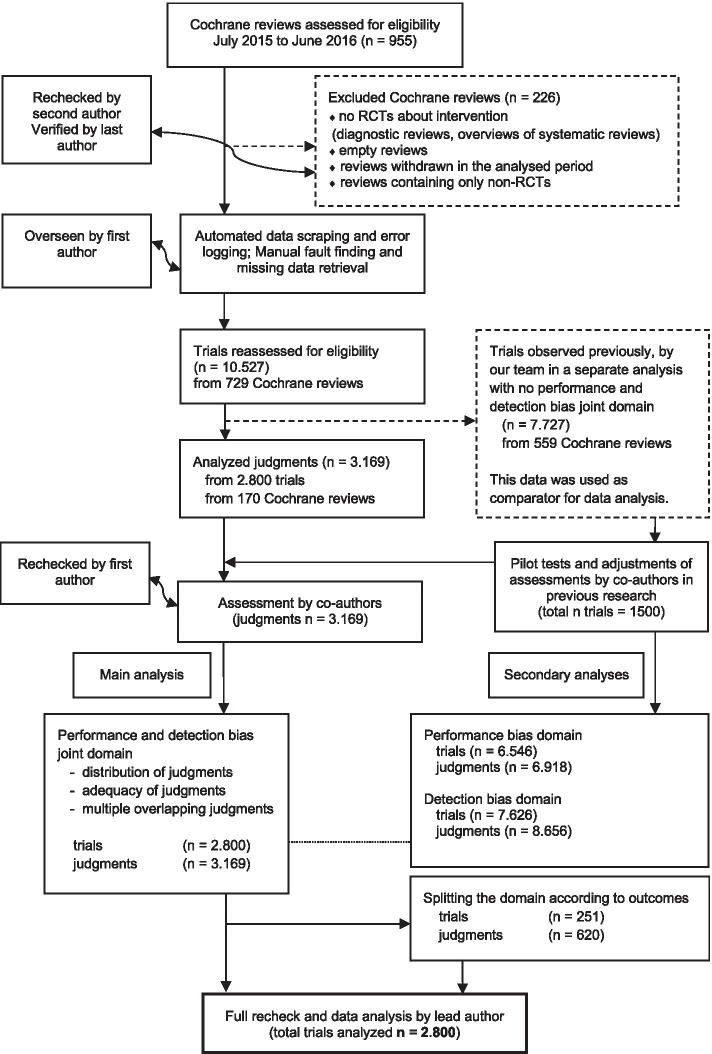
Flow diagram of the progress through the phases of the study and our previous studies

The analysis was conducted on 729 Cochrane reviews, with 10,527 included trials. There were a total of 6918 assessments for performance bias, 8656 for detection bias, and 3169 for the joint domain (Fig. 1, Table 1). Only 28 studies appeared in multiple reviews for the joint domain with a total of 57 judgments.

**Table 1:** Risk of bias (RoB) judgment adequacy in Cochrane reviews using two separate blinding domains compared to the joint domain

	**Reassessment of judgments our studies**														
**Outcome category**	**Joint blinding domain**					**Blinding of participants and personnel**					**Blinding of outcome assessors**				
**Judgment by Cochrane authors**	**High risk**	**Low risk**	**Unclear risk**	**Total N (%)**	**Adeq (%)**	**High risk**	**Low risk**	**Unclear risk**	**Total N (%)**	**Adeq (%)**	**High risk**	**Low risk**	**Unclear risk**	**Total N (%)**	**Adeq (%)**
**All outcomes or not specified**	**721**	**332**	**1581**	**2634 (83.1%)**	**58.1%**	**2642**	**1030**	**2909**	**6581 (95.1%)**	**73.0%**	**1355**	**1502**	**4253**	**7110 (82.1%)**	**76.9%**
High risk	592	11	147	750	78.9%	2206	5	184	2395	92.1%	1129	7	215	1351	83.6%
Low risk	39	273	769	1081	25.3%	94	1000	1129	2223	45.0%	43	1448	1149	2640	54.8%
Unclear risk	90	48	665	803	82.8%	342	25	1596	1963	81.3%	183	47	2889	3119	92.6%
**Objective outcomes**	**0**	**84**	**0**	**97 (3.0%)**	**81.4%**	**53**	**69**	**6**	**128 (1.9%)**	**85.9%**	**0**	**408**	**0**	**408 (4.7%)**	**71.6%**
High risk	0	0	0	0	N/A	41	0	0	41	100%	0	9	0	9	0%
Low risk	0	79	0	79	100%	1	69	6	76	90.8%	0	292	0	292	100%
Unclear risk	0	18	0	18	0%	11	0	0	11	0%	0	107	0	107	0%
**Subjective outcomes**	**213**	**52**	**173**	**438 (13.8%)**	**57.3%**	**162**	**25**	**22**	**209 (3.0%)**	**84.7%**	**357**	**356**	**425**	**1138 (13.1%)**	**86.9%**
High risk	134	2	37	173	77.5%	140	0	0	151	92.7%	311	6	2	319	97.5%
Low risk	11	41	60	112	36.6%	0	24	9	33	72.7%	3	347	92	442	78.5%
Unclear risk	68	9	76	153	49.7%	22	1	13	36	36.1%	43	3	331	377	87.8%
**Overall (%)**	**934 (28.9%)**	**481 (14.9%)**	**1754 (54.3%)**	**3169 (100%)**	**58.7%**	**2857 (41.3%)**	**1124 (16.2%)**	**2937 (42.5%)**	**6918 (100%)**	**73.6%**	**1712 (19.8%)**	**2266 (26.2%)**	**4678 (54.0%)**	**8656 (100%)**	**77.9%**
High risk	726	13	184	923	78.7%	2387	5	184	2575	92.7%	1440	22	217	1679	85.8%
Low risk	50	393	829	1272	30.9%	95	1093	1144	2325	47.0%	46	2087	1241	3374	61.9%
Unclear risk	158	75	741	974	76.1%	375	26	1609	2005	80.2%	226	157	3220	3603	89.4%

*Adeq* Adequacy, *RoB* risk of bias

### Primary outcome

The overall frequency of adequate assessments (the Cochrane authors' assessment matching to that of the assessors in the present evaluation, thus adhering to the Cochrane Handbook) was the lowest (59%; 1860/3169) in the joint domain (Table 1). This was significantly lower compared to 74% (5089/6918) for the performance bias domain (*p* < 0.0001) and versus 78% (6747/8656) for detection bias domain (*p* < 0.0001, Table 1, Supplementary file 2).

### Secondary outcomes

Similar distribution of types of outcomes (subjective / objective / all) that authors specified was found for detection bias domain (13% / 5% / 82%) and joint blinding domain (14% / 3% / 83%, *p* = 0.358; Table 1). The distribution of reassessed judgments (high / low / unclear) differed through all three domains: joint blinding domain (29% / 15% / 54%) vs performance bias domain (41% / 16% / 43%) vs detection bias domain (20% / 26% / 54%); (*p* < 0.05; Table 1, overall row). In all of the three domains, the lowest frequency of adequate assessments was found when Cochrane authors made the judgment of low risk – 47% in performance bias, 62% in detection bias and 31% in the joint domain (Table 1, adequacy column).

Similar to our analyses in previous works, this analysis yielded ‘worse’ RoB judgment in 1046 (32.4%) of those trials (i.e., the judgment changed from originally low to unclear, or unclear to high), and ‘better’ RoB judgments in 273 (8.5%) trials (i.e., the judgment changed from originally unclear to low, or high to unclear), as shown in Table 2. We found that 198 (21.2%) of high-risk judgments made by Cochrane authors were reassessed as unclear or low, while 238 (23.6%) of the assigned unclear risk judgments were reassessed as either high or low risk. Two-thirds of the judgments 883 (68.8%) assigned low RoB for the joint domain were calculated to be of unclear or high risk.

**Table 2:** Difference in judgment provided by the Cochrane review authors and judgment in line with the Cochrane Handbook across different domains

**Direction of change of RoB judgment**	**Joint domain N (%)**	**Performance bias domain**^**a**^ **N (%)**	**Detection bias domain**^b^ **N (%)**
**To higher RoB**	**1046** **(32.4%)**	**1614** **(23.3%)**	**1513** **(17.5%)**
Low to High	50 (3.9%^c^)	95 (4.1%^c^)	46 (1.4%^c^)
Unclear to High	163 (16.2%^c^)	375 (18.6%^c^)	226 (6.3%^c^)
Low to Unclear	833 (64.9%^c^)	1144 (49.0%^c^)	1241 (36.8%^c^)
**To lower RoB**	**273** **(8.5%)**	**214** **(3.1%)**	**396** **(4.6%)**
High to Unclear	185 (19.8%^c^)	183 (7.1%^c^)	217 (12.9%^c^)
Unclear to Low	75 (7.4%^c^)	26 (1.3%^c^)	157 (4.4%^c^)
High to Low	13 (1.4%^c^)	5 (0.2%^c^)	22 (1.3%^c^)
**Unchanged**	**1909** **(59.1%)**	**5090** **(73.6%)**	**6747** **(77.9%)**
High	738 (78.8%^c^)	2386 (92.7%^c^)	1440 (85.8%^c^)
Unclear	770 (76.4%^c^)	1610 (80.1%^c^)	3220 (89.4%^c^)
Low	401 (31.2%^c^)	1094 (46.9%^c^)	2087 (61.9%^c^)

^a^ domain for blinding of participants and personnel, data from Barcot et al. 2019 [11]; percentage of assigned judgment subgroup; ^b^ domain for blinding of the outcome assessors, data from Barcot et al. 2020 [12]; ^c^ percentage of assigned judgment subgroup

Acronyms: RoB = risk of bias

### Distribution and adequacy of judgments in the joint domain for subjective outcomes

Assessment of subjective outcomes demonstrated significantly lower adequacy in the joint blinding domain (57.3%) than in the two separate domains (performance bias domain 84.7%, *p* < 0.05; detection bias domain 86.9%, *p* < 0.05); see Table 1 and 3. In-depth analysis of assessments demonstrated the highest number of inadequate judgments among the subgroup of clinician RRR outcomes making 56% (*N* = 111) out of 187 inadequate judgments in the subjective outcomes group (Table 3). Furthermore, inadequate assessments were most common with judgments of low risk of bias (56/187, 30%) (Table 3 – inadequate judgments column).

**Table 3:** Distribution and adequacy of judgments in the joint blinding domain for subjective outcomes classified as subjective

Outcome category	Reassessments of judgments in our study			Total		Inadequate	
Judgment by Cochrane authors	High risk	Low risk	Unclear risk	**N (%)**		**judgments N (% total)**	
**Clinician RRR**^**a**^	92	41	120	253	(57.8%)	111	(59.4%)
High risk	60	2	20	82	(32.4%)	22	(11.8%)
Low risk	8	30	48	86	(34.0%)	56	(29.9%)
Unclear risk	24	9	52	85	(33.6%)	33	(17.6%)
**Patient RRR**^**a**^	63	9	36	108	(24.7%)	46	(24.6%)
High risk	36	0	17	53	(49.1%)	17	(9.1%)
Low risk	2	9	2	13	(12.0%)	4	(2.1%)
Unclear risk	25	0	17	42	(38.9%)	25	(13.4%)
**Subjective**	58	2	17	77	(17.6%)	30	(16.0%)
High risk	38	0	0	38	(49.4%)	0	(0.0%)
Low risk	1	2	10	13	(16.9%)	11	(5.9%)
Unclear risk	19	0	7	26	(33.8%)	19	(10.2%)
**Total**	213 (48.6%)	52 (11.9%)	173 (39.5%)	438	(100%)	187	(42.7%)

^a^ RRR =—rated /—related /—reported

### Distribution and adequacy of judgments when the joint blinding domain is split according to various outcomes

Distribution of categories of outcomes in the whole joint blinding domain (3169 judgments: 83% all outcomes, 3% objective, 14% subjective) and its subsample of trials with domain split according to the type of outcome (*N* = 251 trials, *N* = 620 judgments, all outcomes 40%, objective 12%, subjective 48%) was significantly different (*p* < 0.05; Table 4). In this subsample, the percentage of adequate judgments for all or not specified outcomes was 40% compared to 83% in the whole sample (*p* < 0.0001; Table 4). Out of these 251 trials, 168 (accounting for 416 judgments) had the risk of detection bias judgment identical within all of their split outcomes (meaning in a single trial, all of the RoB judgments were of the same level: all high, all low, or all unclear). This subsample (Table 4) showed lower adequacy of judgments (44% vs. 59%, *p* < 0.05) than the whole sample. On the other hand, judgments in the rest of the trials which judged the risk of detection bias differently were as (in)adequate as in the whole sample (58% vs 59%, *p* = 0.978).

**Table 4:** Distribution and adequacy of judgments when the joint domain is split according to various outcomes

	**Judgments for domains that were split for various outcomes**									**Whole sample**		
	**Overall**			**Different**			**All the same**					
**Types of outcomes**	**N**	**(%N)**	**Adeq**	**N**	**(%N)**	**Adeq**	**N**	**(%N)**	**Adeq**	**N**	**(%N)**	**Adeq**
**All outcomes**^**a**^	248	(40%)	40%	76	(37%)	74%	172	(41%)	26%	2634	83%	58%
**Objective**^**b**^	72	(12%)	75%	7	(3%)	57%	65	(16%)	77%	97	3%	81%
**Subjective**	300	(48%)	50%	121	(59%)	49%	179	(43%)	50%	438	14%	57%
**Total**	620		49%	204		58%	416		44%	3169		59%

Adeq = Adequacy; ^a^All or not specified outcomes; ^b^objectively measured / subject independent

## Discussion

The main finding of this study is that adequacy of RoB judgments about blinding in Cochrane reviews was better when Cochrane authors judged blinding of the key participants in two separate domains (i.e., one domain for participants and personnel, and another domain for outcome assessors), compared to one joint domain for all those three groups of individuals.

Separate domains force the review authors to provide a separate judgment for different groups of individuals; thus, assessments become more precise with split domains. This separation is relevant because there are specific difficulties for blinding different groups [8]. There might be a problem with the blinding of personnel, usually associated with the type of intervention. Also, participants may not be only passive recipients of interventions; they are often self-assessors of outcomes when patient-reported outcomes are used. Thus, the lack of blinding of specific individuals involved with a trial does not lead to a high risk of bias only if the outcome is objective.

Sometimes Cochrane reviewers specified the type of outcome for which the domain was judged, i.e., whether they considered an outcome objective or subjective. The distribution of assessments according to different types of outcomes demonstrated certain similarities between the joint domain and the detection bias domain. Both domains had a very low rate of specified outcomes (17% joint blinding, 18% detection bias domain), but in contrast to the performance bias domain (with less than 5%), this might be seen as a success. We might conclude that much more effort has to be introduced to identify outcomes susceptible to bias related to blinding in trials, as well as taking care of proper blinding of the subjects.

In our previous study [8], assessments of outcomes defined by Cochrane authors as subjective were significantly more often accurate than outcomes in general. This was due to the relatively high proportion of judgments for “high risk” that were highly accurate. Increased adequacy (objective 81% vs. subjective 57%) came from better precision in the definition of objective outcomes. However, less adequate assessments of subjective judgments did not originate from the distribution of risk judgments, which did not differ from the detection bias domain, as stated before.

Clinician-related outcomes that were judged with low risk by Cochrane authors contributed the most to inadequate judgments. Among these, the majority defined such an outcome as objective, even though it was not (e.g., completeness of treatment or established clinical test rating), a problem linked to the detection bias domain. Some RoB tables did not have enough detail in supporting comment, e.g., they used a vague “double-blind” comment without specifying who exactly was blinded, which is a frequent explanation that reviewers use when describing their rationale for assessing the performance bias domain. This likely stems from the primary studies, where the usage of the term “double-blind” without any further details about blinding of key individuals is widespread; however, it has been shown repeatedly that the term is ambiguous and that it means different things to different researchers [17–19]. Thus, it is recommended that trialists should not use the term “double-blind”, but instead report transparently who exactly was blinded in a trial.

Additionally, we found that Cochrane authors sometimes split the joint domain into multiple subdomains for different outcomes. While this approach may be considered more transparent regarding different types of outcomes (showing, for example, separate judgments for subjective vs objective outcomes), such reviews had much worse results in terms of RoB adequacy. Therefore, we have demonstrated that splitting a joint blinding domain only according to the outcomes is not a preferable solution. Splitting (i.e., providing more granular information) should be used based on the different groups of individuals (three separate domains for judging whether participants, personnel and outcome assessors were blinded) and the susceptibility of an outcome to be influenced by knowledge of intervention received, such as in RoB 2.

Cochrane methods are continuously evolving. Our findings indicate that the decision to split the domain about blinding into two separate domains was justified, as the adequacy of judgment was better in separate domains. This is easily understood, as the joint domain refers to multiple groups of participants, and therefore it may be unclear how Cochrane authors are judging RoB related to blinding in domains covering more than one group of participants. For this reason, we hypothesize that the decision to split further the assessment of RoB related to blinding to three assessments in the RoB tool 2 will prove to be even more advantageous for accurate assessments [6]. However, this hypothesis will need to be tested in the future, as the RoB tool 2 is still in its implementation phase, and Cochrane authors are still not obliged to use it.

### Strengths and limitations

Our study's strength is that we have analyzed a large number of Cochrane reviews with more than ten thousand trials included. We have focused on Cochrane reviews because the use of Cochrane methods, i.e., Cochrane RoB tool, is mandatory in Cochrane reviews, but our results are also relevant for non-Cochrane systematic reviews. Although the majority of non-Cochrane reviews do not report on RoB [20, 21], when they do, their reporting is sub-optimal [1, 22], and their authors also use Cochrane RoB tool inadequately [22].

There are also some potential limitations to our work. Firstly, even though we prepared a study protocol before commencing this study, we did not publish the study protocol, as there is still no requirement in the international community for publishing protocols of studies other than clinical trials. However, we are aware that publishing the study protocol prospectively could be important for readers for appraising the risk of selective reporting and any other biases that may have occurred due to changes to the protocol during the study.

Additionally, there may be differences between assessments made in the original Cochrane reviews in data availability, as the Cochrane authors have appraised reports of included RCTs, and they might have contacted trial authors for clarifications. For this reassessment, we relied on comments provided by Cochrane authors in RoB tables. Cochrane authors should provide informative comments to explain the rationale for their judgments as instructed by the Cochrane Handbook. If the authors did not report all the key information transparently in the supporting comment, the judgment might not be sufficiently justified. The concept of adequacy, used in this study, might still be subjective because it was ultimately determined by the authors of this manuscript (although we did our best to follow guidance from the Cochrane Handbook strictly) [14, 15].

The categorization of outcomes as objective or subjective was made by our team. It needs to be emphasized that outcomes are often not fully objective or fully subjective but instead fall somewhere on the continuum between objective and subjective. It is possible that clinician input during the execution of the Cochrane reviews could have influenced the risk of bias judgments, at least partially explaining why the assessments in the reviews would be different from those undertaken in this study.

Furthermore, some may consider that blinding is not well defined in the Cochrane Handbook and that neither Cochrane authors nor our team could categorically determine whether the Cochrane Handbook criteria have been met. For this reason, we have transparently reported our judgments and rationale behind our assessments: raw data generated in this and related manuscripts can be located on the Open Science Framework project page on the link https://osf.io/fmjxz/.

It could also be argued that blinding is a poorly defined construct. For example, blinding could be a property of the trial methods (in which case assessment of blinding would involve assessing the presence/adequacy of the placebo or sham), but also it can be manifested in the knowledge or beliefs of key individuals about the allocation of interventions; in the latter case evaluation of blinding would involve assessing knowledge or beliefs of the key individuals about the allocation [23].

In this study, we analyzed Cochrane reviews published within a limited date range from July 2015 to June 2016. However, we have no reason to believe that the results would be different if we have used a more extended period after June 2016. We did not choose an earlier period than July 2015 because the analyzed Cochrane RoB tool was published in 2011, and we considered it essential to leave out the first few years after its publication to allow Cochrane reviewers to adopt the new methodology. Regarding the inclusion of a higher number of more recently published Cochrane reviews, we have evidence from our recent methodological study that this is not needed [24]. In that study, we initially analyzed 768 Cochrane reviews that were published in 2015 and 2016. Based on editors' request, we expanded our eligibility criteria to two more years, up to the year 2018. However, our subsequent analysis indicated no difference in our results at all, despite doubling the number of included Cochrane reviews and expanding our eligibility period from one to three years [24]. Additionally, there are no uniform guidelines regarding search periods in methodological studies, and it has been suggested that extended periods should be considered when some significant changes can be expected [25]. Thus, we argue that our data are relevant, considering the eligibility criteria we used.

## Conclusion

Our results indicate that splitting the joint RoB domain about blinding key individuals into two separate domains was justified. Cochrane authors more frequently made adequate judgments in separate domains for blinding. We anticipate that this should result in an even higher adequacy of judgments in the Cochrane RoB 2 tool, but this will need to be confirmed after its full implementation in Cochrane reviews.

## Supplementary Information

**Additional file 1.** List of analyzed Cochrane systematic reviews and studies included.

**Additional file 2.** Table overview of the hypotheses, outcome measures, statistical testsused and results.

## Data Availability

Raw data generated in this study are available on the Open Science Framework project page on the link https://osf.io/fmjxz/.

## Abbreviations

CDSR: Cochrane Database of Systematic Reviews
RRR: Outcomes rated/related/reported
RCT: Randomized Controlled Trial
RoB: Risk of Bias
VBA: Visual Basic for Applications
