# Supplementary material for: Assessing the risk of performance and detection bias in Cochrane reviews as a joint domain is less accurate compared to two separate domains
Source: BMC Med Res Methodol. 2021 Jul 18;21:149. doi: 10.1186/s12874-021-01339-1 (PMC8286598; doi:10.1186/s12874-021-01339-1)
Supplement: Supplementary file 1 — Additional file 1. List of analyzed Cochrane systematic reviews and studies included. [file 12874_2021_1339_MOESM1_ESM.docx]

**Supplementary file 1:** List of analyzed Cochrane systematic reviews and studies included.

| **Study included in assessment (n=2800)** | **Cochrane review (n=170)** | **Number of assessed judgments (n=3169)** |
| --- | --- | --- |
| Candy 1995 | Azathioprine or 6-mercaptopurine for maintenance of remission in Crohn's disease | 1 |
| Cosnes 2013 | Azathioprine or 6-mercaptopurine for maintenance of remission in Crohn's disease | 1 |
| Lémann 2005 | Azathioprine or 6-mercaptopurine for maintenance of remission in Crohn's disease | 1 |
| Mantzaris 2004 | Azathioprine or 6-mercaptopurine for maintenance of remission in Crohn's disease | 1 |
| Mantzaris 2009 | Azathioprine or 6-mercaptopurine for maintenance of remission in Crohn's disease | 1 |
| Maté-Jiménez 2000 | Azathioprine or 6-mercaptopurine for maintenance of remission in Crohn's disease | 1 |
| O'Donoghue 1978 | Azathioprine or 6-mercaptopurine for maintenance of remission in Crohn's disease | 1 |
| Panes 2013 | Azathioprine or 6-mercaptopurine for maintenance of remission in Crohn's disease | 1 |
| Rosenberg 1975 | Azathioprine or 6-mercaptopurine for maintenance of remission in Crohn's disease | 1 |
| Summers 1979 | Azathioprine or 6-mercaptopurine for maintenance of remission in Crohn's disease | 1 |
| Willoughby 1971 | Azathioprine or 6-mercaptopurine for maintenance of remission in Crohn's disease | 1 |
| Ainsworth 2000 | Animal derived surfactant extract versus protein free synthetic surfactant for the prevention and treatment of respiratory distress syndrome | 1 |
| Alvarado 1993 | Animal derived surfactant extract versus protein free synthetic surfactant for the prevention and treatment of respiratory distress syndrome | 1 |
| daCosta 1999 | Animal derived surfactant extract versus protein free synthetic surfactant for the prevention and treatment of respiratory distress syndrome | 1 |
| Halahakoon 1999 | Animal derived surfactant extract versus protein free synthetic surfactant for the prevention and treatment of respiratory distress syndrome | 1 |
| Horbar 1993 | Animal derived surfactant extract versus protein free synthetic surfactant for the prevention and treatment of respiratory distress syndrome | 1 |
| Hudak 1996 | Animal derived surfactant extract versus protein free synthetic surfactant for the prevention and treatment of respiratory distress syndrome | 1 |
| Hudak 1997 | Animal derived surfactant extract versus protein free synthetic surfactant for the prevention and treatment of respiratory distress syndrome | 1 |
| Kukkonen 2000 | Animal derived surfactant extract versus protein free synthetic surfactant for the prevention and treatment of respiratory distress syndrome | 1 |
| Lloyd 1999 | Animal derived surfactant extract versus protein free synthetic surfactant for the prevention and treatment of respiratory distress syndrome | 1 |
| Modanlou 1997 | Animal derived surfactant extract versus protein free synthetic surfactant for the prevention and treatment of respiratory distress syndrome | 1 |
| Moya 2005 | Animal derived surfactant extract versus protein free synthetic surfactant for the prevention and treatment of respiratory distress syndrome | 1 |
| Murdoch 1998 | Animal derived surfactant extract versus protein free synthetic surfactant for the prevention and treatment of respiratory distress syndrome | 1 |
| Pearlman 1993 | Animal derived surfactant extract versus protein free synthetic surfactant for the prevention and treatment of respiratory distress syndrome | 1 |
| Sehgal 1994 | Animal derived surfactant extract versus protein free synthetic surfactant for the prevention and treatment of respiratory distress syndrome | 1 |
| VT Oxford 1996 | Animal derived surfactant extract versus protein free synthetic surfactant for the prevention and treatment of respiratory distress syndrome | 1 |
| Balaban 2008 | Discharge planning from hospital | 1 |
| Bolas 2004 | Discharge planning from hospital | 1 |
| Eggink 2010 | Discharge planning from hospital | 1 |
| Evans 1993 | Discharge planning from hospital | 1 |
| Farris 2014 | Discharge planning from hospital | 1 |
| Gillespie 2009 | Discharge planning from hospital | 1 |
| Goldman 2014 | Discharge planning from hospital | 1 |
| Harrison 2002 | Discharge planning from hospital | 1 |
| Hendriksen 1990 | Discharge planning from hospital | 1 |
| Jack 2009 | Discharge planning from hospital | 1 |
| Kennedy 1987 | Discharge planning from hospital | 1 |
| Kripalani 2012 | Discharge planning from hospital | 1 |
| Lainscak 2013 | Discharge planning from hospital | 1 |
| Laramee 2003 | Discharge planning from hospital | 1 |
| Legrain 2011 | Discharge planning from hospital | 1 |
| Lin 2009 | Discharge planning from hospital | 1 |
| Lindpaintner 2013 | Discharge planning from hospital | 1 |
| Moher 1992 | Discharge planning from hospital | 1 |
| Naji 1999 | Discharge planning from hospital | 1 |
| Naughton 1994 | Discharge planning from hospital | 1 |
| Naylor 1994 | Discharge planning from hospital | 1 |
| Nazareth 2001 | Discharge planning from hospital | 1 |
| Pardessus 2002 | Discharge planning from hospital | 1 |
| Parfrey 1994 | Discharge planning from hospital | 1 |
| Preen 2005 | Discharge planning from hospital | 1 |
| Rich 1993 | Discharge planning from hospital | 1 |
| Rich 1995 | Discharge planning from hospital | 1 |
| Shaw 2000 | Discharge planning from hospital | 1 |
| Sulch 2000 | Discharge planning from hospital | 1 |
| Weinberger 1996 | Discharge planning from hospital | 1 |
| Arici 1994 | Assisted reproductive technologies for male subfertility | 1 |
| Bensdorp 2015 | Assisted reproductive technologies for male subfertility | 1 |
| Cohlen 1998 | Assisted reproductive technologies for male subfertility | 1 |
| Francavilla 2009 | Assisted reproductive technologies for male subfertility | 1 |
| Goverde 2000 | Assisted reproductive technologies for male subfertility | 1 |
| Gregoriou 1996 | Assisted reproductive technologies for male subfertility | 1 |
| Guzick 1999 | Assisted reproductive technologies for male subfertility | 1 |
| Kerin 1984 | Assisted reproductive technologies for male subfertility | 1 |
| Melis 1995 | Assisted reproductive technologies for male subfertility | 1 |
| Nan 1994 | Assisted reproductive technologies for male subfertility | 1 |
| Alderman 2006 | Deworming drugs for soil-transmitted intestinal worms in children: effects on nutritional indicators, haemoglobin, and school performance | 1 |
| Awasthi 1995 | Deworming drugs for soil-transmitted intestinal worms in children: effects on nutritional indicators, haemoglobin, and school performance | 1 |
| Awasthi 2000 | Deworming drugs for soil-transmitted intestinal worms in children: effects on nutritional indicators, haemoglobin, and school performance | 1 |
| Awasthi 2001 | Deworming drugs for soil-transmitted intestinal worms in children: effects on nutritional indicators, haemoglobin, and school performance | 1 |
| Awasthi 2013 | Deworming drugs for soil-transmitted intestinal worms in children: effects on nutritional indicators, haemoglobin, and school performance | 1 |
| Beach 1999 | Deworming drugs for soil-transmitted intestinal worms in children: effects on nutritional indicators, haemoglobin, and school performance | 1 |
| Donnen 1998 | Deworming drugs for soil-transmitted intestinal worms in children: effects on nutritional indicators, haemoglobin, and school performance | 1 |
| Dossa 2001 | Deworming drugs for soil-transmitted intestinal worms in children: effects on nutritional indicators, haemoglobin, and school performance | 1 |
| Fox 2005 | Deworming drugs for soil-transmitted intestinal worms in children: effects on nutritional indicators, haemoglobin, and school performance | 1 |
| Freij 1979 | Deworming drugs for soil-transmitted intestinal worms in children: effects on nutritional indicators, haemoglobin, and school performance | 1 |
| Freij 1979 | Deworming drugs for soil-transmitted intestinal worms in children: effects on nutritional indicators, haemoglobin, and school performance | 1 |
| Garg 2002 | Deworming drugs for soil-transmitted intestinal worms in children: effects on nutritional indicators, haemoglobin, and school performance | 1 |
| Goto 2009 | Deworming drugs for soil-transmitted intestinal worms in children: effects on nutritional indicators, haemoglobin, and school performance | 1 |
| Greenberg 1981 | Deworming drugs for soil-transmitted intestinal worms in children: effects on nutritional indicators, haemoglobin, and school performance | 1 |
| Hadju 1996 | Deworming drugs for soil-transmitted intestinal worms in children: effects on nutritional indicators, haemoglobin, and school performance | 1 |
| Hadju 1997 | Deworming drugs for soil-transmitted intestinal worms in children: effects on nutritional indicators, haemoglobin, and school performance | 1 |
| Hall 2006 | Deworming drugs for soil-transmitted intestinal worms in children: effects on nutritional indicators, haemoglobin, and school performance | 1 |
| Kirwan 2010 | Deworming drugs for soil-transmitted intestinal worms in children: effects on nutritional indicators, haemoglobin, and school performance | 1 |
| Kloetzel 1982 | Deworming drugs for soil-transmitted intestinal worms in children: effects on nutritional indicators, haemoglobin, and school performance | 1 |
| Koroma 1996 | Deworming drugs for soil-transmitted intestinal worms in children: effects on nutritional indicators, haemoglobin, and school performance | 1 |
| Kruger 1996 | Deworming drugs for soil-transmitted intestinal worms in children: effects on nutritional indicators, haemoglobin, and school performance | 1 |
| Kvalsvig 1991 | Deworming drugs for soil-transmitted intestinal worms in children: effects on nutritional indicators, haemoglobin, and school performance | 1 |
| Lai 1995 | Deworming drugs for soil-transmitted intestinal worms in children: effects on nutritional indicators, haemoglobin, and school performance | 1 |
| Le Huong 2007 | Deworming drugs for soil-transmitted intestinal worms in children: effects on nutritional indicators, haemoglobin, and school performance | 1 |
| Michaelsen 1985 | Deworming drugs for soil-transmitted intestinal worms in children: effects on nutritional indicators, haemoglobin, and school performance | 1 |
| Miguel 2004 | Deworming drugs for soil-transmitted intestinal worms in children: effects on nutritional indicators, haemoglobin, and school performance | 1 |
| Ndibazza 2012 | Deworming drugs for soil-transmitted intestinal worms in children: effects on nutritional indicators, haemoglobin, and school performance | 1 |
| Nga 2009 | Deworming drugs for soil-transmitted intestinal worms in children: effects on nutritional indicators, haemoglobin, and school performance | 1 |
| Nokes 1992 | Deworming drugs for soil-transmitted intestinal worms in children: effects on nutritional indicators, haemoglobin, and school performance | 1 |
| Olds 1999 | Deworming drugs for soil-transmitted intestinal worms in children: effects on nutritional indicators, haemoglobin, and school performance | 1 |
| Palupi 1997 | Deworming drugs for soil-transmitted intestinal worms in children: effects on nutritional indicators, haemoglobin, and school performance | 1 |
| Rousham 1994 | Deworming drugs for soil-transmitted intestinal worms in children: effects on nutritional indicators, haemoglobin, and school performance | 1 |
| Sarkar 2002 | Deworming drugs for soil-transmitted intestinal worms in children: effects on nutritional indicators, haemoglobin, and school performance | 1 |
| Simeon 1995 | Deworming drugs for soil-transmitted intestinal worms in children: effects on nutritional indicators, haemoglobin, and school performance | 1 |
| Solon 2003 | Deworming drugs for soil-transmitted intestinal worms in children: effects on nutritional indicators, haemoglobin, and school performance | 1 |
| Stephenson 1989 | Deworming drugs for soil-transmitted intestinal worms in children: effects on nutritional indicators, haemoglobin, and school performance | 1 |
| Stephenson 1993 | Deworming drugs for soil-transmitted intestinal worms in children: effects on nutritional indicators, haemoglobin, and school performance | 1 |
| Stoltzfus 1997 | Deworming drugs for soil-transmitted intestinal worms in children: effects on nutritional indicators, haemoglobin, and school performance | 1 |
| Stoltzfus 2001 | Deworming drugs for soil-transmitted intestinal worms in children: effects on nutritional indicators, haemoglobin, and school performance | 1 |
| Sur 2005 | Deworming drugs for soil-transmitted intestinal worms in children: effects on nutritional indicators, haemoglobin, and school performance | 1 |
| Tee 2013 | Deworming drugs for soil-transmitted intestinal worms in children: effects on nutritional indicators, haemoglobin, and school performance | 1 |
| Watkins 1996 | Deworming drugs for soil-transmitted intestinal worms in children: effects on nutritional indicators, haemoglobin, and school performance | 1 |
| Willett 1979 | Deworming drugs for soil-transmitted intestinal worms in children: effects on nutritional indicators, haemoglobin, and school performance | 1 |
| Wiria 2013 | Deworming drugs for soil-transmitted intestinal worms in children: effects on nutritional indicators, haemoglobin, and school performance | 1 |
| Yap 2014 | Deworming drugs for soil-transmitted intestinal worms in children: effects on nutritional indicators, haemoglobin, and school performance | 1 |
| Auerbach 1985 | Oral steroids for long-term use in cystic fibrosis | 1 |
| Eigen 1995 | Oral steroids for long-term use in cystic fibrosis | 1 |
| Greally 1994 | Oral steroids for long-term use in cystic fibrosis | 1 |
| ACTNoW 2011 | Speech and language therapy for aphasia following stroke | 1 |
| B.A.Bar 2011 | Speech and language therapy for aphasia following stroke | 1 |
| B.A.Bar 2011 | Speech and language therapy for aphasia following stroke | 1 |
| Bakheit 2007 | Speech and language therapy for aphasia following stroke | 1 |
| CACTUS 2013 | Speech and language therapy for aphasia following stroke | 1 |
| Conklyn 2012 | Speech and language therapy for aphasia following stroke | 1 |
| Crerar 1996 | Speech and language therapy for aphasia following stroke | 1 |
| Crosson 2014 | Speech and language therapy for aphasia following stroke | 1 |
| David 1982 | Speech and language therapy for aphasia following stroke | 1 |
| Denes 1996 | Speech and language therapy for aphasia following stroke | 1 |
| Di Carlo 1980 | Speech and language therapy for aphasia following stroke | 1 |
| Doesborgh 2004 | Speech and language therapy for aphasia following stroke | 1 |
| Drummond 1981 | Speech and language therapy for aphasia following stroke | 1 |
| Elman 1999 | Speech and language therapy for aphasia following stroke | 1 |
| FUATAC 0000 | Speech and language therapy for aphasia following stroke | 1 |
| Hinckley 2001 | Speech and language therapy for aphasia following stroke | 1 |
| Katz 1997 | Speech and language therapy for aphasia following stroke | 1 |
| Katz 1997 | Speech and language therapy for aphasia following stroke | 1 |
| Laska 2011 | Speech and language therapy for aphasia following stroke | 1 |
| Leal 1993 | Speech and language therapy for aphasia following stroke | 1 |
| Lincoln 1982 | Speech and language therapy for aphasia following stroke | 1 |
| Lincoln 1982 | Speech and language therapy for aphasia following stroke | 1 |
| Lincoln 1982 | Speech and language therapy for aphasia following stroke | 1 |
| Lincoln 1984 | Speech and language therapy for aphasia following stroke | 1 |
| Lincoln 1984 | Speech and language therapy for aphasia following stroke | 1 |
| Liu 2006 | Speech and language therapy for aphasia following stroke | 1 |
| Lyon 1997 | Speech and language therapy for aphasia following stroke | 1 |
| MacKay 1988 | Speech and language therapy for aphasia following stroke | 1 |
| Mattioli 2014 | Speech and language therapy for aphasia following stroke | 1 |
| Meikle 1979 | Speech and language therapy for aphasia following stroke | 1 |
| Meinzer 2007 | Speech and language therapy for aphasia following stroke | 1 |
| MIT 2014 | Speech and language therapy for aphasia following stroke | 1 |
| MIT 2014 | Speech and language therapy for aphasia following stroke | 1 |
| NARNIA 2013 | Speech and language therapy for aphasia following stroke | 1 |
| ORLA 2006 | Speech and language therapy for aphasia following stroke | 1 |
| ORLA 2010 | Speech and language therapy for aphasia following stroke | 1 |
| Prins 1989 | Speech and language therapy for aphasia following stroke | 1 |
| Pulvermuller 2001 | Speech and language therapy for aphasia following stroke | 1 |
| RATS 0000 | Speech and language therapy for aphasia following stroke | 1 |
| RATS-2 0000 | Speech and language therapy for aphasia following stroke | 1 |
| Rochon 2005 | Speech and language therapy for aphasia following stroke | 1 |
| SEMaFORE 0000 | Speech and language therapy for aphasia following stroke | 1 |
| Shewan 1984 | Speech and language therapy for aphasia following stroke | 1 |
| Shewan 1984 | Speech and language therapy for aphasia following stroke | 1 |
| Shewan 1984 | Speech and language therapy for aphasia following stroke | 1 |
| Sickert 2014 | Speech and language therapy for aphasia following stroke | 1 |
| Smania 2006 | Speech and language therapy for aphasia following stroke | 1 |
| Smith 1981 | Speech and language therapy for aphasia following stroke | 1 |
| Smith 1981 | Speech and language therapy for aphasia following stroke | 1 |
| Smith 1981 | Speech and language therapy for aphasia following stroke | 1 |
| SP-I-RiT 0000 | Speech and language therapy for aphasia following stroke | 1 |
| Szaflarski 2014 | Speech and language therapy for aphasia following stroke | 1 |
| Van Steenbrugge 1981 | Speech and language therapy for aphasia following stroke | 1 |
| Varley 2016 | Speech and language therapy for aphasia following stroke | 1 |
| Varley 2016 | Speech and language therapy for aphasia following stroke | 1 |
| VERSE I 0000 | Speech and language therapy for aphasia following stroke | 1 |
| VERSE II 0000 | Speech and language therapy for aphasia following stroke | 1 |
| Wertz 1981 | Speech and language therapy for aphasia following stroke | 1 |
| Wertz 1986 | Speech and language therapy for aphasia following stroke | 1 |
| Wertz 1986 | Speech and language therapy for aphasia following stroke | 1 |
| Wertz 1986 | Speech and language therapy for aphasia following stroke | 1 |
| Wilssens 2015 | Speech and language therapy for aphasia following stroke | 1 |
| Woolf 2015 | Speech and language therapy for aphasia following stroke | 1 |
| Woolf 2015 | Speech and language therapy for aphasia following stroke | 1 |
| Woolf 2015 | Speech and language therapy for aphasia following stroke | 1 |
| Wu 2004 | Speech and language therapy for aphasia following stroke | 1 |
| Wu 2013 | Speech and language therapy for aphasia following stroke | 1 |
| Xie 2002 | Speech and language therapy for aphasia following stroke | 1 |
| Yao 2005 | Speech and language therapy for aphasia following stroke | 1 |
| Yao 2005 | Speech and language therapy for aphasia following stroke | 1 |
| Yao 2005 | Speech and language therapy for aphasia following stroke | 1 |
| Zhang 2007 | Speech and language therapy for aphasia following stroke | 1 |
| Zhang 2007 | Speech and language therapy for aphasia following stroke | 1 |
| Zhao 2000 | Speech and language therapy for aphasia following stroke | 1 |
| Agorastides 2007 | Interventions for treating proximal humeral fractures in adults | 2 |
| Bertoft 1984 | Interventions for treating proximal humeral fractures in adults | 2 |
| Boons 2012 | Interventions for treating proximal humeral fractures in adults | 2 |
| Buecking 2014 | Interventions for treating proximal humeral fractures in adults | 2 |
| Cai 2012 | Interventions for treating proximal humeral fractures in adults | 2 |
| Fialka 2008 | Interventions for treating proximal humeral fractures in adults | 2 |
| Fjalestad 2010 | Interventions for treating proximal humeral fractures in adults | 2 |
| Hodgson 2003 | Interventions for treating proximal humeral fractures in adults | 2 |
| Hoellen 1997 | Interventions for treating proximal humeral fractures in adults | 2 |
| Kristiansen 1988 | Interventions for treating proximal humeral fractures in adults | 2 |
| Kristiansen 1989 | Interventions for treating proximal humeral fractures in adults | 2 |
| Lefevre-Colau 2007 | Interventions for treating proximal humeral fractures in adults | 2 |
| Livesley 1992 | Interventions for treating proximal humeral fractures in adults | 2 |
| Lopiz 2014 | Interventions for treating proximal humeral fractures in adults | 2 |
| Lundberg 1979 | Interventions for treating proximal humeral fractures in adults | 2 |
| Ockert 2010 | Interventions for treating proximal humeral fractures in adults | 2 |
| Olerud 2011 | Interventions for treating proximal humeral fractures in adults | 2 |
| Olerud 2011 | Interventions for treating proximal humeral fractures in adults | 2 |
| ProFHER 2015 | Interventions for treating proximal humeral fractures in adults | 2 |
| Revay 1992 | Interventions for treating proximal humeral fractures in adults | 2 |
| Rommens 1993 | Interventions for treating proximal humeral fractures in adults | 2 |
| Sebastiá-Forcada 2014 | Interventions for treating proximal humeral fractures in adults | 2 |
| Smejkal 2011 | Interventions for treating proximal humeral fractures in adults | 2 |
| Soliman 2013 | Interventions for treating proximal humeral fractures in adults | 2 |
| Stableforth 1984 | Interventions for treating proximal humeral fractures in adults | 2 |
| Torrens 2012 | Interventions for treating proximal humeral fractures in adults | 2 |
| Voigt 2011 | Interventions for treating proximal humeral fractures in adults | 2 |
| Wirbel 1999 | Interventions for treating proximal humeral fractures in adults | 2 |
| Zhang 2011 | Interventions for treating proximal humeral fractures in adults | 2 |
| Zhu 2011 | Interventions for treating proximal humeral fractures in adults | 2 |
| Zyto 1997 | Interventions for treating proximal humeral fractures in adults | 2 |
| Keszler 1991 | Rescue high-frequency jet ventilation versus conventional ventilation for severe pulmonary dysfunction in preterm infants | 1 |
| Andreoli 1987 | Oral 5-aminosalicylic acid for induction of remission in ulcerative colitis | 1 |
| Bresci 1990 | Oral 5-aminosalicylic acid for induction of remission in ulcerative colitis | 1 |
| Cai 2001 | Oral 5-aminosalicylic acid for induction of remission in ulcerative colitis | 1 |
| D'Haens 2006 | Oral 5-aminosalicylic acid for induction of remission in ulcerative colitis | 1 |
| Ewe 1988 | Oral 5-aminosalicylic acid for induction of remission in ulcerative colitis | 1 |
| Farup 2001 | Oral 5-aminosalicylic acid for induction of remission in ulcerative colitis | 1 |
| Feagan 2013 | Oral 5-aminosalicylic acid for induction of remission in ulcerative colitis | 1 |
| Feurle 1989 | Oral 5-aminosalicylic acid for induction of remission in ulcerative colitis | 1 |
| Fleig 1988 | Oral 5-aminosalicylic acid for induction of remission in ulcerative colitis | 1 |
| Flourie 2013 | Oral 5-aminosalicylic acid for induction of remission in ulcerative colitis | 1 |
| Forbes 2005 | Oral 5-aminosalicylic acid for induction of remission in ulcerative colitis | 1 |
| Gibson 2006 | Oral 5-aminosalicylic acid for induction of remission in ulcerative colitis | 1 |
| Good 1992 | Oral 5-aminosalicylic acid for induction of remission in ulcerative colitis | 1 |
| Green 1998 | Oral 5-aminosalicylic acid for induction of remission in ulcerative colitis | 1 |
| Green 2002 | Oral 5-aminosalicylic acid for induction of remission in ulcerative colitis | 1 |
| Hanauer 1993 | Oral 5-aminosalicylic acid for induction of remission in ulcerative colitis | 1 |
| Hanauer 1996 | Oral 5-aminosalicylic acid for induction of remission in ulcerative colitis | 1 |
| Hanauer 2005 | Oral 5-aminosalicylic acid for induction of remission in ulcerative colitis | 1 |
| Hanauer 2007 | Oral 5-aminosalicylic acid for induction of remission in ulcerative colitis | 1 |
| Hetzel 1986 | Oral 5-aminosalicylic acid for induction of remission in ulcerative colitis | 1 |
| Hiwatashi 2011 | Oral 5-aminosalicylic acid for induction of remission in ulcerative colitis | 1 |
| Ito 2010 | Oral 5-aminosalicylic acid for induction of remission in ulcerative colitis | 1 |
| Jiang 2004 | Oral 5-aminosalicylic acid for induction of remission in ulcerative colitis | 1 |
| Kamm 2007 | Oral 5-aminosalicylic acid for induction of remission in ulcerative colitis | 1 |
| Kruis 1998 | Oral 5-aminosalicylic acid for induction of remission in ulcerative colitis | 1 |
| Kruis 2003 | Oral 5-aminosalicylic acid for induction of remission in ulcerative colitis | 1 |
| Kruis 2009 | Oral 5-aminosalicylic acid for induction of remission in ulcerative colitis | 1 |
| Levine 2002 | Oral 5-aminosalicylic acid for induction of remission in ulcerative colitis | 1 |
| Lichtenstein 2007 | Oral 5-aminosalicylic acid for induction of remission in ulcerative colitis | 1 |
| Maier 1985 | Oral 5-aminosalicylic acid for induction of remission in ulcerative colitis | 1 |
| Mansfield 2002 | Oral 5-aminosalicylic acid for induction of remission in ulcerative colitis | 1 |
| Marakhouski 2005 | Oral 5-aminosalicylic acid for induction of remission in ulcerative colitis | 1 |
| Miglioli 1990 | Oral 5-aminosalicylic acid for induction of remission in ulcerative colitis | 1 |
| Mihas 1988 | Oral 5-aminosalicylic acid for induction of remission in ulcerative colitis | 1 |
| Munakata 1995 | Oral 5-aminosalicylic acid for induction of remission in ulcerative colitis | 1 |
| Pontes 2014 | Oral 5-aminosalicylic acid for induction of remission in ulcerative colitis | 1 |
| Pruitt 2002 | Oral 5-aminosalicylic acid for induction of remission in ulcerative colitis | 1 |
| Qian 2004 | Oral 5-aminosalicylic acid for induction of remission in ulcerative colitis | 1 |
| Rachmilewitz 1989 | Oral 5-aminosalicylic acid for induction of remission in ulcerative colitis | 1 |
| Raedler 2004 | Oral 5-aminosalicylic acid for induction of remission in ulcerative colitis | 1 |
| Rao 1989 | Oral 5-aminosalicylic acid for induction of remission in ulcerative colitis | 1 |
| Rijk 1991 | Oral 5-aminosalicylic acid for induction of remission in ulcerative colitis | 1 |
| Riley 1988 | Oral 5-aminosalicylic acid for induction of remission in ulcerative colitis | 1 |
| Robinson 1988 | Oral 5-aminosalicylic acid for induction of remission in ulcerative colitis | 1 |
| Sandborn 2009 | Oral 5-aminosalicylic acid for induction of remission in ulcerative colitis | 1 |
| Sandborn 2012 | Oral 5-aminosalicylic acid for induction of remission in ulcerative colitis | 1 |
| Scherl 2009 | Oral 5-aminosalicylic acid for induction of remission in ulcerative colitis | 1 |
| Schroeder 1987 | Oral 5-aminosalicylic acid for induction of remission in ulcerative colitis | 1 |
| Sninsky 1991 | Oral 5-aminosalicylic acid for induction of remission in ulcerative colitis | 1 |
| Sutherland 1990 | Oral 5-aminosalicylic acid for induction of remission in ulcerative colitis | 1 |
| Tursi 2004 | Oral 5-aminosalicylic acid for induction of remission in ulcerative colitis | 1 |
| Willoughby 1988 | Oral 5-aminosalicylic acid for induction of remission in ulcerative colitis | 1 |
| Zinberg 1990 | Oral 5-aminosalicylic acid for induction of remission in ulcerative colitis | 1 |
| Andreoli 1987 | Oral 5-aminosalicylic acid for maintenance of remission in ulcerative colitis | 1 |
| Ardizzone 1995 | Oral 5-aminosalicylic acid for maintenance of remission in ulcerative colitis | 1 |
| Ardizzone 1999 | Oral 5-aminosalicylic acid for maintenance of remission in ulcerative colitis | 1 |
| Courtney 1992 | Oral 5-aminosalicylic acid for maintenance of remission in ulcerative colitis | 1 |
| D'Haens 2012 | Oral 5-aminosalicylic acid for maintenance of remission in ulcerative colitis | 1 |
| Deventer 2001 | Oral 5-aminosalicylic acid for maintenance of remission in ulcerative colitis | 1 |
| Dew 1983 | Oral 5-aminosalicylic acid for maintenance of remission in ulcerative colitis | 1 |
| Dignass 2009 | Oral 5-aminosalicylic acid for maintenance of remission in ulcerative colitis | 1 |
| Fockens 1995 | Oral 5-aminosalicylic acid for maintenance of remission in ulcerative colitis | 1 |
| Giaffer 1992 | Oral 5-aminosalicylic acid for maintenance of remission in ulcerative colitis | 1 |
| Green 1992 | Oral 5-aminosalicylic acid for maintenance of remission in ulcerative colitis | 1 |
| Green 1998 | Oral 5-aminosalicylic acid for maintenance of remission in ulcerative colitis | 1 |
| Hanauer 1996 | Oral 5-aminosalicylic acid for maintenance of remission in ulcerative colitis | 1 |
| Hawkey 1997 | Oral 5-aminosalicylic acid for maintenance of remission in ulcerative colitis | 1 |
| Hawthorne 2012 | Oral 5-aminosalicylic acid for maintenance of remission in ulcerative colitis | 1 |
| Ireland 1988 | Oral 5-aminosalicylic acid for maintenance of remission in ulcerative colitis | 1 |
| Ito 2010 | Oral 5-aminosalicylic acid for maintenance of remission in ulcerative colitis | 1 |
| Kamm 2008 | Oral 5-aminosalicylic acid for maintenance of remission in ulcerative colitis | 1 |
| Kane 2003 | Oral 5-aminosalicylic acid for maintenance of remission in ulcerative colitis | 1 |
| Kane 2008 | Oral 5-aminosalicylic acid for maintenance of remission in ulcerative colitis | 1 |
| Kiilerich 1992 | Oral 5-aminosalicylic acid for maintenance of remission in ulcerative colitis | 1 |
| Kruis 1995 | Oral 5-aminosalicylic acid for maintenance of remission in ulcerative colitis | 1 |
| Kruis 2001 | Oral 5-aminosalicylic acid for maintenance of remission in ulcerative colitis | 1 |
| Kruis 2011 | Oral 5-aminosalicylic acid for maintenance of remission in ulcerative colitis | 1 |
| Lichtenstein 2010 | Oral 5-aminosalicylic acid for maintenance of remission in ulcerative colitis | 1 |
| Mahmud 2002 | Oral 5-aminosalicylic acid for maintenance of remission in ulcerative colitis | 1 |
| McIntyre 1988 | Oral 5-aminosalicylic acid for maintenance of remission in ulcerative colitis | 1 |
| Miner 1995 | Oral 5-aminosalicylic acid for maintenance of remission in ulcerative colitis | 1 |
| Mulder 1988 | Oral 5-aminosalicylic acid for maintenance of remission in ulcerative colitis | 1 |
| Nilsson 1995 | Oral 5-aminosalicylic acid for maintenance of remission in ulcerative colitis | 1 |
| Paoluzi 2005 | Oral 5-aminosalicylic acid for maintenance of remission in ulcerative colitis | 1 |
| Pica 2012 | Oral 5-aminosalicylic acid for maintenance of remission in ulcerative colitis | 1 |
| Prantera 2009 | Oral 5-aminosalicylic acid for maintenance of remission in ulcerative colitis | 1 |
| Rijk 1992 | Oral 5-aminosalicylic acid for maintenance of remission in ulcerative colitis | 1 |
| Riley 1988 | Oral 5-aminosalicylic acid for maintenance of remission in ulcerative colitis | 1 |
| Rutgeerts 1989 | Oral 5-aminosalicylic acid for maintenance of remission in ulcerative colitis | 1 |
| Sandberg-Gertzen 1986 | Oral 5-aminosalicylic acid for maintenance of remission in ulcerative colitis | 1 |
| Sandborn 2010 | Oral 5-aminosalicylic acid for maintenance of remission in ulcerative colitis | 1 |
| Travis 1994 | Oral 5-aminosalicylic acid for maintenance of remission in ulcerative colitis | 1 |
| Watanabe 2013 | Oral 5-aminosalicylic acid for maintenance of remission in ulcerative colitis | 1 |
| Wright 1993 | Oral 5-aminosalicylic acid for maintenance of remission in ulcerative colitis | 1 |
| Fenton 1979 | Crisis intervention for people with severe mental illnesses | 1 |
| Fenton 1998 | Crisis intervention for people with severe mental illnesses | 1 |
| Hoult 1983 | Crisis intervention for people with severe mental illnesses | 1 |
| Howard 2010 | Crisis intervention for people with severe mental illnesses | 1 |
| Johnson 2005 | Crisis intervention for people with severe mental illnesses | 1 |
| Muijen 1992 | Crisis intervention for people with severe mental illnesses | 1 |
| Pasamanick 1964 | Crisis intervention for people with severe mental illnesses | 1 |
| Stein 1975 | Crisis intervention for people with severe mental illnesses | 1 |
| Adde 2004 | Dornase alfa for cystic fibrosis | 1 |
| Amin 2011 | Dornase alfa for cystic fibrosis | 1 |
| Ballmann 2002 | Dornase alfa for cystic fibrosis | 1 |
| Castile 2009 | Dornase alfa for cystic fibrosis | 1 |
| Dodd 2000 | Dornase alfa for cystic fibrosis | 1 |
| Frederiksen 2006 | Dornase alfa for cystic fibrosis | 1 |
| Fuchs 1994 | Dornase alfa for cystic fibrosis | 1 |
| Laube 1996 | Dornase alfa for cystic fibrosis | 1 |
| McCoy 1996 | Dornase alfa for cystic fibrosis | 1 |
| Minasian 2010 | Dornase alfa for cystic fibrosis | 1 |
| Paul 2004 | Dornase alfa for cystic fibrosis | 1 |
| Quan 2001 | Dornase alfa for cystic fibrosis | 1 |
| Ramsey 1993 | Dornase alfa for cystic fibrosis | 1 |
| Ranasinha 1993 | Dornase alfa for cystic fibrosis | 1 |
| Robinson 2000 | Dornase alfa for cystic fibrosis | 1 |
| Robinson 2005 | Dornase alfa for cystic fibrosis | 1 |
| Shah 1995 | Dornase alfa for cystic fibrosis | 1 |
| Suri 2001 | Dornase alfa for cystic fibrosis | 1 |
| Wilmott 1996 | Dornase alfa for cystic fibrosis | 1 |
| Alkharfy 2014 | Topical emollient for preventing infection in preterm infants | 1 |
| Arora 2005 | Topical emollient for preventing infection in preterm infants | 1 |
| Darmstadt 2004 | Topical emollient for preventing infection in preterm infants | 1 |
| Darmstadt 2005 | Topical emollient for preventing infection in preterm infants | 1 |
| Edwards 2004 | Topical emollient for preventing infection in preterm infants | 1 |
| Erdemir 2014 | Topical emollient for preventing infection in preterm infants | 1 |
| Fallah 2013 | Topical emollient for preventing infection in preterm infants | 1 |
| Farhat 2010 | Topical emollient for preventing infection in preterm infants | 1 |
| Kanti 2014 | Topical emollient for preventing infection in preterm infants | 1 |
| Kiechl-Kohlendorfer 2008 | Topical emollient for preventing infection in preterm infants | 1 |
| Kumar 2013 | Topical emollient for preventing infection in preterm infants | 1 |
| Lane 1993 | Topical emollient for preventing infection in preterm infants | 1 |
| Nopper 1996 | Topical emollient for preventing infection in preterm infants | 1 |
| Pabst 1999 | Topical emollient for preventing infection in preterm infants | 1 |
| Salam 2015 | Topical emollient for preventing infection in preterm infants | 1 |
| Sankaranarayanan 2005 | Topical emollient for preventing infection in preterm infants | 1 |
| Soriano 2000 | Topical emollient for preventing infection in preterm infants | 1 |
| Vaivre-Douret 2008 | Topical emollient for preventing infection in preterm infants | 1 |
| Brown 2004 | Treatment and prevention of pouchitis after ileal pouch-anal anastomosis for chronic ulcerative colitis | 1 |
| Gionchetti 2000 | Treatment and prevention of pouchitis after ileal pouch-anal anastomosis for chronic ulcerative colitis | 1 |
| Gionchetti 2003 | Treatment and prevention of pouchitis after ileal pouch-anal anastomosis for chronic ulcerative colitis | 1 |
| Ha 2010 | Treatment and prevention of pouchitis after ileal pouch-anal anastomosis for chronic ulcerative colitis | 1 |
| Isaacs 2007 | Treatment and prevention of pouchitis after ileal pouch-anal anastomosis for chronic ulcerative colitis | 1 |
| Joelsson 2001 | Treatment and prevention of pouchitis after ileal pouch-anal anastomosis for chronic ulcerative colitis | 1 |
| Kuisma 2003 | Treatment and prevention of pouchitis after ileal pouch-anal anastomosis for chronic ulcerative colitis | 1 |
| Mimura 2004 | Treatment and prevention of pouchitis after ileal pouch-anal anastomosis for chronic ulcerative colitis | 1 |
| Pronio 2008 | Treatment and prevention of pouchitis after ileal pouch-anal anastomosis for chronic ulcerative colitis | 1 |
| Sambuelli 2002 | Treatment and prevention of pouchitis after ileal pouch-anal anastomosis for chronic ulcerative colitis | 1 |
| Shen 2001 | Treatment and prevention of pouchitis after ileal pouch-anal anastomosis for chronic ulcerative colitis | 1 |
| Tremaine 1997 | Treatment and prevention of pouchitis after ileal pouch-anal anastomosis for chronic ulcerative colitis | 1 |
| Wischmeyer 1993 | Treatment and prevention of pouchitis after ileal pouch-anal anastomosis for chronic ulcerative colitis | 1 |
| Alecrim 2005 | Acupuncture for the prevention of episodic migraine | 1 |
| Alecrim 2006 | Acupuncture for the prevention of episodic migraine | 1 |
| Alecrim 2008 | Acupuncture for the prevention of episodic migraine | 1 |
| Allais 2002 | Acupuncture for the prevention of episodic migraine | 1 |
| Ceccherelli 1992 | Acupuncture for the prevention of episodic migraine | 1 |
| Diener 2006 | Acupuncture for the prevention of episodic migraine | 1 |
| Facco 2008 | Acupuncture for the prevention of episodic migraine | 1 |
| Facco 2013 | Acupuncture for the prevention of episodic migraine | 1 |
| Hesse 1994 | Acupuncture for the prevention of episodic migraine | 1 |
| Jena 2008 | Acupuncture for the prevention of episodic migraine | 1 |
| Li 2012 | Acupuncture for the prevention of episodic migraine | 1 |
| Linde K 2005 | Acupuncture for the prevention of episodic migraine | 1 |
| Linde M 2000 | Acupuncture for the prevention of episodic migraine | 1 |
| Linde M 2004 | Acupuncture for the prevention of episodic migraine | 1 |
| Streng 2006 | Acupuncture for the prevention of episodic migraine | 1 |
| Vickers 2004 | Acupuncture for the prevention of episodic migraine | 1 |
| Vincent 1989 | Acupuncture for the prevention of episodic migraine | 1 |
| Wallasch 2012 | Acupuncture for the prevention of episodic migraine | 1 |
| Wang 2015 | Acupuncture for the prevention of episodic migraine | 1 |
| Weinschütz 1993 | Acupuncture for the prevention of episodic migraine | 1 |
| Weinschütz 1994 | Acupuncture for the prevention of episodic migraine | 1 |
| Zhao 2014 | Acupuncture for the prevention of episodic migraine | 1 |
| Avasiloaiei 2013 | Prophylactic barbiturate use for the prevention of morbidity and mortality following perinatal asphyxia | 1 |
| Gathwala 2011 | Prophylactic barbiturate use for the prevention of morbidity and mortality following perinatal asphyxia | 1 |
| Goldberg 1986 | Prophylactic barbiturate use for the prevention of morbidity and mortality following perinatal asphyxia | 1 |
| Hall 1998 | Prophylactic barbiturate use for the prevention of morbidity and mortality following perinatal asphyxia | 1 |
| Ruth 1991 | Prophylactic barbiturate use for the prevention of morbidity and mortality following perinatal asphyxia | 1 |
| Singh 2004 | Prophylactic barbiturate use for the prevention of morbidity and mortality following perinatal asphyxia | 1 |
| Vargas-Origel 2004 | Prophylactic barbiturate use for the prevention of morbidity and mortality following perinatal asphyxia | 1 |
| Vela 1987 | Prophylactic barbiturate use for the prevention of morbidity and mortality following perinatal asphyxia | 1 |
| Velaphi 2013 | Prophylactic barbiturate use for the prevention of morbidity and mortality following perinatal asphyxia | 1 |
| Caple 2004 | Slow advancement of enteral feed volumes to prevent necrotising enterocolitis in very low birth weight infants | 1 |
| Jain 2016 | Slow advancement of enteral feed volumes to prevent necrotising enterocolitis in very low birth weight infants | 1 |
| Karagol 2013 | Slow advancement of enteral feed volumes to prevent necrotising enterocolitis in very low birth weight infants | 1 |
| Krishnamurthy 2010 | Slow advancement of enteral feed volumes to prevent necrotising enterocolitis in very low birth weight infants | 1 |
| Modi 2015 | Slow advancement of enteral feed volumes to prevent necrotising enterocolitis in very low birth weight infants | 1 |
| Raban 2014a 0000 | Slow advancement of enteral feed volumes to prevent necrotising enterocolitis in very low birth weight infants | 1 |
| Raban 2014b 0000 | Slow advancement of enteral feed volumes to prevent necrotising enterocolitis in very low birth weight infants | 1 |
| Rayyis 1999 | Slow advancement of enteral feed volumes to prevent necrotising enterocolitis in very low birth weight infants | 1 |
| Salhotra 2004 | Slow advancement of enteral feed volumes to prevent necrotising enterocolitis in very low birth weight infants | 1 |
| SIFT 2016 | Slow advancement of enteral feed volumes to prevent necrotising enterocolitis in very low birth weight infants | 1 |
| Han 1987 | Prophylactic nasal continuous positive airway pressure for preventing morbidity and mortality in very preterm infants | 1 |
| Sandri 2004 | Prophylactic nasal continuous positive airway pressure for preventing morbidity and mortality in very preterm infants | 1 |
| Morley 2008 | Prophylactic nasal continuous positive airway pressure for preventing morbidity and mortality in very preterm infants | 1 |
| Finer 2010 | Prophylactic nasal continuous positive airway pressure for preventing morbidity and mortality in very preterm infants | 1 |
| Dunn 2011 | Prophylactic nasal continuous positive airway pressure for preventing morbidity and mortality in very preterm infants | 1 |
| Tapia 2012 | Prophylactic nasal continuous positive airway pressure for preventing morbidity and mortality in very preterm infants | 1 |
| Gonçalves-Ferri 2014 | Prophylactic nasal continuous positive airway pressure for preventing morbidity and mortality in very preterm infants | 1 |
| Döring 2007 | Vaccines for preventing infection with Pseudomonas aeruginosa in cystic fibrosis | 1 |
| Lang 2004 | Vaccines for preventing infection with Pseudomonas aeruginosa in cystic fibrosis | 1 |
| Langford 1983 | Vaccines for preventing infection with Pseudomonas aeruginosa in cystic fibrosis | 1 |
| Braggion 1995 | Chest physiotherapy compared to no chest physiotherapy for cystic fibrosis | 1 |
| Elkins 2005 | Chest physiotherapy compared to no chest physiotherapy for cystic fibrosis | 1 |
| Falk 1993 | Chest physiotherapy compared to no chest physiotherapy for cystic fibrosis | 1 |
| Jarad 2010 | Chest physiotherapy compared to no chest physiotherapy for cystic fibrosis | 1 |
| Mortensen 1991 | Chest physiotherapy compared to no chest physiotherapy for cystic fibrosis | 1 |
| Pfleger 1992 | Chest physiotherapy compared to no chest physiotherapy for cystic fibrosis | 1 |
| Rossman 1982 | Chest physiotherapy compared to no chest physiotherapy for cystic fibrosis | 1 |
| van der Schans 1991 | Chest physiotherapy compared to no chest physiotherapy for cystic fibrosis | 1 |
| Kapoor 1998 | Corticosteroids for treating optic neuritis | 1 |
| Menon 2007 | Corticosteroids for treating optic neuritis | 1 |
| ONMRG 1999 | Corticosteroids for treating optic neuritis | 1 |
| ONTT 1992-2006 | Corticosteroids for treating optic neuritis | 1 |
| Sellebjerg 1999 | Corticosteroids for treating optic neuritis | 1 |
| Tübingen Study 1993 | Corticosteroids for treating optic neuritis | 1 |
| Bober-Olesiñska 2005 | Glutamine supplementation to prevent morbidity and mortality in preterm infants | 1 |
| Korkmaz 2007 | Glutamine supplementation to prevent morbidity and mortality in preterm infants | 1 |
| Lacey 1996 | Glutamine supplementation to prevent morbidity and mortality in preterm infants | 1 |
| Mohamad Ikram 2011 | Glutamine supplementation to prevent morbidity and mortality in preterm infants | 1 |
| Neu 1997 | Glutamine supplementation to prevent morbidity and mortality in preterm infants | 1 |
| Pawlik 2012 | Glutamine supplementation to prevent morbidity and mortality in preterm infants | 1 |
| Poindexter 2004 | Glutamine supplementation to prevent morbidity and mortality in preterm infants | 1 |
| Sevastiadou 2011 | Glutamine supplementation to prevent morbidity and mortality in preterm infants | 1 |
| Thompson 2003 | Glutamine supplementation to prevent morbidity and mortality in preterm infants | 1 |
| van den Berg 2005 | Glutamine supplementation to prevent morbidity and mortality in preterm infants | 1 |
| Vaughn 2003 | Glutamine supplementation to prevent morbidity and mortality in preterm infants | 1 |
| Wang 2010 | Glutamine supplementation to prevent morbidity and mortality in preterm infants | 1 |
| Konstan 1991 | Oral non-steroidal anti-inflammatory drug therapy for lung disease in cystic fibrosis | 1 |
| Konstan 1995 | Oral non-steroidal anti-inflammatory drug therapy for lung disease in cystic fibrosis | 1 |
| Lands 2007 | Oral non-steroidal anti-inflammatory drug therapy for lung disease in cystic fibrosis | 1 |
| Sordelli 1994 | Oral non-steroidal anti-inflammatory drug therapy for lung disease in cystic fibrosis | 1 |
| Bernard 1999 | Beta2-agonists for acute cough or a clinical diagnosis of acute bronchitis | 1 |
| Hueston 1991 | Beta2-agonists for acute cough or a clinical diagnosis of acute bronchitis | 1 |
| Hueston 1994 | Beta2-agonists for acute cough or a clinical diagnosis of acute bronchitis | 1 |
| Korppi 1991 | Beta2-agonists for acute cough or a clinical diagnosis of acute bronchitis | 1 |
| Littenberg 1996 | Beta2-agonists for acute cough or a clinical diagnosis of acute bronchitis | 1 |
| Melbye 1991 | Beta2-agonists for acute cough or a clinical diagnosis of acute bronchitis | 1 |
| Tukiainen 1986 | Beta2-agonists for acute cough or a clinical diagnosis of acute bronchitis | 1 |
| Andersen 1982 | Parenteral fluid regimens for improving functional outcome in people with acute stroke | 1 |
| Aronovitch 1999 | Parenteral fluid regimens for improving functional outcome in people with acute stroke | 1 |
| Bennett 1998 | Parenteral fluid regimens for improving functional outcome in people with acute stroke | 1 |
| Brienza 2010 | Parenteral fluid regimens for improving functional outcome in people with acute stroke | 1 |
| Cadue 2008 | Parenteral fluid regimens for improving functional outcome in people with acute stroke | 1 |
| Cavicchioli 2007 | Parenteral fluid regimens for improving functional outcome in people with acute stroke | 1 |
| Cobb 1997 | Parenteral fluid regimens for improving functional outcome in people with acute stroke | 1 |
| Collier 1996 | Parenteral fluid regimens for improving functional outcome in people with acute stroke | 1 |
| Conine 1990 | Parenteral fluid regimens for improving functional outcome in people with acute stroke | 1 |
| Conine 1993 | Parenteral fluid regimens for improving functional outcome in people with acute stroke | 1 |
| Conine 1994 | Parenteral fluid regimens for improving functional outcome in people with acute stroke | 1 |
| Cooper 1998 | Parenteral fluid regimens for improving functional outcome in people with acute stroke | 1 |
| Daechsel 1985 | Parenteral fluid regimens for improving functional outcome in people with acute stroke | 1 |
| Demarre 2012 | Parenteral fluid regimens for improving functional outcome in people with acute stroke | 1 |
| Donnelly 2011 | Parenteral fluid regimens for improving functional outcome in people with acute stroke | 1 |
| Economides 1995 | Parenteral fluid regimens for improving functional outcome in people with acute stroke | 1 |
| Ewing 1964 | Parenteral fluid regimens for improving functional outcome in people with acute stroke | 1 |
| Exton-Smith 1982 | Parenteral fluid regimens for improving functional outcome in people with acute stroke | 1 |
| Feuchtinger 2006 | Parenteral fluid regimens for improving functional outcome in people with acute stroke | 1 |
| Gebhardt 1996 | Parenteral fluid regimens for improving functional outcome in people with acute stroke | 1 |
| Gentilello 1988 | Parenteral fluid regimens for improving functional outcome in people with acute stroke | 1 |
| Geyer 2001 | Parenteral fluid regimens for improving functional outcome in people with acute stroke | 1 |
| Gilcreast 2005 | Parenteral fluid regimens for improving functional outcome in people with acute stroke | 1 |
| Goldstone 1982 | Parenteral fluid regimens for improving functional outcome in people with acute stroke | 1 |
| Gray 1994 | Parenteral fluid regimens for improving functional outcome in people with acute stroke | 1 |
| Gray 1998 | Parenteral fluid regimens for improving functional outcome in people with acute stroke | 1 |
| Gunningberg 2000 | Parenteral fluid regimens for improving functional outcome in people with acute stroke | 1 |
| Hampton 1997 | Parenteral fluid regimens for improving functional outcome in people with acute stroke | 1 |
| Hofman 1994 | Parenteral fluid regimens for improving functional outcome in people with acute stroke | 1 |
| Inman 1993 | Parenteral fluid regimens for improving functional outcome in people with acute stroke | 1 |
| Jolley 2004 | Parenteral fluid regimens for improving functional outcome in people with acute stroke | 1 |
| Kemp 1993 | Parenteral fluid regimens for improving functional outcome in people with acute stroke | 1 |
| Keogh 2001 | Parenteral fluid regimens for improving functional outcome in people with acute stroke | 1 |
| Laurent 1998 | Parenteral fluid regimens for improving functional outcome in people with acute stroke | 1 |
| Lazzara 1991 | Parenteral fluid regimens for improving functional outcome in people with acute stroke | 1 |
| Lim 1988 | Parenteral fluid regimens for improving functional outcome in people with acute stroke | 1 |
| McGowan 2000 | Parenteral fluid regimens for improving functional outcome in people with acute stroke | 1 |
| Mistiaen 2009 | Parenteral fluid regimens for improving functional outcome in people with acute stroke | 1 |
| Nixon 1998 | Parenteral fluid regimens for improving functional outcome in people with acute stroke | 1 |
| Nixon 2006 | Parenteral fluid regimens for improving functional outcome in people with acute stroke | 1 |
| Price 1999 | Parenteral fluid regimens for improving functional outcome in people with acute stroke | 1 |
| Ricci 2013 | Parenteral fluid regimens for improving functional outcome in people with acute stroke | 1 |
| Russell 2000 | Parenteral fluid regimens for improving functional outcome in people with acute stroke | 1 |
| Russell 2003 | Parenteral fluid regimens for improving functional outcome in people with acute stroke | 1 |
| Sanada 2003 | Parenteral fluid regimens for improving functional outcome in people with acute stroke | 1 |
| Santy 1994 | Parenteral fluid regimens for improving functional outcome in people with acute stroke | 1 |
| Schultz 1999 | Parenteral fluid regimens for improving functional outcome in people with acute stroke | 1 |
| Sideranko 1992 | Parenteral fluid regimens for improving functional outcome in people with acute stroke | 1 |
| Stapleton 1986 | Parenteral fluid regimens for improving functional outcome in people with acute stroke | 1 |
| Summer 1989 | Parenteral fluid regimens for improving functional outcome in people with acute stroke | 1 |
| Takala 1996 | Parenteral fluid regimens for improving functional outcome in people with acute stroke | 1 |
| Taylor 1999 | Parenteral fluid regimens for improving functional outcome in people with acute stroke | 1 |
| Theaker 2005 | Parenteral fluid regimens for improving functional outcome in people with acute stroke | 1 |
| Tymec 1997 | Parenteral fluid regimens for improving functional outcome in people with acute stroke | 1 |
| van Leen 2011 | Parenteral fluid regimens for improving functional outcome in people with acute stroke | 1 |
| Vanderwee 2005 | Parenteral fluid regimens for improving functional outcome in people with acute stroke | 1 |
| Vermette 2012 | Parenteral fluid regimens for improving functional outcome in people with acute stroke | 1 |
| Vyhlidal 1997 | Parenteral fluid regimens for improving functional outcome in people with acute stroke | 1 |
| Whitney 1984 | Parenteral fluid regimens for improving functional outcome in people with acute stroke | 1 |
| Al-Karaki 2011 | Gonadotrophin-releasing hormone antagonists for assisted reproductive technology | 1 |
| Albano 2000 | Gonadotrophin-releasing hormone antagonists for assisted reproductive technology | 1 |
| Anderson 2014 | Gonadotrophin-releasing hormone antagonists for assisted reproductive technology | 1 |
| Awata 2010 | Gonadotrophin-releasing hormone antagonists for assisted reproductive technology | 1 |
| Baart 2007 | Gonadotrophin-releasing hormone antagonists for assisted reproductive technology | 1 |
| Badrawi 2005 | Gonadotrophin-releasing hormone antagonists for assisted reproductive technology | 1 |
| Bahceci 2005 | Gonadotrophin-releasing hormone antagonists for assisted reproductive technology | 1 |
| Barmat 2005 | Gonadotrophin-releasing hormone antagonists for assisted reproductive technology | 1 |
| Brelik 2004 | Gonadotrophin-releasing hormone antagonists for assisted reproductive technology | 1 |
| Celik 2011 | Gonadotrophin-releasing hormone antagonists for assisted reproductive technology | 1 |
| Check 2004 | Gonadotrophin-releasing hormone antagonists for assisted reproductive technology | 1 |
| Cheung 2005 | Gonadotrophin-releasing hormone antagonists for assisted reproductive technology | 1 |
| Choi 2012 | Gonadotrophin-releasing hormone antagonists for assisted reproductive technology | 1 |
| Cota 2012 | Gonadotrophin-releasing hormone antagonists for assisted reproductive technology | 1 |
| Depalo 2009 | Gonadotrophin-releasing hormone antagonists for assisted reproductive technology | 1 |
| El Sahwi 2005 | Gonadotrophin-releasing hormone antagonists for assisted reproductive technology | 1 |
| Engmann 2008 | Gonadotrophin-releasing hormone antagonists for assisted reproductive technology | 1 |
| Euro Middle East 2001 | Gonadotrophin-releasing hormone antagonists for assisted reproductive technology | 1 |
| Euro Orgalutran 2000 | Gonadotrophin-releasing hormone antagonists for assisted reproductive technology | 1 |
| Ferrari 2006 | Gonadotrophin-releasing hormone antagonists for assisted reproductive technology | 1 |
| Ferrero 2010 | Gonadotrophin-releasing hormone antagonists for assisted reproductive technology | 1 |
| Firouzabadi 2010 | Gonadotrophin-releasing hormone antagonists for assisted reproductive technology | 1 |
| Fluker 2001 | Gonadotrophin-releasing hormone antagonists for assisted reproductive technology | 1 |
| Franco 2003 | Gonadotrophin-releasing hormone antagonists for assisted reproductive technology | 1 |
| Friedler 2003 | Gonadotrophin-releasing hormone antagonists for assisted reproductive technology | 1 |
| Gizzo 2014 | Gonadotrophin-releasing hormone antagonists for assisted reproductive technology | 1 |
| Haydardedeoglu 2012 | Gonadotrophin-releasing hormone antagonists for assisted reproductive technology | 1 |
| Heijnen 2007 | Gonadotrophin-releasing hormone antagonists for assisted reproductive technology | 1 |
| Hershko Klement 2015 | Gonadotrophin-releasing hormone antagonists for assisted reproductive technology | 1 |
| Hohmann 2003 | Gonadotrophin-releasing hormone antagonists for assisted reproductive technology | 1 |
| Hoseini 2014 | Gonadotrophin-releasing hormone antagonists for assisted reproductive technology | 1 |
| Hosseini 2010 | Gonadotrophin-releasing hormone antagonists for assisted reproductive technology | 1 |
| Hsieh 2008 | Gonadotrophin-releasing hormone antagonists for assisted reproductive technology | 1 |
| Huirne 2006 | Gonadotrophin-releasing hormone antagonists for assisted reproductive technology | 1 |
| Hwang 2004 | Gonadotrophin-releasing hormone antagonists for assisted reproductive technology | 1 |
| Inza 2004 | Gonadotrophin-releasing hormone antagonists for assisted reproductive technology | 1 |
| Karimzadeh 2010 | Gonadotrophin-releasing hormone antagonists for assisted reproductive technology | 1 |
| Khalaf 2010 | Gonadotrophin-releasing hormone antagonists for assisted reproductive technology | 1 |
| Kim 2004 | Gonadotrophin-releasing hormone antagonists for assisted reproductive technology | 1 |
| Kim 2011 | Gonadotrophin-releasing hormone antagonists for assisted reproductive technology | 1 |
| Kim 2012 | Gonadotrophin-releasing hormone antagonists for assisted reproductive technology | 1 |
| Kurzawa 2008 | Gonadotrophin-releasing hormone antagonists for assisted reproductive technology | 1 |
| Kyono 2005 | Gonadotrophin-releasing hormone antagonists for assisted reproductive technology | 1 |
| Lainas 2007 | Gonadotrophin-releasing hormone antagonists for assisted reproductive technology | 1 |
| Lainas 2010 | Gonadotrophin-releasing hormone antagonists for assisted reproductive technology | 1 |
| Lavorato 2012 | Gonadotrophin-releasing hormone antagonists for assisted reproductive technology | 1 |
| Lee 2005 | Gonadotrophin-releasing hormone antagonists for assisted reproductive technology | 1 |
| Lin 2006 | Gonadotrophin-releasing hormone antagonists for assisted reproductive technology | 1 |
| Loutradis 2004 | Gonadotrophin-releasing hormone antagonists for assisted reproductive technology | 1 |
| Marci 2005 | Gonadotrophin-releasing hormone antagonists for assisted reproductive technology | 1 |
| Martinez 2008 | Gonadotrophin-releasing hormone antagonists for assisted reproductive technology | 1 |
| Mohamed 2006 | Gonadotrophin-releasing hormone antagonists for assisted reproductive technology | 1 |
| Moraloglu 2008 | Gonadotrophin-releasing hormone antagonists for assisted reproductive technology | 1 |
| Moshin 2007 | Gonadotrophin-releasing hormone antagonists for assisted reproductive technology | 1 |
| Olivennes 2000 | Gonadotrophin-releasing hormone antagonists for assisted reproductive technology | 1 |
| Papanikolaou 2012 | Gonadotrophin-releasing hormone antagonists for assisted reproductive technology | 1 |
| Prapas 2013 | Gonadotrophin-releasing hormone antagonists for assisted reproductive technology | 1 |
| Qiao 2012 | Gonadotrophin-releasing hormone antagonists for assisted reproductive technology | 1 |
| Rabati 2012 | Gonadotrophin-releasing hormone antagonists for assisted reproductive technology | 1 |
| Revelli 2014 | Gonadotrophin-releasing hormone antagonists for assisted reproductive technology | 1 |
| Rinaldi 2014 | Gonadotrophin-releasing hormone antagonists for assisted reproductive technology | 1 |
| Rombauts 2006 | Gonadotrophin-releasing hormone antagonists for assisted reproductive technology | 1 |
| Sauer 2004 | Gonadotrophin-releasing hormone antagonists for assisted reproductive technology | 1 |
| Sbracia 2009 | Gonadotrophin-releasing hormone antagonists for assisted reproductive technology | 1 |
| Serafini 2008 | Gonadotrophin-releasing hormone antagonists for assisted reproductive technology | 1 |
| Stenbaek 2015 | Gonadotrophin-releasing hormone antagonists for assisted reproductive technology | 1 |
| Sunkara 2014 | Gonadotrophin-releasing hormone antagonists for assisted reproductive technology | 1 |
| Tazegul 2008 | Gonadotrophin-releasing hormone antagonists for assisted reproductive technology | 1 |
| Tehraninejad 2010 | Gonadotrophin-releasing hormone antagonists for assisted reproductive technology | 1 |
| Tehraninejad 2011 | Gonadotrophin-releasing hormone antagonists for assisted reproductive technology | 1 |
| Toltager 2015 | Gonadotrophin-releasing hormone antagonists for assisted reproductive technology | 1 |
| Xavier 2005 | Gonadotrophin-releasing hormone antagonists for assisted reproductive technology | 1 |
| Ye 2009 | Gonadotrophin-releasing hormone antagonists for assisted reproductive technology | 1 |
| Akerlund 1989 | Nonsteroidal anti-inflammatory drugs for dysmenorrhoea | 1 |
| Akinluyi 1987 | Nonsteroidal anti-inflammatory drugs for dysmenorrhoea | 1 |
| al-Waili 1990 | Nonsteroidal anti-inflammatory drugs for dysmenorrhoea | 1 |
| Andersch 1989 | Nonsteroidal anti-inflammatory drugs for dysmenorrhoea | 1 |
| Arnold 1983 | Nonsteroidal anti-inflammatory drugs for dysmenorrhoea | 1 |
| Balsamo 1986 | Nonsteroidal anti-inflammatory drugs for dysmenorrhoea | 1 |
| Benassi 1993 | Nonsteroidal anti-inflammatory drugs for dysmenorrhoea | 1 |
| Bitner 2004 | Nonsteroidal anti-inflammatory drugs for dysmenorrhoea | 1 |
| Budoff 1979 | Nonsteroidal anti-inflammatory drugs for dysmenorrhoea | 1 |
| Cash 1982 | Nonsteroidal anti-inflammatory drugs for dysmenorrhoea | 1 |
| Chan 1983 | Nonsteroidal anti-inflammatory drugs for dysmenorrhoea | 1 |
| Chantler 2008 | Nonsteroidal anti-inflammatory drugs for dysmenorrhoea | 1 |
| Chantler 2009 | Nonsteroidal anti-inflammatory drugs for dysmenorrhoea | 1 |
| Costa 1987a 0000 | Nonsteroidal anti-inflammatory drugs for dysmenorrhoea | 1 |
| Costa 1987b 0000 | Nonsteroidal anti-inflammatory drugs for dysmenorrhoea | 1 |
| Dandenell 1979 | Nonsteroidal anti-inflammatory drugs for dysmenorrhoea | 1 |
| Daniels 2002 | Nonsteroidal anti-inflammatory drugs for dysmenorrhoea | 1 |
| Daniels 2008 | Nonsteroidal anti-inflammatory drugs for dysmenorrhoea | 1 |
| Daniels 2009a 0000 | Nonsteroidal anti-inflammatory drugs for dysmenorrhoea | 1 |
| Daniels 2009b 0000 | Nonsteroidal anti-inflammatory drugs for dysmenorrhoea | 1 |
| Dawood 1999a 0000 | Nonsteroidal anti-inflammatory drugs for dysmenorrhoea | 1 |
| Dawood 1999b 0000 | Nonsteroidal anti-inflammatory drugs for dysmenorrhoea | 1 |
| Dawood 2007 | Nonsteroidal anti-inflammatory drugs for dysmenorrhoea | 1 |
| de Mello 2004 | Nonsteroidal anti-inflammatory drugs for dysmenorrhoea | 1 |
| De Souza 1991 | Nonsteroidal anti-inflammatory drugs for dysmenorrhoea | 1 |
| Delgado 1994 | Nonsteroidal anti-inflammatory drugs for dysmenorrhoea | 1 |
| Di Girolamo 1999 | Nonsteroidal anti-inflammatory drugs for dysmenorrhoea | 1 |
| Elder 1979 | Nonsteroidal anti-inflammatory drugs for dysmenorrhoea | 1 |
| Ezcurdia 1998 | Nonsteroidal anti-inflammatory drugs for dysmenorrhoea | 1 |
| Facchinetti 2001 | Nonsteroidal anti-inflammatory drugs for dysmenorrhoea | 1 |
| Fedele 1989 | Nonsteroidal anti-inflammatory drugs for dysmenorrhoea | 1 |
| Gleeson 1983 | Nonsteroidal anti-inflammatory drugs for dysmenorrhoea | 1 |
| Hamann 1980 | Nonsteroidal anti-inflammatory drugs for dysmenorrhoea | 1 |
| Hanson 1978 | Nonsteroidal anti-inflammatory drugs for dysmenorrhoea | 1 |
| Heidarifar 2014 | Nonsteroidal anti-inflammatory drugs for dysmenorrhoea | 1 |
| Henzl 1977b 0000 | Nonsteroidal anti-inflammatory drugs for dysmenorrhoea | 1 |
| Iacovides 2014 | Nonsteroidal anti-inflammatory drugs for dysmenorrhoea | 1 |
| Ingemanson 1984 | Nonsteroidal anti-inflammatory drugs for dysmenorrhoea | 1 |
| Jacobson 1979 | Nonsteroidal anti-inflammatory drugs for dysmenorrhoea | 1 |
| Jacobson 1983 | Nonsteroidal anti-inflammatory drugs for dysmenorrhoea | 1 |
| Kajanoja 1978 | Nonsteroidal anti-inflammatory drugs for dysmenorrhoea | 1 |
| Kajanoja 1984 | Nonsteroidal anti-inflammatory drugs for dysmenorrhoea | 1 |
| Kapadia 1978 | Nonsteroidal anti-inflammatory drugs for dysmenorrhoea | 1 |
| Kintigh 1995 | Nonsteroidal anti-inflammatory drugs for dysmenorrhoea | 1 |
| Layes Molla 1974 | Nonsteroidal anti-inflammatory drugs for dysmenorrhoea | 1 |
| Legris 1997 | Nonsteroidal anti-inflammatory drugs for dysmenorrhoea | 1 |
| Letzel 2006 | Nonsteroidal anti-inflammatory drugs for dysmenorrhoea | 1 |
| Lopez Rosales 1989 | Nonsteroidal anti-inflammatory drugs for dysmenorrhoea | 1 |
| Malmstrom 2003 | Nonsteroidal anti-inflammatory drugs for dysmenorrhoea | 1 |
| Marchini 1995 | Nonsteroidal anti-inflammatory drugs for dysmenorrhoea | 1 |
| Mehlisch 1990 | Nonsteroidal anti-inflammatory drugs for dysmenorrhoea | 1 |
| Mehlisch 1997 | Nonsteroidal anti-inflammatory drugs for dysmenorrhoea | 1 |
| Mehlisch 2003 | Nonsteroidal anti-inflammatory drugs for dysmenorrhoea | 1 |
| Milsom 1985 | Nonsteroidal anti-inflammatory drugs for dysmenorrhoea | 1 |
| Milsom 2002d 0000 | Nonsteroidal anti-inflammatory drugs for dysmenorrhoea | 1 |
| Milsom 2002e 0000 | Nonsteroidal anti-inflammatory drugs for dysmenorrhoea | 1 |
| Moggian 1986 | Nonsteroidal anti-inflammatory drugs for dysmenorrhoea | 1 |
| Morrison 1979 | Nonsteroidal anti-inflammatory drugs for dysmenorrhoea | 1 |
| Morrison 1980 | Nonsteroidal anti-inflammatory drugs for dysmenorrhoea | 1 |
| Morrison 1999 | Nonsteroidal anti-inflammatory drugs for dysmenorrhoea | 1 |
| Nahid 2009 | Nonsteroidal anti-inflammatory drugs for dysmenorrhoea | 1 |
| Onatra 1994 | Nonsteroidal anti-inflammatory drugs for dysmenorrhoea | 1 |
| Osathanondh 1985 | Nonsteroidal anti-inflammatory drugs for dysmenorrhoea | 1 |
| Osinusi 1986 | Nonsteroidal anti-inflammatory drugs for dysmenorrhoea | 1 |
| Pasquale 1988 | Nonsteroidal anti-inflammatory drugs for dysmenorrhoea | 1 |
| Pauls 1978 | Nonsteroidal anti-inflammatory drugs for dysmenorrhoea | 1 |
| Pedron 1995 | Nonsteroidal anti-inflammatory drugs for dysmenorrhoea | 1 |
| Powell 1981 | Nonsteroidal anti-inflammatory drugs for dysmenorrhoea | 1 |
| Pulkkinen 1987 | Nonsteroidal anti-inflammatory drugs for dysmenorrhoea | 1 |
| Riihiluoma 1981 | Nonsteroidal anti-inflammatory drugs for dysmenorrhoea | 1 |
| Rondel 1984 | Nonsteroidal anti-inflammatory drugs for dysmenorrhoea | 1 |
| Salmalian 2014 | Nonsteroidal anti-inflammatory drugs for dysmenorrhoea | 1 |
| Saltveit 1985 | Nonsteroidal anti-inflammatory drugs for dysmenorrhoea | 1 |
| Saltveit 1989 | Nonsteroidal anti-inflammatory drugs for dysmenorrhoea | 1 |
| Sande 1978 | Nonsteroidal anti-inflammatory drugs for dysmenorrhoea | 1 |
| Soares 1993 | Nonsteroidal anti-inflammatory drugs for dysmenorrhoea | 1 |
| Villasenor 1984 | Nonsteroidal anti-inflammatory drugs for dysmenorrhoea | 1 |
| Wilhelmsson 1985a 0000 | Nonsteroidal anti-inflammatory drugs for dysmenorrhoea | 1 |
| Wilhelmsson 1985b 0000 | Nonsteroidal anti-inflammatory drugs for dysmenorrhoea | 1 |
| Yu 2014 | Nonsteroidal anti-inflammatory drugs for dysmenorrhoea | 1 |
| Agarwal 2004 | Intra-uterine insemination for unexplained subfertility | 1 |
| Arcaini 1996 | Intra-uterine insemination for unexplained subfertility | 1 |
| Arici 1994 | Intra-uterine insemination for unexplained subfertility | 1 |
| Bhattacharya 2008 | Intra-uterine insemination for unexplained subfertility | 1 |
| Chung 1995 | Intra-uterine insemination for unexplained subfertility | 1 |
| Crosignani 1991 | Intra-uterine insemination for unexplained subfertility | 1 |
| Deaton 1990 | Intra-uterine insemination for unexplained subfertility | 1 |
| Goverde 2000 | Intra-uterine insemination for unexplained subfertility | 1 |
| Guzick 1999 | Intra-uterine insemination for unexplained subfertility | 1 |
| Janko 1998 | Intra-uterine insemination for unexplained subfertility | 1 |
| Karlstrom 1993 | Intra-uterine insemination for unexplained subfertility | 1 |
| Melis 1995 | Intra-uterine insemination for unexplained subfertility | 1 |
| Murdoch 1991 | Intra-uterine insemination for unexplained subfertility | 1 |
| Steures 2006a 0000 | Intra-uterine insemination for unexplained subfertility | 1 |
| Barnet 2009 | Education for contraceptive use by women after childbirth | 1 |
| Bashour 2008 | Education for contraceptive use by women after childbirth | 1 |
| Black 2006 | Education for contraceptive use by women after childbirth | 1 |
| Bolam 1998 | Education for contraceptive use by women after childbirth | 1 |
| Gilliam 2004 | Education for contraceptive use by women after childbirth | 1 |
| Katz 2011 | Education for contraceptive use by women after childbirth | 1 |
| O'Sullivan 1992 | Education for contraceptive use by women after childbirth | 1 |
| Quinlivan 2003 | Education for contraceptive use by women after childbirth | 1 |
| Saeed 2008 | Education for contraceptive use by women after childbirth | 1 |
| Simmons 2013 | Education for contraceptive use by women after childbirth | 1 |
| Tang 2014 | Education for contraceptive use by women after childbirth | 1 |
| Torres 2014 | Education for contraceptive use by women after childbirth | 1 |
| Bergqvist 2005 | Ketogenic diet and other dietary treatments for epilepsy | 1 |
| Kossoff 2007 | Ketogenic diet and other dietary treatments for epilepsy | 1 |
| Neal 2008 | Ketogenic diet and other dietary treatments for epilepsy | 1 |
| Seo 2007 | Ketogenic diet and other dietary treatments for epilepsy | 1 |
| El-Rashidy 2013 | Ketogenic diet and other dietary treatments for epilepsy | 1 |
| Raju 2011 | Ketogenic diet and other dietary treatments for epilepsy | 1 |
| Sharma 2013 | Ketogenic diet and other dietary treatments for epilepsy | 1 |
| Baulac 2010 | Lamotrigine add-on for drug-resistant partial epilepsy | 1 |
| Binnie 1989 | Lamotrigine add-on for drug-resistant partial epilepsy | 1 |
| Boas 1996 | Lamotrigine add-on for drug-resistant partial epilepsy | 1 |
| Duchowny 1999 | Lamotrigine add-on for drug-resistant partial epilepsy | 1 |
| Jawad 1989 | Lamotrigine add-on for drug-resistant partial epilepsy | 1 |
| Loiseau 1990 | Lamotrigine add-on for drug-resistant partial epilepsy | 1 |
| Matsuo 1993 | Lamotrigine add-on for drug-resistant partial epilepsy | 1 |
| Messenheimer 1994 | Lamotrigine add-on for drug-resistant partial epilepsy | 1 |
| Naritoku 2007 | Lamotrigine add-on for drug-resistant partial epilepsy | 1 |
| Piña-Garza 2008 | Lamotrigine add-on for drug-resistant partial epilepsy | 1 |
| Schachter 1995 | Lamotrigine add-on for drug-resistant partial epilepsy | 1 |
| Schapel 1993 | Lamotrigine add-on for drug-resistant partial epilepsy | 1 |
| Schmidt 1993 | Lamotrigine add-on for drug-resistant partial epilepsy | 1 |
| Smith 1993 | Lamotrigine add-on for drug-resistant partial epilepsy | 1 |
| Wolter 1997 | Home versus hospital intravenous antibiotic therapy for cystic fibrosis | 1 |
| Calvey 1985 | Branched-chain amino acids for people with hepatic encephalopathy | 1 |
| Cerra 1985 | Branched-chain amino acids for people with hepatic encephalopathy | 1 |
| Egberts 1985 | Branched-chain amino acids for people with hepatic encephalopathy | 1 |
| Fiaccadori 1984 | Branched-chain amino acids for people with hepatic encephalopathy | 1 |
| Hayashi 1991 | Branched-chain amino acids for people with hepatic encephalopathy | 1 |
| Horst 1984 | Branched-chain amino acids for people with hepatic encephalopathy | 1 |
| Hwang 1988 | Branched-chain amino acids for people with hepatic encephalopathy | 1 |
| Les 2011 | Branched-chain amino acids for people with hepatic encephalopathy | 1 |
| Marchesini 1990 | Branched-chain amino acids for people with hepatic encephalopathy | 1 |
| Marchesini 2003 | Branched-chain amino acids for people with hepatic encephalopathy | 1 |
| Michel 1985 | Branched-chain amino acids for people with hepatic encephalopathy | 1 |
| Muto 2005 | Branched-chain amino acids for people with hepatic encephalopathy | 1 |
| Plauth 1993 | Branched-chain amino acids for people with hepatic encephalopathy | 1 |
| Rossi-Fanelli 1986 | Branched-chain amino acids for people with hepatic encephalopathy | 1 |
| Strauss 1986 | Branched-chain amino acids for people with hepatic encephalopathy | 1 |
| Vilstrup 1990 | Branched-chain amino acids for people with hepatic encephalopathy | 1 |
| Batey 1987 | Alpha2-adrenergic agonists for the management of opioid withdrawal | 2 |
| Bearn 1996 | Alpha2-adrenergic agonists for the management of opioid withdrawal | 2 |
| Benos 1985 | Alpha2-adrenergic agonists for the management of opioid withdrawal | 2 |
| Bertschy 1997 | Alpha2-adrenergic agonists for the management of opioid withdrawal | 2 |
| Bruno 1979 | Alpha2-adrenergic agonists for the management of opioid withdrawal | 2 |
| Cami 1985 | Alpha2-adrenergic agonists for the management of opioid withdrawal | 2 |
| Carnwath 1998 | Alpha2-adrenergic agonists for the management of opioid withdrawal | 2 |
| Gerra 1995 | Alpha2-adrenergic agonists for the management of opioid withdrawal | 2 |
| Gerra 2000 | Alpha2-adrenergic agonists for the management of opioid withdrawal | 2 |
| Gupta 1988 | Alpha2-adrenergic agonists for the management of opioid withdrawal | 2 |
| Howells 2002 | Alpha2-adrenergic agonists for the management of opioid withdrawal | 2 |
| Jiang 1993 | Alpha2-adrenergic agonists for the management of opioid withdrawal | 2 |
| Kahn 1997 | Alpha2-adrenergic agonists for the management of opioid withdrawal | 2 |
| Kleber 1985 | Alpha2-adrenergic agonists for the management of opioid withdrawal | 2 |
| Li 2002 | Alpha2-adrenergic agonists for the management of opioid withdrawal | 2 |
| Lin 1997 | Alpha2-adrenergic agonists for the management of opioid withdrawal | 2 |
| Muga 1990 | Alpha2-adrenergic agonists for the management of opioid withdrawal | 2 |
| Nazari 2013 | Alpha2-adrenergic agonists for the management of opioid withdrawal | 2 |
| San 1990 | Alpha2-adrenergic agonists for the management of opioid withdrawal | 2 |
| San 1994 | Alpha2-adrenergic agonists for the management of opioid withdrawal | 2 |
| Senay 1983 | Alpha2-adrenergic agonists for the management of opioid withdrawal | 2 |
| Sos 2000 | Alpha2-adrenergic agonists for the management of opioid withdrawal | 2 |
| Umbricht 2003 | Alpha2-adrenergic agonists for the management of opioid withdrawal | 2 |
| Vilalta 1987 | Alpha2-adrenergic agonists for the management of opioid withdrawal | 2 |
| Washton 1981 | Alpha2-adrenergic agonists for the management of opioid withdrawal | 2 |
| Yu 2008 | Alpha2-adrenergic agonists for the management of opioid withdrawal | 2 |
| Ahring 1992 | Interactive telemedicine: effects on professional practice and health care outcomes | 2 |
| Al Khatib 2009 | Interactive telemedicine: effects on professional practice and health care outcomes | 2 |
| Antonicelli 2008 | Interactive telemedicine: effects on professional practice and health care outcomes | 2 |
| Artinian 2007 | Interactive telemedicine: effects on professional practice and health care outcomes | 1 |
| Benatar 2003 | Interactive telemedicine: effects on professional practice and health care outcomes | 2 |
| Bergmo 2009 | Interactive telemedicine: effects on professional practice and health care outcomes | 2 |
| Biermann 2000 | Interactive telemedicine: effects on professional practice and health care outcomes | 2 |
| Boaz 2009 | Interactive telemedicine: effects on professional practice and health care outcomes | 2 |
| Bond 2007 | Interactive telemedicine: effects on professional practice and health care outcomes | 2 |
| Bowles 2011 | Interactive telemedicine: effects on professional practice and health care outcomes | 2 |
| Boyne 2012 | Interactive telemedicine: effects on professional practice and health care outcomes | 2 |
| Capomolla 2004 | Interactive telemedicine: effects on professional practice and health care outcomes | 1 |
| Chambers 2006 | Interactive telemedicine: effects on professional practice and health care outcomes | 2 |
| Chan 2007 | Interactive telemedicine: effects on professional practice and health care outcomes | 2 |
| Charpentier 2011 | Interactive telemedicine: effects on professional practice and health care outcomes | 2 |
| Chase 2003 | Interactive telemedicine: effects on professional practice and health care outcomes | 2 |
| Chaudry 2010 | Interactive telemedicine: effects on professional practice and health care outcomes | 1 |
| Chiantera 2005 | Interactive telemedicine: effects on professional practice and health care outcomes | 1 |
| Chong 2012 | Interactive telemedicine: effects on professional practice and health care outcomes | 2 |
| Cleland 2005 | Interactive telemedicine: effects on professional practice and health care outcomes | 1 |
| Cross 2012 | Interactive telemedicine: effects on professional practice and health care outcomes | 2 |
| Crossley 2011 | Interactive telemedicine: effects on professional practice and health care outcomes | 1 |
| Dallollio 2008 | Interactive telemedicine: effects on professional practice and health care outcomes | 2 |
| Dansky 2008 | Interactive telemedicine: effects on professional practice and health care outcomes | 2 |
| Dar 2009 | Interactive telemedicine: effects on professional practice and health care outcomes | 2 |
| Davis 2010 | Interactive telemedicine: effects on professional practice and health care outcomes | 1 |
| De Las Cuevas 2006 | Interactive telemedicine: effects on professional practice and health care outcomes | 2 |
| Dendale 2012 | Interactive telemedicine: effects on professional practice and health care outcomes | 1 |
| Ellison 2004 | Interactive telemedicine: effects on professional practice and health care outcomes | 2 |
| Ellison 2007 | Interactive telemedicine: effects on professional practice and health care outcomes | 2 |
| Finkelstein 2006 | Interactive telemedicine: effects on professional practice and health care outcomes | 2 |
| Giordano 2009 | Interactive telemedicine: effects on professional practice and health care outcomes | 1 |
| Goldberg 2003 | Interactive telemedicine: effects on professional practice and health care outcomes | 2 |
| Gray 2000 | Interactive telemedicine: effects on professional practice and health care outcomes | 2 |
| Halimi 2008 | Interactive telemedicine: effects on professional practice and health care outcomes | 2 |
| Harrison 1999 | Interactive telemedicine: effects on professional practice and health care outcomes | 2 |
| Hermens 2007 | Interactive telemedicine: effects on professional practice and health care outcomes | 1 |
| Hopp 2006 | Interactive telemedicine: effects on professional practice and health care outcomes | 2 |
| Hui 2006 | Interactive telemedicine: effects on professional practice and health care outcomes | 2 |
| Izquierdo 2003 | Interactive telemedicine: effects on professional practice and health care outcomes | 2 |
| Izquierdo 2009 | Interactive telemedicine: effects on professional practice and health care outcomes | 2 |
| 39083 | Interactive telemedicine: effects on professional practice and health care outcomes | 2 |
| Jansa 2006 | Interactive telemedicine: effects on professional practice and health care outcomes | 2 |
| Jerant 2001 | Interactive telemedicine: effects on professional practice and health care outcomes | 2 |
| Kashem 2008 | Interactive telemedicine: effects on professional practice and health care outcomes | 2 |
| Kearney 2009 | Interactive telemedicine: effects on professional practice and health care outcomes | 2 |
| Kim 2007 | Interactive telemedicine: effects on professional practice and health care outcomes | 1 |
| King 2009 | Interactive telemedicine: effects on professional practice and health care outcomes | 1 |
| Koehler 2011 | Interactive telemedicine: effects on professional practice and health care outcomes | 2 |
| Koff 2009 | Interactive telemedicine: effects on professional practice and health care outcomes | 2 |
| Kwon 2003 | Interactive telemedicine: effects on professional practice and health care outcomes | 1 |
| Lewis 2010 | Interactive telemedicine: effects on professional practice and health care outcomes | 1 |
| Madigan 2013 | Interactive telemedicine: effects on professional practice and health care outcomes | 2 |
| Madsen 2008 | Interactive telemedicine: effects on professional practice and health care outcomes | 2 |
| Marrero 1995 | Interactive telemedicine: effects on professional practice and health care outcomes | 2 |
| McCarrier 2009 | Interactive telemedicine: effects on professional practice and health care outcomes | 2 |
| McCrossan 2012 | Interactive telemedicine: effects on professional practice and health care outcomes | 1 |
| McMahon 2005 | Interactive telemedicine: effects on professional practice and health care outcomes | 1 |
| Meyer 2008 | Interactive telemedicine: effects on professional practice and health care outcomes | 1 |
| Mitchell 2008 | Interactive telemedicine: effects on professional practice and health care outcomes | 2 |
| Morland 2010 | Interactive telemedicine: effects on professional practice and health care outcomes | 2 |
| Mortara 2009 | Interactive telemedicine: effects on professional practice and health care outcomes | 1 |
| Nguyen 2008 | Interactive telemedicine: effects on professional practice and health care outcomes | 2 |
| Noel 2004 | Interactive telemedicine: effects on professional practice and health care outcomes | 2 |
| Oakley 2000 | Interactive telemedicine: effects on professional practice and health care outcomes | 2 |
| Parati 2009 | Interactive telemedicine: effects on professional practice and health care outcomes | 2 |
| Piron 2009 | Interactive telemedicine: effects on professional practice and health care outcomes | 2 |
| Poon 2005 | Interactive telemedicine: effects on professional practice and health care outcomes | 2 |
| Ralston 2009 | Interactive telemedicine: effects on professional practice and health care outcomes | 1 |
| Rasmussen 2005 | Interactive telemedicine: effects on professional practice and health care outcomes | 2 |
| Rodriguez-Idigoras 2009 | Interactive telemedicine: effects on professional practice and health care outcomes | 1 |
| Rogers 2001 | Interactive telemedicine: effects on professional practice and health care outcomes | 1 |
| Ruskin 2004 | Interactive telemedicine: effects on professional practice and health care outcomes | 2 |
| Scherr 2009 | Interactive telemedicine: effects on professional practice and health care outcomes | 2 |
| Schwarz 2008 | Interactive telemedicine: effects on professional practice and health care outcomes | 2 |
| Seto 2012 | Interactive telemedicine: effects on professional practice and health care outcomes | 2 |
| Shea 2006 | Interactive telemedicine: effects on professional practice and health care outcomes | 2 |
| Soran 2008 | Interactive telemedicine: effects on professional practice and health care outcomes | 1 |
| Spaeder 2006 | Interactive telemedicine: effects on professional practice and health care outcomes | 1 |
| Stone 2010 | Interactive telemedicine: effects on professional practice and health care outcomes | 1 |
| Taylor 2006 | Interactive telemedicine: effects on professional practice and health care outcomes | 1 |
| Thompson 2009 | Interactive telemedicine: effects on professional practice and health care outcomes | 2 |
| Van der Meer 2010 | Interactive telemedicine: effects on professional practice and health care outcomes | 2 |
| Wakefield 2008 | Interactive telemedicine: effects on professional practice and health care outcomes | 2 |
| Waldmann 2008 | Interactive telemedicine: effects on professional practice and health care outcomes | 1 |
| Wallace 2002 | Interactive telemedicine: effects on professional practice and health care outcomes | 2 |
| Weintraub 2010 | Interactive telemedicine: effects on professional practice and health care outcomes | 2 |
| Whitlock 2000 | Interactive telemedicine: effects on professional practice and health care outcomes | 2 |
| Willems 2008 | Interactive telemedicine: effects on professional practice and health care outcomes | 2 |
| Wojcicki 2001 | Interactive telemedicine: effects on professional practice and health care outcomes | 1 |
| Wong 2006 | Interactive telemedicine: effects on professional practice and health care outcomes | 2 |
| Woodend 2008 | Interactive telemedicine: effects on professional practice and health care outcomes | 2 |
| Wootton 2000 | Interactive telemedicine: effects on professional practice and health care outcomes | 1 |
| Agarwala 1968 | Tricyclic and related drugs for nocturnal enuresis in children | 3 |
| Alderton 1967 | Tricyclic and related drugs for nocturnal enuresis in children | 3 |
| Alderton 1970 | Tricyclic and related drugs for nocturnal enuresis in children | 3 |
| Attenburrow 1984 | Tricyclic and related drugs for nocturnal enuresis in children | 3 |
| Banerjee 1993 | Tricyclic and related drugs for nocturnal enuresis in children | 3 |
| Batislam 1995 | Tricyclic and related drugs for nocturnal enuresis in children | 3 |
| Bhatia 1990 | Tricyclic and related drugs for nocturnal enuresis in children | 3 |
| Bindelglas 1968 | Tricyclic and related drugs for nocturnal enuresis in children | 3 |
| Burke 1995 | Tricyclic and related drugs for nocturnal enuresis in children | 3 |
| Ciotti 1983 | Tricyclic and related drugs for nocturnal enuresis in children | 3 |
| Danquah 1975 | Tricyclic and related drugs for nocturnal enuresis in children | 3 |
| Drew 1966 | Tricyclic and related drugs for nocturnal enuresis in children | 3 |
| Esmaeili 2008 | Tricyclic and related drugs for nocturnal enuresis in children | 3 |
| Forsythe 1969 | Tricyclic and related drugs for nocturnal enuresis in children | 3 |
| Forsythe 1972a 0000 | Tricyclic and related drugs for nocturnal enuresis in children | 3 |
| Forsythe 1972b 0000 | Tricyclic and related drugs for nocturnal enuresis in children | 3 |
| Fournier 1987 | Tricyclic and related drugs for nocturnal enuresis in children | 3 |
| Friday 1966 | Tricyclic and related drugs for nocturnal enuresis in children | 3 |
| Haegglund 1964 | Tricyclic and related drugs for nocturnal enuresis in children | 3 |
| Harrison 1970 | Tricyclic and related drugs for nocturnal enuresis in children | 3 |
| Hoashi 1995 | Tricyclic and related drugs for nocturnal enuresis in children | 3 |
| Hodes 1973 | Tricyclic and related drugs for nocturnal enuresis in children | 3 |
| Holt 1986 | Tricyclic and related drugs for nocturnal enuresis in children | 3 |
| Iester 1991 | Tricyclic and related drugs for nocturnal enuresis in children | 3 |
| Ingle 1968 | Tricyclic and related drugs for nocturnal enuresis in children | 3 |
| Kang 2003 | Tricyclic and related drugs for nocturnal enuresis in children | 3 |
| Khorana 1972 | Tricyclic and related drugs for nocturnal enuresis in children | 3 |
| Kolvin 1972 | Tricyclic and related drugs for nocturnal enuresis in children | 3 |
| Kumazawa 1990 | Tricyclic and related drugs for nocturnal enuresis in children | 3 |
| Kunin 1970 | Tricyclic and related drugs for nocturnal enuresis in children | 3 |
| Lake 1968 | Tricyclic and related drugs for nocturnal enuresis in children | 3 |
| Lee 2005 | Tricyclic and related drugs for nocturnal enuresis in children | 3 |
| Liederman 1969 | Tricyclic and related drugs for nocturnal enuresis in children | 3 |
| Lines 1968 | Tricyclic and related drugs for nocturnal enuresis in children | 3 |
| Manhas 1967 | Tricyclic and related drugs for nocturnal enuresis in children | 3 |
| Martin 1971 | Tricyclic and related drugs for nocturnal enuresis in children | 3 |
| Maxwell 1971 | Tricyclic and related drugs for nocturnal enuresis in children | 3 |
| McKendry 1975 | Tricyclic and related drugs for nocturnal enuresis in children | 3 |
| Mehrotra 1980 | Tricyclic and related drugs for nocturnal enuresis in children | 3 |
| Miyazaki 1973 | Tricyclic and related drugs for nocturnal enuresis in children | 3 |
| Moltke 1979 | Tricyclic and related drugs for nocturnal enuresis in children | 3 |
| Motavalli 1994 | Tricyclic and related drugs for nocturnal enuresis in children | 3 |
| Naitoh 2005 | Tricyclic and related drugs for nocturnal enuresis in children | 3 |
| Netley 1984 | Tricyclic and related drugs for nocturnal enuresis in children | 3 |
| Neveus 2008 | Tricyclic and related drugs for nocturnal enuresis in children | 3 |
| Petersen 1974 | Tricyclic and related drugs for nocturnal enuresis in children | 3 |
| Poussaint 1965a 0000 | Tricyclic and related drugs for nocturnal enuresis in children | 3 |
| Poussaint 1966a 0000 | Tricyclic and related drugs for nocturnal enuresis in children | 3 |
| Poussaint 1966b 0000 | Tricyclic and related drugs for nocturnal enuresis in children | 3 |
| Roy 1970 | Tricyclic and related drugs for nocturnal enuresis in children | 3 |
| Scholander 1968 | Tricyclic and related drugs for nocturnal enuresis in children | 3 |
| Schröder 1971 | Tricyclic and related drugs for nocturnal enuresis in children | 3 |
| Seo 2001 | Tricyclic and related drugs for nocturnal enuresis in children | 3 |
| Shaffer 1968 | Tricyclic and related drugs for nocturnal enuresis in children | 3 |
| Shah 1971 | Tricyclic and related drugs for nocturnal enuresis in children | 3 |
| Smellie 1996 | Tricyclic and related drugs for nocturnal enuresis in children | 3 |
| Tahmaz 2000 | Tricyclic and related drugs for nocturnal enuresis in children | 3 |
| Thomsen 1967 | Tricyclic and related drugs for nocturnal enuresis in children | 3 |
| Treffert 1964 | Tricyclic and related drugs for nocturnal enuresis in children | 3 |
| Vertucci 1997 | Tricyclic and related drugs for nocturnal enuresis in children | 3 |
| Wagner 1982 | Tricyclic and related drugs for nocturnal enuresis in children | 3 |
| Ye 2001 | Tricyclic and related drugs for nocturnal enuresis in children | 3 |
| Yurdakok 1986 | Tricyclic and related drugs for nocturnal enuresis in children | 3 |
| Yurdakok 1987 | Tricyclic and related drugs for nocturnal enuresis in children | 3 |
| Aziminekoo 2015 | Cleavage stage versus blastocyst stage embryo transfer in assisted reproductive technology | 1 |
| Brugnon 2010 | Cleavage stage versus blastocyst stage embryo transfer in assisted reproductive technology | 1 |
| Bungum 2003 | Cleavage stage versus blastocyst stage embryo transfer in assisted reproductive technology | 1 |
| Coskun 2000 | Cleavage stage versus blastocyst stage embryo transfer in assisted reproductive technology | 1 |
| Devreker 2000 | Cleavage stage versus blastocyst stage embryo transfer in assisted reproductive technology | 1 |
| Elgindy 2011 | Cleavage stage versus blastocyst stage embryo transfer in assisted reproductive technology | 1 |
| Emiliani 2003 | Cleavage stage versus blastocyst stage embryo transfer in assisted reproductive technology | 1 |
| Fernandez-Shaw 2015 | Cleavage stage versus blastocyst stage embryo transfer in assisted reproductive technology | 1 |
| Fisch 2007 | Cleavage stage versus blastocyst stage embryo transfer in assisted reproductive technology | 1 |
| Frattarelli 2003 | Cleavage stage versus blastocyst stage embryo transfer in assisted reproductive technology | 1 |
| Gaafar 2015 | Cleavage stage versus blastocyst stage embryo transfer in assisted reproductive technology | 1 |
| Gardner 1998 | Cleavage stage versus blastocyst stage embryo transfer in assisted reproductive technology | 1 |
| Hreinsson 2004 | Cleavage stage versus blastocyst stage embryo transfer in assisted reproductive technology | 1 |
| Karaki 2002 | Cleavage stage versus blastocyst stage embryo transfer in assisted reproductive technology | 1 |
| Kaur 2014 | Cleavage stage versus blastocyst stage embryo transfer in assisted reproductive technology | 1 |
| Kolibianakis 2004 | Cleavage stage versus blastocyst stage embryo transfer in assisted reproductive technology | 1 |
| Levitas 2004 | Cleavage stage versus blastocyst stage embryo transfer in assisted reproductive technology | 1 |
| Levron 2002 | Cleavage stage versus blastocyst stage embryo transfer in assisted reproductive technology | 1 |
| Livingstone 2002 | Cleavage stage versus blastocyst stage embryo transfer in assisted reproductive technology | 1 |
| Motta 1998 | Cleavage stage versus blastocyst stage embryo transfer in assisted reproductive technology | 1 |
| Pantos 2004 | Cleavage stage versus blastocyst stage embryo transfer in assisted reproductive technology | 1 |
| Papanikolaou 2005 | Cleavage stage versus blastocyst stage embryo transfer in assisted reproductive technology | 1 |
| Papanikolaou 2006 | Cleavage stage versus blastocyst stage embryo transfer in assisted reproductive technology | 1 |
| Rienzi 2002 | Cleavage stage versus blastocyst stage embryo transfer in assisted reproductive technology | 1 |
| Schillaci 2002 | Cleavage stage versus blastocyst stage embryo transfer in assisted reproductive technology | 1 |
| Ten 2011 | Cleavage stage versus blastocyst stage embryo transfer in assisted reproductive technology | 1 |
| Van der Auwera 2002 | Cleavage stage versus blastocyst stage embryo transfer in assisted reproductive technology | 1 |
| Abdelmaeboud 2014 | Dietary supplements for dysmenorrhoea | 1 |
| Akbari 2012 | Dietary supplements for dysmenorrhoea | 1 |
| Akhavan Amjadi 2009 | Dietary supplements for dysmenorrhoea | 1 |
| Bani 2014 | Dietary supplements for dysmenorrhoea | 1 |
| Bokaie 2013 | Dietary supplements for dysmenorrhoea | 1 |
| Dolation 2010 | Dietary supplements for dysmenorrhoea | 1 |
| Doubova 2007 | Dietary supplements for dysmenorrhoea | 1 |
| Ghodsi 2014 | Dietary supplements for dysmenorrhoea | 1 |
| Gokhale 1996 | Dietary supplements for dysmenorrhoea | 1 |
| Heidarifar 2014 | Dietary supplements for dysmenorrhoea | 1 |
| Hosseinlou 2014 | Dietary supplements for dysmenorrhoea | 1 |
| Iravani 2009 | Dietary supplements for dysmenorrhoea | 1 |
| Jenabi 2010 | Dietary supplements for dysmenorrhoea | 1 |
| Jenabi 2012 | Dietary supplements for dysmenorrhoea | 1 |
| Jenabi 2013 | Dietary supplements for dysmenorrhoea | 1 |
| Kashanian 2013 | Dietary supplements for dysmenorrhoea | 1 |
| Kashefi 2014 | Dietary supplements for dysmenorrhoea | 1 |
| Khorshidi 2003 | Dietary supplements for dysmenorrhoea | 1 |
| Modaress 2011 | Dietary supplements for dysmenorrhoea | 1 |
| Moslemi 2012 | Dietary supplements for dysmenorrhoea | 1 |
| Nasehi 2013 | Dietary supplements for dysmenorrhoea | 1 |
| Nayeban 2014 | Dietary supplements for dysmenorrhoea | 1 |
| Nazarpour 2007 | Dietary supplements for dysmenorrhoea | 1 |
| Rahnama 2010 | Dietary supplements for dysmenorrhoea | 1 |
| Rahnama 2012 | Dietary supplements for dysmenorrhoea | 1 |
| Rehman 2015 | Dietary supplements for dysmenorrhoea | 1 |
| Schwertner 2013 | Dietary supplements for dysmenorrhoea | 1 |
| Henderson 1994 | Omega-3 fatty acids for cystic fibrosis | 1 |
| Keen 2010 | Omega-3 fatty acids for cystic fibrosis | 1 |
| Lawrence 1993 | Omega-3 fatty acids for cystic fibrosis | 1 |
| Panchaud 2006 | Omega-3 fatty acids for cystic fibrosis | 1 |
| Annane 2002 | Corticosteroids for treating sepsis | 1 |
| Annane 2010 | Corticosteroids for treating sepsis | 1 |
| Arabi 2011 | Corticosteroids for treating sepsis | 1 |
| Bollaert 1998 | Corticosteroids for treating sepsis | 1 |
| Bone 1987 | Corticosteroids for treating sepsis | 1 |
| Briegel 1999 | Corticosteroids for treating sepsis | 1 |
| Chawla 1999 | Corticosteroids for treating sepsis | 1 |
| Cicarelli 2007 | Corticosteroids for treating sepsis | 1 |
| Confalonieri 2005 | Corticosteroids for treating sepsis | 1 |
| CSG 1963 | Corticosteroids for treating sepsis | 1 |
| Gordon 2014 | Corticosteroids for treating sepsis | 1 |
| Hu 2009 | Corticosteroids for treating sepsis | 1 |
| Huh 2007 | Corticosteroids for treating sepsis | 1 |
| Keh 2003 | Corticosteroids for treating sepsis | 1 |
| Liu 2012 | Corticosteroids for treating sepsis | 1 |
| Luce 1988 | Corticosteroids for treating sepsis | 1 |
| Meduri 2007 | Corticosteroids for treating sepsis | 1 |
| Meijvis 2011 | Corticosteroids for treating sepsis | 1 |
| Oppert 2005 | Corticosteroids for treating sepsis | 1 |
| Rezk 2013 | Corticosteroids for treating sepsis | 1 |
| Rinaldi 2006 | Corticosteroids for treating sepsis | 1 |
| Sabry 2011 | Corticosteroids for treating sepsis | 1 |
| Schumer 1976 | Corticosteroids for treating sepsis | 1 |
| Slusher 1996 | Corticosteroids for treating sepsis | 1 |
| Snijders 2010 | Corticosteroids for treating sepsis | 1 |
| Sprung 1984 | Corticosteroids for treating sepsis | 1 |
| Sprung 2008 | Corticosteroids for treating sepsis | 1 |
| Tandan 2005 | Corticosteroids for treating sepsis | 1 |
| Torres 2015 | Corticosteroids for treating sepsis | 1 |
| Valoor 2009 | Corticosteroids for treating sepsis | 1 |
| VASSCSG 1987 | Corticosteroids for treating sepsis | 1 |
| Yildiz 2002 | Corticosteroids for treating sepsis | 1 |
| Yildiz 2011 | Corticosteroids for treating sepsis | 1 |
| Albo 2007 | Open retropubic colposuspension for urinary incontinence in women | 1 |
| Ankardal 2001 | Open retropubic colposuspension for urinary incontinence in women | 1 |
| Athanassopoulos 1996 | Open retropubic colposuspension for urinary incontinence in women | 1 |
| Bai 2005 | Open retropubic colposuspension for urinary incontinence in women | 1 |
| Bandarian 2011 | Open retropubic colposuspension for urinary incontinence in women | 1 |
| Berglund 1996 | Open retropubic colposuspension for urinary incontinence in women | 1 |
| Bergman 1989a 0000 | Open retropubic colposuspension for urinary incontinence in women | 1 |
| Bergman 1989b 0000 | Open retropubic colposuspension for urinary incontinence in women | 1 |
| Burton 1994 | Open retropubic colposuspension for urinary incontinence in women | 1 |
| Carey 2000 | Open retropubic colposuspension for urinary incontinence in women | 1 |
| Colombo 1994 | Open retropubic colposuspension for urinary incontinence in women | 1 |
| Colombo 1996 | Open retropubic colposuspension for urinary incontinence in women | 1 |
| Colombo 2000 | Open retropubic colposuspension for urinary incontinence in women | 1 |
| Corcos 2001 | Open retropubic colposuspension for urinary incontinence in women | 1 |
| Demirci 2001 | Open retropubic colposuspension for urinary incontinence in women | 1 |
| Drahoradova 2004 | Open retropubic colposuspension for urinary incontinence in women | 1 |
| El Barky 2005 | Open retropubic colposuspension for urinary incontinence in women | 1 |
| El-Din Shawki 2012 | Open retropubic colposuspension for urinary incontinence in women | 1 |
| Elshawaf 2009 | Open retropubic colposuspension for urinary incontinence in women | 1 |
| Enzelsberger 1996 | Open retropubic colposuspension for urinary incontinence in women | 1 |
| Fatthy 2001 | Open retropubic colposuspension for urinary incontinence in women | 1 |
| Fischer 2001 | Open retropubic colposuspension for urinary incontinence in women | 1 |
| German 1994 | Open retropubic colposuspension for urinary incontinence in women | 1 |
| Gilja 1998 | Open retropubic colposuspension for urinary incontinence in women | 1 |
| Halaska 2001 | Open retropubic colposuspension for urinary incontinence in women | 1 |
| Han 2001 | Open retropubic colposuspension for urinary incontinence in women | 1 |
| Henriksson 1978 | Open retropubic colposuspension for urinary incontinence in women | 1 |
| Holmes 1985 | Open retropubic colposuspension for urinary incontinence in women | 1 |
| Kammerer-Doak 1999 | Open retropubic colposuspension for urinary incontinence in women | 1 |
| Kitchener 2006 | Open retropubic colposuspension for urinary incontinence in women | 1 |
| Klarskov 1986 | Open retropubic colposuspension for urinary incontinence in women | 1 |
| Koelbl 2002 | Open retropubic colposuspension for urinary incontinence in women | 1 |
| Liapis 1996 | Open retropubic colposuspension for urinary incontinence in women | 1 |
| Liapis 2002 | Open retropubic colposuspension for urinary incontinence in women | 1 |
| Mak 2000 | Open retropubic colposuspension for urinary incontinence in women | 1 |
| McCrery 2005 | Open retropubic colposuspension for urinary incontinence in women | 1 |
| Morris 2001 | Open retropubic colposuspension for urinary incontinence in women | 1 |
| Mundy 1983 | Open retropubic colposuspension for urinary incontinence in women | 1 |
| O'Sullivan 2000 | Open retropubic colposuspension for urinary incontinence in women | 1 |
| Osman 2003 | Open retropubic colposuspension for urinary incontinence in women | 1 |
| Palma 1985 | Open retropubic colposuspension for urinary incontinence in women | 1 |
| Quadri 1985 | Open retropubic colposuspension for urinary incontinence in women | 1 |
| Quadri 1999 | Open retropubic colposuspension for urinary incontinence in women | 1 |
| Sand 2000 | Open retropubic colposuspension for urinary incontinence in women | 1 |
| Sivaslioglu 2007 | Open retropubic colposuspension for urinary incontinence in women | 1 |
| Stangel-Wojcikiewicz 2008 | Open retropubic colposuspension for urinary incontinence in women | 1 |
| Su 1997 | Open retropubic colposuspension for urinary incontinence in women | 1 |
| Summitt 2000 | Open retropubic colposuspension for urinary incontinence in women | 1 |
| Tapp 1989 | Open retropubic colposuspension for urinary incontinence in women | 1 |
| Tellez Martinez-Fornes 2009 | Open retropubic colposuspension for urinary incontinence in women | 1 |
| Trabuco 2014 | Open retropubic colposuspension for urinary incontinence in women | 1 |
| Tuygun 2006 | Open retropubic colposuspension for urinary incontinence in women | 1 |
| Ustun 2005 | Open retropubic colposuspension for urinary incontinence in women | 1 |
| Wang 2003 | Open retropubic colposuspension for urinary incontinence in women | 1 |
| Ward 2002 | Open retropubic colposuspension for urinary incontinence in women | 1 |
| Comorosan 1993 | Electromagnetic therapy for treating pressure ulcers | 1 |
| Salzberg 1995 | Electromagnetic therapy for treating pressure ulcers | 1 |
| Aranda 1976 | Techniques for the interruption of tubal patency for female sterilisation | 1 |
| Aranda 1985 | Techniques for the interruption of tubal patency for female sterilisation | 1 |
| Argueta 1980 | Techniques for the interruption of tubal patency for female sterilisation | 1 |
| Dominik 2000 | Techniques for the interruption of tubal patency for female sterilisation | 1 |
| Geirsson 1985 | Techniques for the interruption of tubal patency for female sterilisation | 1 |
| Gentile 2006 | Techniques for the interruption of tubal patency for female sterilisation | 1 |
| Goynumer 2009 | Techniques for the interruption of tubal patency for female sterilisation | 1 |
| Koetsawang 1978 | Techniques for the interruption of tubal patency for female sterilisation | 1 |
| Kohaut 2004 | Techniques for the interruption of tubal patency for female sterilisation | 1 |
| Pymar 2004 | Techniques for the interruption of tubal patency for female sterilisation | 1 |
| Qui 2011 | Techniques for the interruption of tubal patency for female sterilisation | 1 |
| Rodriguez 2013 | Techniques for the interruption of tubal patency for female sterilisation | 1 |
| Siegle 2005 | Techniques for the interruption of tubal patency for female sterilisation | 1 |
| Sitompul 1984 | Techniques for the interruption of tubal patency for female sterilisation | 1 |
| Sokal 2000 | Techniques for the interruption of tubal patency for female sterilisation | 1 |
| Stovall 1991 | Techniques for the interruption of tubal patency for female sterilisation | 1 |
| Toplis 1988 | Techniques for the interruption of tubal patency for female sterilisation | 1 |
| WHO 1982 | Techniques for the interruption of tubal patency for female sterilisation | 1 |
| Yan 1990 | Techniques for the interruption of tubal patency for female sterilisation | 1 |
| Karabayir 2006 | Diuretics for transient tachypnoea of the newborn | 1 |
| Wiswell 1985 | Diuretics for transient tachypnoea of the newborn | 1 |
| ACCOG 2004 | High-dose chemotherapy and autologous bone marrow or stem cell transplantation versus conventional chemotherapy for women with early poor prognosis breast cancer | 1 |
| CALGB 2005 | High-dose chemotherapy and autologous bone marrow or stem cell transplantation versus conventional chemotherapy for women with early poor prognosis breast cancer | 1 |
| Dutch Intergp 2003 | High-dose chemotherapy and autologous bone marrow or stem cell transplantation versus conventional chemotherapy for women with early poor prognosis breast cancer | 1 |
| Dutch pilot 1998 | High-dose chemotherapy and autologous bone marrow or stem cell transplantation versus conventional chemotherapy for women with early poor prognosis breast cancer | 1 |
| ECOG 2003 | High-dose chemotherapy and autologous bone marrow or stem cell transplantation versus conventional chemotherapy for women with early poor prognosis breast cancer | 1 |
| GABG 2004 | High-dose chemotherapy and autologous bone marrow or stem cell transplantation versus conventional chemotherapy for women with early poor prognosis breast cancer | 1 |
| IBCSG 2006 | High-dose chemotherapy and autologous bone marrow or stem cell transplantation versus conventional chemotherapy for women with early poor prognosis breast cancer | 1 |
| ICCG 2005 | High-dose chemotherapy and autologous bone marrow or stem cell transplantation versus conventional chemotherapy for women with early poor prognosis breast cancer | 1 |
| JCOG 2001 | High-dose chemotherapy and autologous bone marrow or stem cell transplantation versus conventional chemotherapy for women with early poor prognosis breast cancer | 1 |
| MCG 2001 | High-dose chemotherapy and autologous bone marrow or stem cell transplantation versus conventional chemotherapy for women with early poor prognosis breast cancer | 1 |
| MDACC 2000 | High-dose chemotherapy and autologous bone marrow or stem cell transplantation versus conventional chemotherapy for women with early poor prognosis breast cancer | 1 |
| NCT00002772 | High-dose chemotherapy and autologous bone marrow or stem cell transplantation versus conventional chemotherapy for women with early poor prognosis breast cancer | 1 |
| PEGASE 01 2003 | High-dose chemotherapy and autologous bone marrow or stem cell transplantation versus conventional chemotherapy for women with early poor prognosis breast cancer | 1 |
| WSG 2005 | High-dose chemotherapy and autologous bone marrow or stem cell transplantation versus conventional chemotherapy for women with early poor prognosis breast cancer | 1 |
| Bensdorp 2015 | In vitro fertilisation for unexplained subfertility | 1 |
| Elzeiny 2014 | In vitro fertilisation for unexplained subfertility | 1 |
| Goldman 2014 | In vitro fertilisation for unexplained subfertility | 1 |
| Goverde 2000 | In vitro fertilisation for unexplained subfertility | 1 |
| Hughes 2004 | In vitro fertilisation for unexplained subfertility | 1 |
| Reindollar 2010 | In vitro fertilisation for unexplained subfertility | 1 |
| Soliman 1993 | In vitro fertilisation for unexplained subfertility | 1 |
| van Rumste 2014 | In vitro fertilisation for unexplained subfertility | 1 |
| Andreyev 2013 | Non-surgical interventions for late rectal problems (proctopathy) of radiotherapy in people who have received radiotherapy to the pelvis | 1 |
| Cavcic 2000 | Non-surgical interventions for late rectal problems (proctopathy) of radiotherapy in people who have received radiotherapy to the pelvis | 1 |
| Chruscielewska 2012 | Non-surgical interventions for late rectal problems (proctopathy) of radiotherapy in people who have received radiotherapy to the pelvis | 1 |
| Clarke 2008 | Non-surgical interventions for late rectal problems (proctopathy) of radiotherapy in people who have received radiotherapy to the pelvis | 1 |
| Ehrenpreis 2005 | Non-surgical interventions for late rectal problems (proctopathy) of radiotherapy in people who have received radiotherapy to the pelvis | 1 |
| Jensen 1997 | Non-surgical interventions for late rectal problems (proctopathy) of radiotherapy in people who have received radiotherapy to the pelvis | 1 |
| Kochhar 1991 | Non-surgical interventions for late rectal problems (proctopathy) of radiotherapy in people who have received radiotherapy to the pelvis | 1 |
| Lenz 2010 | Non-surgical interventions for late rectal problems (proctopathy) of radiotherapy in people who have received radiotherapy to the pelvis | 1 |
| Nelamangala 2012 | Non-surgical interventions for late rectal problems (proctopathy) of radiotherapy in people who have received radiotherapy to the pelvis | 1 |
| Pinto 1999 | Non-surgical interventions for late rectal problems (proctopathy) of radiotherapy in people who have received radiotherapy to the pelvis | 1 |
| Rougier 1992 | Non-surgical interventions for late rectal problems (proctopathy) of radiotherapy in people who have received radiotherapy to the pelvis | 1 |
| Sahakitrungruang 2012 | Non-surgical interventions for late rectal problems (proctopathy) of radiotherapy in people who have received radiotherapy to the pelvis | 1 |
| Talley 1997 | Non-surgical interventions for late rectal problems (proctopathy) of radiotherapy in people who have received radiotherapy to the pelvis | 1 |
| Tian 2008 | Non-surgical interventions for late rectal problems (proctopathy) of radiotherapy in people who have received radiotherapy to the pelvis | 1 |
| Venkitaraman 2008 | Non-surgical interventions for late rectal problems (proctopathy) of radiotherapy in people who have received radiotherapy to the pelvis | 1 |
| Yeoh 2013 | Non-surgical interventions for late rectal problems (proctopathy) of radiotherapy in people who have received radiotherapy to the pelvis | 1 |
| Aydemir 2011a 0000 | Prophylactic oral/topical non-absorbed antifungal agents to prevent invasive fungal infection in very low birth weight infants | 1 |
| Aydemir 2011b 0000 | Prophylactic oral/topical non-absorbed antifungal agents to prevent invasive fungal infection in very low birth weight infants | 1 |
| Mersal 2013 | Prophylactic oral/topical non-absorbed antifungal agents to prevent invasive fungal infection in very low birth weight infants | 1 |
| Ozturk 2006 | Prophylactic oral/topical non-absorbed antifungal agents to prevent invasive fungal infection in very low birth weight infants | 1 |
| Sims 1988 | Prophylactic oral/topical non-absorbed antifungal agents to prevent invasive fungal infection in very low birth weight infants | 1 |
| Violaris 2010 | Prophylactic oral/topical non-absorbed antifungal agents to prevent invasive fungal infection in very low birth weight infants | 1 |
| Wainer 1992 | Prophylactic oral/topical non-absorbed antifungal agents to prevent invasive fungal infection in very low birth weight infants | 1 |
| Agostini 2006 | Surgical approach to hysterectomy for benign gynaecological disease | 1 |
| Benassi 2002 | Surgical approach to hysterectomy for benign gynaecological disease | 1 |
| Candiani 2009 | Surgical approach to hysterectomy for benign gynaecological disease | 1 |
| Chakraborty 2011 | Surgical approach to hysterectomy for benign gynaecological disease | 1 |
| Chen 2011 | Surgical approach to hysterectomy for benign gynaecological disease | 1 |
| Darai 2001 | Surgical approach to hysterectomy for benign gynaecological disease | 1 |
| Ellstrom 1998 | Surgical approach to hysterectomy for benign gynaecological disease | 1 |
| Falcone 1999 | Surgical approach to hysterectomy for benign gynaecological disease | 1 |
| Ferrari 2000 | Surgical approach to hysterectomy for benign gynaecological disease | 1 |
| Garry 2004 | Surgical approach to hysterectomy for benign gynaecological disease | 1 |
| Ghezzi 2010 | Surgical approach to hysterectomy for benign gynaecological disease | 1 |
| Ghezzi 2011 | Surgical approach to hysterectomy for benign gynaecological disease | 1 |
| Harkki-Siren 2000 | Surgical approach to hysterectomy for benign gynaecological disease | 1 |
| Hwang 2002 | Surgical approach to hysterectomy for benign gynaecological disease | 1 |
| Jung 2011 | Surgical approach to hysterectomy for benign gynaecological disease | 1 |
| Kluivers 2007 | Surgical approach to hysterectomy for benign gynaecological disease | 1 |
| Kongwattanakul 2012 | Surgical approach to hysterectomy for benign gynaecological disease | 1 |
| Kunz 1996 | Surgical approach to hysterectomy for benign gynaecological disease | 1 |
| Langebrekke 1996 | Surgical approach to hysterectomy for benign gynaecological disease | 1 |
| Long 2002 | Surgical approach to hysterectomy for benign gynaecological disease | 1 |
| Lumsden 2000 | Surgical approach to hysterectomy for benign gynaecological disease | 1 |
| Marana 1999 | Surgical approach to hysterectomy for benign gynaecological disease | 1 |
| Miskry 2003 | Surgical approach to hysterectomy for benign gynaecological disease | 1 |
| Muzii 2007 | Surgical approach to hysterectomy for benign gynaecological disease | 1 |
| Olsson 1996 | Surgical approach to hysterectomy for benign gynaecological disease | 1 |
| Ottosen 2000 | Surgical approach to hysterectomy for benign gynaecological disease | 1 |
| Paraiso 2013 | Surgical approach to hysterectomy for benign gynaecological disease | 1 |
| Perino 1999 | Surgical approach to hysterectomy for benign gynaecological disease | 1 |
| Persson 2006 | Surgical approach to hysterectomy for benign gynaecological disease | 1 |
| Raju 1994 | Surgical approach to hysterectomy for benign gynaecological disease | 1 |
| Ribeiro 2003 | Surgical approach to hysterectomy for benign gynaecological disease | 1 |
| Richardson 1995 | Surgical approach to hysterectomy for benign gynaecological disease | 1 |
| Roy 2011 | Surgical approach to hysterectomy for benign gynaecological disease | 1 |
| Roy 2012 | Surgical approach to hysterectomy for benign gynaecological disease | 1 |
| Sarlos 2012 | Surgical approach to hysterectomy for benign gynaecological disease | 1 |
| Schutz 2002 | Surgical approach to hysterectomy for benign gynaecological disease | 1 |
| Seracchioli 2002 | Surgical approach to hysterectomy for benign gynaecological disease | 1 |
| Sesti 2008a 0000 | Surgical approach to hysterectomy for benign gynaecological disease | 1 |
| Sesti 2008b 0000 | Surgical approach to hysterectomy for benign gynaecological disease | 1 |
| Silva Filho 2006 | Surgical approach to hysterectomy for benign gynaecological disease | 1 |
| Song 2013 | Surgical approach to hysterectomy for benign gynaecological disease | 1 |
| Soriano 2001 | Surgical approach to hysterectomy for benign gynaecological disease | 1 |
| Summitt 1992 | Surgical approach to hysterectomy for benign gynaecological disease | 1 |
| Summitt 1998 | Surgical approach to hysterectomy for benign gynaecological disease | 1 |
| Tsai 2003 | Surgical approach to hysterectomy for benign gynaecological disease | 1 |
| Yuen 1998 | Surgical approach to hysterectomy for benign gynaecological disease | 1 |
| Zhu 2009 | Surgical approach to hysterectomy for benign gynaecological disease | 1 |
| Abdelmassih 2005 | Recombinant versus urinary human chorionic gonadotrophin for final oocyte maturation triggering in IVF and ICSI cycles | 1 |
| Bellavia 2013 | Recombinant versus urinary human chorionic gonadotrophin for final oocyte maturation triggering in IVF and ICSI cycles | 1 |
| Borges 2004 | Recombinant versus urinary human chorionic gonadotrophin for final oocyte maturation triggering in IVF and ICSI cycles | 1 |
| Chang 2001 | Recombinant versus urinary human chorionic gonadotrophin for final oocyte maturation triggering in IVF and ICSI cycles | 1 |
| Driscoll 2000 | Recombinant versus urinary human chorionic gonadotrophin for final oocyte maturation triggering in IVF and ICSI cycles | 1 |
| Eftekhar 2012 | Recombinant versus urinary human chorionic gonadotrophin for final oocyte maturation triggering in IVF and ICSI cycles | 1 |
| ERHCG Group 2000 | Recombinant versus urinary human chorionic gonadotrophin for final oocyte maturation triggering in IVF and ICSI cycles | 1 |
| ERLH Group 2001 | Recombinant versus urinary human chorionic gonadotrophin for final oocyte maturation triggering in IVF and ICSI cycles | 1 |
| Farrag 2008 | Recombinant versus urinary human chorionic gonadotrophin for final oocyte maturation triggering in IVF and ICSI cycles | 1 |
| Goswami 2007 | Recombinant versus urinary human chorionic gonadotrophin for final oocyte maturation triggering in IVF and ICSI cycles | 1 |
| Jie 2005 | Recombinant versus urinary human chorionic gonadotrophin for final oocyte maturation triggering in IVF and ICSI cycles | 1 |
| Kovacs 2008 | Recombinant versus urinary human chorionic gonadotrophin for final oocyte maturation triggering in IVF and ICSI cycles | 1 |
| Madani 2013 | Recombinant versus urinary human chorionic gonadotrophin for final oocyte maturation triggering in IVF and ICSI cycles | 1 |
| Manau 2002 | Recombinant versus urinary human chorionic gonadotrophin for final oocyte maturation triggering in IVF and ICSI cycles | 1 |
| Papanikolaou 2010 | Recombinant versus urinary human chorionic gonadotrophin for final oocyte maturation triggering in IVF and ICSI cycles | 1 |
| Schoolcraft 2002 | Recombinant versus urinary human chorionic gonadotrophin for final oocyte maturation triggering in IVF and ICSI cycles | 1 |
| Study 21447 | Recombinant versus urinary human chorionic gonadotrophin for final oocyte maturation triggering in IVF and ICSI cycles | 1 |
| Vidal 2005 | Recombinant versus urinary human chorionic gonadotrophin for final oocyte maturation triggering in IVF and ICSI cycles | 1 |
| Örtenstrand 1999 | Early discharge with home support of gavage feeding for stable preterm infants who have not established full oral feeds | 1 |
| Davies 1994 | Parasympathomimetic drugs for the treatment of salivary gland dysfunction due to radiotherapy | 1 |
| Johnson 1993 | Parasympathomimetic drugs for the treatment of salivary gland dysfunction due to radiotherapy | 1 |
| LeVeque 1993 | Parasympathomimetic drugs for the treatment of salivary gland dysfunction due to radiotherapy | 1 |
| Azarnoush 2009 | Self-monitoring and self-management of oral anticoagulation | 1 |
| Beyth 2000 | Self-monitoring and self-management of oral anticoagulation | 1 |
| Christensen 2006 | Self-monitoring and self-management of oral anticoagulation | 1 |
| Christensen 2011 | Self-monitoring and self-management of oral anticoagulation | 1 |
| Cromheecke 2000 | Self-monitoring and self-management of oral anticoagulation | 1 |
| Dignan 2013 | Self-monitoring and self-management of oral anticoagulation | 1 |
| Fitzmaurice 2002 | Self-monitoring and self-management of oral anticoagulation | 1 |
| Fitzmaurice 2005 | Self-monitoring and self-management of oral anticoagulation | 1 |
| Gadisseur 2003 Self mge 0000 | Self-monitoring and self-management of oral anticoagulation | 1 |
| Gadisseur 2003 Self monit 0000 | Self-monitoring and self-management of oral anticoagulation | 1 |
| Gardiner 2005 | Self-monitoring and self-management of oral anticoagulation | 1 |
| Grunau 2011 | Self-monitoring and self-management of oral anticoagulation | 1 |
| Horstkotte 1998 | Self-monitoring and self-management of oral anticoagulation | 1 |
| Kaatz Unpublished 0000 | Self-monitoring and self-management of oral anticoagulation | 1 |
| Khan 2004 | Self-monitoring and self-management of oral anticoagulation | 1 |
| Körtke 2001 | Self-monitoring and self-management of oral anticoagulation | 1 |
| Matchar 2010 | Self-monitoring and self-management of oral anticoagulation | 1 |
| Menendez-Jandula 2005 | Self-monitoring and self-management of oral anticoagulation | 1 |
| Rasmussen 2012 | Self-monitoring and self-management of oral anticoagulation | 1 |
| Ryan 2009 | Self-monitoring and self-management of oral anticoagulation | 1 |
| Sawicki 1999 | Self-monitoring and self-management of oral anticoagulation | 1 |
| Sidhu 2001 | Self-monitoring and self-management of oral anticoagulation | 1 |
| Siebenhofer 2007 | Self-monitoring and self-management of oral anticoagulation | 1 |
| Soliman Hamad 2009 | Self-monitoring and self-management of oral anticoagulation | 1 |
| Sunderji 2004 | Self-monitoring and self-management of oral anticoagulation | 1 |
| Thompson 2013 | Self-monitoring and self-management of oral anticoagulation | 1 |
| Verret 2012 | Self-monitoring and self-management of oral anticoagulation | 1 |
| Voller 2005 | Self-monitoring and self-management of oral anticoagulation | 1 |
| White 1989 | Self-monitoring and self-management of oral anticoagulation | 1 |
| Arkkila 2005 | Eradication therapy for peptic ulcer disease in Helicobacter pylori-positive people | 1 |
| Asaka 2001 | Eradication therapy for peptic ulcer disease in Helicobacter pylori-positive people | 1 |
| Avsar 1996 | Eradication therapy for peptic ulcer disease in Helicobacter pylori-positive people | 1 |
| Axon 1997 | Eradication therapy for peptic ulcer disease in Helicobacter pylori-positive people | 1 |
| Bardhan 1997 | Eradication therapy for peptic ulcer disease in Helicobacter pylori-positive people | 1 |
| Bayerdorffer 1992 | Eradication therapy for peptic ulcer disease in Helicobacter pylori-positive people | 1 |
| Bayerdorffer 1995 | Eradication therapy for peptic ulcer disease in Helicobacter pylori-positive people | 1 |
| Bayerdorffer 1996 | Eradication therapy for peptic ulcer disease in Helicobacter pylori-positive people | 1 |
| Befrits 2004 | Eradication therapy for peptic ulcer disease in Helicobacter pylori-positive people | 1 |
| Bianchi Porro 1993 | Eradication therapy for peptic ulcer disease in Helicobacter pylori-positive people | 1 |
| Bianchi Porro 1996 | Eradication therapy for peptic ulcer disease in Helicobacter pylori-positive people | 1 |
| Carpintero 1997 | Eradication therapy for peptic ulcer disease in Helicobacter pylori-positive people | 1 |
| Chen 1995 | Eradication therapy for peptic ulcer disease in Helicobacter pylori-positive people | 1 |
| Feng 2005 | Eradication therapy for peptic ulcer disease in Helicobacter pylori-positive people | 1 |
| Figueroa 1996 | Eradication therapy for peptic ulcer disease in Helicobacter pylori-positive people | 1 |
| Fukuda 1995a 0000 | Eradication therapy for peptic ulcer disease in Helicobacter pylori-positive people | 1 |
| Fukuda 1995b 0000 | Eradication therapy for peptic ulcer disease in Helicobacter pylori-positive people | 1 |
| Furuta 1995 | Eradication therapy for peptic ulcer disease in Helicobacter pylori-positive people | 1 |
| Graham 1991 | Eradication therapy for peptic ulcer disease in Helicobacter pylori-positive people | 1 |
| Graham 1992 | Eradication therapy for peptic ulcer disease in Helicobacter pylori-positive people | 1 |
| Graham 1998 | Eradication therapy for peptic ulcer disease in Helicobacter pylori-positive people | 1 |
| Harford 1996 | Eradication therapy for peptic ulcer disease in Helicobacter pylori-positive people | 1 |
| Hentschel 1993 | Eradication therapy for peptic ulcer disease in Helicobacter pylori-positive people | 1 |
| Higuchi 2003 | Eradication therapy for peptic ulcer disease in Helicobacter pylori-positive people | 1 |
| Hosking 1992 | Eradication therapy for peptic ulcer disease in Helicobacter pylori-positive people | 1 |
| Kato 1996 | Eradication therapy for peptic ulcer disease in Helicobacter pylori-positive people | 1 |
| Katoh 1995 | Eradication therapy for peptic ulcer disease in Helicobacter pylori-positive people | 1 |
| Kepecki 1999 | Eradication therapy for peptic ulcer disease in Helicobacter pylori-positive people | 1 |
| Kim 2002 | Eradication therapy for peptic ulcer disease in Helicobacter pylori-positive people | 1 |
| Lam 1997 | Eradication therapy for peptic ulcer disease in Helicobacter pylori-positive people | 1 |
| Lazzaroni 1997 | Eradication therapy for peptic ulcer disease in Helicobacter pylori-positive people | 1 |
| Lin 1994 | Eradication therapy for peptic ulcer disease in Helicobacter pylori-positive people | 1 |
| Logan 1995 | Eradication therapy for peptic ulcer disease in Helicobacter pylori-positive people | 1 |
| Malfertheiner 1999 | Eradication therapy for peptic ulcer disease in Helicobacter pylori-positive people | 1 |
| Mantzaris 1993 | Eradication therapy for peptic ulcer disease in Helicobacter pylori-positive people | 1 |
| Meining 1998 | Eradication therapy for peptic ulcer disease in Helicobacter pylori-positive people | 1 |
| Mones 2001 | Eradication therapy for peptic ulcer disease in Helicobacter pylori-positive people | 1 |
| O'Morain 1996 | Eradication therapy for peptic ulcer disease in Helicobacter pylori-positive people | 1 |
| Parente 1996 | Eradication therapy for peptic ulcer disease in Helicobacter pylori-positive people | 1 |
| Pinero 1995 | Eradication therapy for peptic ulcer disease in Helicobacter pylori-positive people | 1 |
| Pounder 1997 | Eradication therapy for peptic ulcer disease in Helicobacter pylori-positive people | 1 |
| Rauws 1990 | Eradication therapy for peptic ulcer disease in Helicobacter pylori-positive people | 1 |
| Schwartz 1998 | Eradication therapy for peptic ulcer disease in Helicobacter pylori-positive people | 1 |
| Shirotani 1996 | Eradication therapy for peptic ulcer disease in Helicobacter pylori-positive people | 1 |
| Sobhani 1995 | Eradication therapy for peptic ulcer disease in Helicobacter pylori-positive people | 1 |
| Spinzi 1994 | Eradication therapy for peptic ulcer disease in Helicobacter pylori-positive people | 1 |
| Suarez 1999 | Eradication therapy for peptic ulcer disease in Helicobacter pylori-positive people | 1 |
| Sung 1995 | Eradication therapy for peptic ulcer disease in Helicobacter pylori-positive people | 1 |
| Tomita 2002 | Eradication therapy for peptic ulcer disease in Helicobacter pylori-positive people | 1 |
| Tulassay 2008 | Eradication therapy for peptic ulcer disease in Helicobacter pylori-positive people | 1 |
| Unge 1993 | Eradication therapy for peptic ulcer disease in Helicobacter pylori-positive people | 1 |
| Van Zanten 1999 | Eradication therapy for peptic ulcer disease in Helicobacter pylori-positive people | 1 |
| Wang 1993 | Eradication therapy for peptic ulcer disease in Helicobacter pylori-positive people | 1 |
| Wang 1996 | Eradication therapy for peptic ulcer disease in Helicobacter pylori-positive people | 1 |
| Wong 1999 | Eradication therapy for peptic ulcer disease in Helicobacter pylori-positive people | 1 |
| Arrieta 2010 | Prophylactic systemic antifungal agents to prevent mortality and morbidity in very low birth weight infants | 1 |
| Aydemir 2011a 0000 | Prophylactic systemic antifungal agents to prevent mortality and morbidity in very low birth weight infants | 1 |
| Aydemir 2011b 0000 | Prophylactic systemic antifungal agents to prevent mortality and morbidity in very low birth weight infants | 1 |
| Benjamin 2014 | Prophylactic systemic antifungal agents to prevent mortality and morbidity in very low birth weight infants | 1 |
| Cabrera 2002 | Prophylactic systemic antifungal agents to prevent mortality and morbidity in very low birth weight infants | 1 |
| Kaufman 2001 | Prophylactic systemic antifungal agents to prevent mortality and morbidity in very low birth weight infants | 1 |
| Kaufman 2005 | Prophylactic systemic antifungal agents to prevent mortality and morbidity in very low birth weight infants | 1 |
| Kicklighter 2001 | Prophylactic systemic antifungal agents to prevent mortality and morbidity in very low birth weight infants | 1 |
| Kim 2010 | Prophylactic systemic antifungal agents to prevent mortality and morbidity in very low birth weight infants | 1 |
| Kirpal 2015 | Prophylactic systemic antifungal agents to prevent mortality and morbidity in very low birth weight infants | 1 |
| Manzoni 2007a 0000 | Prophylactic systemic antifungal agents to prevent mortality and morbidity in very low birth weight infants | 1 |
| Manzoni 2007b 0000 | Prophylactic systemic antifungal agents to prevent mortality and morbidity in very low birth weight infants | 1 |
| Mersal 2013 | Prophylactic systemic antifungal agents to prevent mortality and morbidity in very low birth weight infants | 1 |
| Parikh 2007 | Prophylactic systemic antifungal agents to prevent mortality and morbidity in very low birth weight infants | 1 |
| Violaris 2010 | Prophylactic systemic antifungal agents to prevent mortality and morbidity in very low birth weight infants | 1 |
| Barrington 2003 | Surgery versus medical therapy for heavy menstrual bleeding | 1 |
| Cooper 1997 | Surgery versus medical therapy for heavy menstrual bleeding | 1 |
| Crosignani 1997 | Surgery versus medical therapy for heavy menstrual bleeding | 1 |
| de Souza 2010 | Surgery versus medical therapy for heavy menstrual bleeding | 1 |
| Ergun 2012 | Surgery versus medical therapy for heavy menstrual bleeding | 1 |
| Ghazizdeh 2014 | Surgery versus medical therapy for heavy menstrual bleeding | 1 |
| Hurskainen 2001 | Surgery versus medical therapy for heavy menstrual bleeding | 1 |
| Istre 1998 | Surgery versus medical therapy for heavy menstrual bleeding | 1 |
| Kupperman 2004 | Surgery versus medical therapy for heavy menstrual bleeding | 1 |
| Malak 2006 | Surgery versus medical therapy for heavy menstrual bleeding | 1 |
| Sesti 2012 | Surgery versus medical therapy for heavy menstrual bleeding | 1 |
| Shaw 2007 | Surgery versus medical therapy for heavy menstrual bleeding | 1 |
| Soysal 2002 | Surgery versus medical therapy for heavy menstrual bleeding | 1 |
| Talis 2006 | Surgery versus medical therapy for heavy menstrual bleeding | 1 |
| Tam 2006 | Surgery versus medical therapy for heavy menstrual bleeding | 1 |
| Dyck 1986 | Plasma exchange for chronic inflammatory demyelinating polyradiculoneuropathy | 1 |
| Hahn 1996b 0000 | Plasma exchange for chronic inflammatory demyelinating polyradiculoneuropathy | 1 |
| Bachar 1999 | Individual psychological therapy in the outpatient treatment of adults with anorexia nervosa | 1 |
| Bergh 2002 | Individual psychological therapy in the outpatient treatment of adults with anorexia nervosa | 1 |
| Channon 1989 | Individual psychological therapy in the outpatient treatment of adults with anorexia nervosa | 1 |
| Dare 2001 | Individual psychological therapy in the outpatient treatment of adults with anorexia nervosa | 1 |
| Lock 2013 | Individual psychological therapy in the outpatient treatment of adults with anorexia nervosa | 1 |
| McIntosh 2005 | Individual psychological therapy in the outpatient treatment of adults with anorexia nervosa | 1 |
| Schmidt 2012 | Individual psychological therapy in the outpatient treatment of adults with anorexia nervosa | 1 |
| Serfaty 1999 | Individual psychological therapy in the outpatient treatment of adults with anorexia nervosa | 1 |
| Treasure 1995 | Individual psychological therapy in the outpatient treatment of adults with anorexia nervosa | 1 |
| Zipfel 2014 | Individual psychological therapy in the outpatient treatment of adults with anorexia nervosa | 1 |
| Altintas 2000 | Lidocaine for preventing postoperative sore throat | 1 |
| Bajaj 2004 | Lidocaine for preventing postoperative sore throat | 1 |
| Basaranoglu 2004 | Lidocaine for preventing postoperative sore throat | 1 |
| Estebe 2002 | Lidocaine for preventing postoperative sore throat | 1 |
| Estebe 2004 | Lidocaine for preventing postoperative sore throat | 1 |
| Estebe 2005 | Lidocaine for preventing postoperative sore throat | 1 |
| Hara 2005 | Lidocaine for preventing postoperative sore throat | 1 |
| Hashimoto 1981 | Lidocaine for preventing postoperative sore throat | 1 |
| Herlevsen 1992 | Lidocaine for preventing postoperative sore throat | 1 |
| Jaicobandram 2009 | Lidocaine for preventing postoperative sore throat | 1 |
| Klemola 1988 | Lidocaine for preventing postoperative sore throat | 1 |
| Krishnan 2008 | Lidocaine for preventing postoperative sore throat | 1 |
| Navarro 1997 | Lidocaine for preventing postoperative sore throat | 1 |
| Navarro 2007 | Lidocaine for preventing postoperative sore throat | 1 |
| Navarro 2012 | Lidocaine for preventing postoperative sore throat | 1 |
| Porter 1999 | Lidocaine for preventing postoperative sore throat | 1 |
| Soltani 2002 | Lidocaine for preventing postoperative sore throat | 1 |
| Takekawa 2006 | Lidocaine for preventing postoperative sore throat | 1 |
| Xu 2012 | Lidocaine for preventing postoperative sore throat | 1 |
| Arnold 1994 | High-frequency oscillatory ventilation versus conventional ventilation for acute respiratory distress syndrome | 1 |
| Bollen 2005 | High-frequency oscillatory ventilation versus conventional ventilation for acute respiratory distress syndrome | 1 |
| Demory 2007 | High-frequency oscillatory ventilation versus conventional ventilation for acute respiratory distress syndrome | 1 |
| Derdak 2002 | High-frequency oscillatory ventilation versus conventional ventilation for acute respiratory distress syndrome | 1 |
| Ferguson 2013 | High-frequency oscillatory ventilation versus conventional ventilation for acute respiratory distress syndrome | 1 |
| Mentzelopoulos 2012 | High-frequency oscillatory ventilation versus conventional ventilation for acute respiratory distress syndrome | 1 |
| Papazian 2005 | High-frequency oscillatory ventilation versus conventional ventilation for acute respiratory distress syndrome | 1 |
| Samransamruajkit 2005 | High-frequency oscillatory ventilation versus conventional ventilation for acute respiratory distress syndrome | 1 |
| Shah 2004 | High-frequency oscillatory ventilation versus conventional ventilation for acute respiratory distress syndrome | 1 |
| Young 2013 | High-frequency oscillatory ventilation versus conventional ventilation for acute respiratory distress syndrome | 1 |
| Bai 2006 | Risperidone (depot) for schizophrenia | 1 |
| Chue 2002 | Risperidone (depot) for schizophrenia | 1 |
| Covell 2012 | Risperidone (depot) for schizophrenia | 1 |
| Fleischhacker 2011 | Risperidone (depot) for schizophrenia | 1 |
| Gaebel 2010 | Risperidone (depot) for schizophrenia | 1 |
| Kane 2002 | Risperidone (depot) for schizophrenia | 1 |
| Keks 2007 | Risperidone (depot) for schizophrenia | 1 |
| Li 2011 | Risperidone (depot) for schizophrenia | 1 |
| MacFadden 2010 | Risperidone (depot) for schizophrenia | 1 |
| Pandina 2011 | Risperidone (depot) for schizophrenia | 1 |
| Quinn 2012 | Risperidone (depot) for schizophrenia | 1 |
| Rosenheck 2011 | Risperidone (depot) for schizophrenia | 1 |
| Ainsworth 2001 | Percutaneous central venous catheters versus peripheral cannulae for delivery of parenteral nutrition in neonates | 1 |
| Annibale 1995 | Percutaneous central venous catheters versus peripheral cannulae for delivery of parenteral nutrition in neonates | 1 |
| Barria 2007 | Percutaneous central venous catheters versus peripheral cannulae for delivery of parenteral nutrition in neonates | 1 |
| Hosseini 2014 | Percutaneous central venous catheters versus peripheral cannulae for delivery of parenteral nutrition in neonates | 1 |
| Janes 2000 | Percutaneous central venous catheters versus peripheral cannulae for delivery of parenteral nutrition in neonates | 1 |
| Wilson 2007 | Percutaneous central venous catheters versus peripheral cannulae for delivery of parenteral nutrition in neonates | 1 |
| Castells 2001 | Day care versus in-patient surgery for age-related cataract | 1 |
| Galin 1981 | Day care versus in-patient surgery for age-related cataract | 1 |
| Bolden 1972 | Steroids for symptom control in infectious mononucleosis | 1 |
| Collins 1984 | Steroids for symptom control in infectious mononucleosis | 1 |
| Klein 1969 | Steroids for symptom control in infectious mononucleosis | 1 |
| Prout 1966 | Steroids for symptom control in infectious mononucleosis | 1 |
| Roy 2004 | Steroids for symptom control in infectious mononucleosis | 1 |
| Simon 2003 | Steroids for symptom control in infectious mononucleosis | 1 |
| Tynell 1996 | Steroids for symptom control in infectious mononucleosis | 1 |
| Bademosi 1979 | Corticosteroids for acute bacterial meningitis | 1 |
| Belsey 1969 | Corticosteroids for acute bacterial meningitis | 1 |
| Bennett 1963 | Corticosteroids for acute bacterial meningitis | 1 |
| Bhaumik 1998 | Corticosteroids for acute bacterial meningitis | 1 |
| Ciana 1995 | Corticosteroids for acute bacterial meningitis | 1 |
| de Gans 2002 | Corticosteroids for acute bacterial meningitis | 1 |
| DeLemos 1969 | Corticosteroids for acute bacterial meningitis | 1 |
| Girgis 1989 | Corticosteroids for acute bacterial meningitis | 1 |
| Kanra 1995 | Corticosteroids for acute bacterial meningitis | 1 |
| Kilpi 1995 | Corticosteroids for acute bacterial meningitis | 1 |
| King 1994 | Corticosteroids for acute bacterial meningitis | 1 |
| Lebel 1988a 0000 | Corticosteroids for acute bacterial meningitis | 1 |
| Lebel 1988b 0000 | Corticosteroids for acute bacterial meningitis | 1 |
| Lebel 1989 | Corticosteroids for acute bacterial meningitis | 1 |
| Mathur 2013 | Corticosteroids for acute bacterial meningitis | 1 |
| Molyneux 2002 | Corticosteroids for acute bacterial meningitis | 1 |
| Nguyen 2007 | Corticosteroids for acute bacterial meningitis | 1 |
| Odio 1991 | Corticosteroids for acute bacterial meningitis | 1 |
| Peltola 2007 | Corticosteroids for acute bacterial meningitis | 1 |
| Qazi 1996 | Corticosteroids for acute bacterial meningitis | 1 |
| Sankar 2007 | Corticosteroids for acute bacterial meningitis | 1 |
| Scarborough 2007 | Corticosteroids for acute bacterial meningitis | 1 |
| Schaad 1993 | Corticosteroids for acute bacterial meningitis | 1 |
| Thomas 1999 | Corticosteroids for acute bacterial meningitis | 1 |
| Wald 1995 | Corticosteroids for acute bacterial meningitis | 1 |
| Alberts 2004 | Constraint-induced movement therapy for upper extremities in people with stroke | 1 |
| Atteya 2004 | Constraint-induced movement therapy for upper extremities in people with stroke | 1 |
| Azab 2009 | Constraint-induced movement therapy for upper extremities in people with stroke | 1 |
| Bergheim 2010 | Constraint-induced movement therapy for upper extremities in people with stroke | 1 |
| Boake 2007 | Constraint-induced movement therapy for upper extremities in people with stroke | 1 |
| Brogårdh 2009 | Constraint-induced movement therapy for upper extremities in people with stroke | 1 |
| Brunner 2012 | Constraint-induced movement therapy for upper extremities in people with stroke | 1 |
| Dahl 2008 | Constraint-induced movement therapy for upper extremities in people with stroke | 1 |
| Dromerick 2000 | Constraint-induced movement therapy for upper extremities in people with stroke | 1 |
| Dromerick 2009 | Constraint-induced movement therapy for upper extremities in people with stroke | 1 |
| Hammer 2009 | Constraint-induced movement therapy for upper extremities in people with stroke | 1 |
| Hayner 2010 | Constraint-induced movement therapy for upper extremities in people with stroke | 1 |
| Huseyinsinoglu 2012 | Constraint-induced movement therapy for upper extremities in people with stroke | 1 |
| Khan 2011 | Constraint-induced movement therapy for upper extremities in people with stroke | 1 |
| Kim 2008 | Constraint-induced movement therapy for upper extremities in people with stroke | 1 |
| Krawczyk 2012 | Constraint-induced movement therapy for upper extremities in people with stroke | 1 |
| Lin 2007 | Constraint-induced movement therapy for upper extremities in people with stroke | 1 |
| Lin 2009a 0000 | Constraint-induced movement therapy for upper extremities in people with stroke | 1 |
| Lin 2010 | Constraint-induced movement therapy for upper extremities in people with stroke | 1 |
| Myint 2008 | Constraint-induced movement therapy for upper extremities in people with stroke | 1 |
| Page 2001 | Constraint-induced movement therapy for upper extremities in people with stroke | 1 |
| Page 2002b 0000 | Constraint-induced movement therapy for upper extremities in people with stroke | 1 |
| Page 2004 | Constraint-induced movement therapy for upper extremities in people with stroke | 1 |
| Page 2005b 0000 | Constraint-induced movement therapy for upper extremities in people with stroke | 1 |
| Page 2008 | Constraint-induced movement therapy for upper extremities in people with stroke | 1 |
| Ploughman 2004 | Constraint-induced movement therapy for upper extremities in people with stroke | 1 |
| Singh 2013 | Constraint-induced movement therapy for upper extremities in people with stroke | 1 |
| Smania 2012 | Constraint-induced movement therapy for upper extremities in people with stroke | 1 |
| Suputtitada 2004 | Constraint-induced movement therapy for upper extremities in people with stroke | 1 |
| Tariah 2010 | Constraint-induced movement therapy for upper extremities in people with stroke | 1 |
| Taub 1993 | Constraint-induced movement therapy for upper extremities in people with stroke | 1 |
| Treger 2012 | Constraint-induced movement therapy for upper extremities in people with stroke | 1 |
| Van Delden 2013 | Constraint-induced movement therapy for upper extremities in people with stroke | 1 |
| Wang 2011 | Constraint-induced movement therapy for upper extremities in people with stroke | 1 |
| Wittenberg 2003 | Constraint-induced movement therapy for upper extremities in people with stroke | 1 |
| Wolf 2006 | Constraint-induced movement therapy for upper extremities in people with stroke | 1 |
| Wu 2007a 0000 | Constraint-induced movement therapy for upper extremities in people with stroke | 1 |
| Wu 2007b 0000 | Constraint-induced movement therapy for upper extremities in people with stroke | 1 |
| Wu 2007c 0000 | Constraint-induced movement therapy for upper extremities in people with stroke | 1 |
| Wu 2011 | Constraint-induced movement therapy for upper extremities in people with stroke | 1 |
| Wu 2012a 0000 | Constraint-induced movement therapy for upper extremities in people with stroke | 1 |
| Yoon 2014 | Constraint-induced movement therapy for upper extremities in people with stroke | 1 |
| Becattini 2010 | Thrombolytic therapy for pulmonary embolism | 1 |
| Dalla-Volta 1992 | Thrombolytic therapy for pulmonary embolism | 1 |
| Dotter 1979 | Thrombolytic therapy for pulmonary embolism | 1 |
| Fasullo 2011 | Thrombolytic therapy for pulmonary embolism | 1 |
| Goldhaber 1993 | Thrombolytic therapy for pulmonary embolism | 1 |
| Jerjes-Sánchez 1995 | Thrombolytic therapy for pulmonary embolism | 1 |
| Kline 2014 | Thrombolytic therapy for pulmonary embolism | 1 |
| Konstantinides 2002 | Thrombolytic therapy for pulmonary embolism | 1 |
| Kucher 2014 | Thrombolytic therapy for pulmonary embolism | 1 |
| Levine 1990 | Thrombolytic therapy for pulmonary embolism | 1 |
| Ly 1978 | Thrombolytic therapy for pulmonary embolism | 1 |
| Marini 1988 | Thrombolytic therapy for pulmonary embolism | 1 |
| Meyer 2014 | Thrombolytic therapy for pulmonary embolism | 1 |
| PIOPED 1990 | Thrombolytic therapy for pulmonary embolism | 1 |
| Sharifi 2013 | Thrombolytic therapy for pulmonary embolism | 1 |
| Taherkhani 2014 | Thrombolytic therapy for pulmonary embolism | 1 |
| Tibbutt 1974 | Thrombolytic therapy for pulmonary embolism | 1 |
| UPETSG 1970 | Thrombolytic therapy for pulmonary embolism | 1 |
| Arrillaga 1999 | Emergency ultrasound-based algorithms for diagnosing blunt abdominal trauma | 1 |
| Boulanger 1999 | Emergency ultrasound-based algorithms for diagnosing blunt abdominal trauma | 1 |
| Melniker 2006 | Emergency ultrasound-based algorithms for diagnosing blunt abdominal trauma | 1 |
| Rose 2001 | Emergency ultrasound-based algorithms for diagnosing blunt abdominal trauma | 1 |
| Astermark 2007 | Recombinant factor VIIa concentrate versus plasma-derived concentrates for treating acute bleeding episodes in people with haemophilia and inhibitors | 1 |
| Young 2008 | Recombinant factor VIIa concentrate versus plasma-derived concentrates for treating acute bleeding episodes in people with haemophilia and inhibitors | 1 |
| Albright 1991 | Intrathecal baclofen for treating spasticity in children with cerebral palsy | 1 |
| Armstrong 1997 | Intrathecal baclofen for treating spasticity in children with cerebral palsy | 1 |
| Gilmartin 2000 | Intrathecal baclofen for treating spasticity in children with cerebral palsy | 1 |
| Hoving 2006 | Intrathecal baclofen for treating spasticity in children with cerebral palsy | 1 |
| Hoving 2007 | Intrathecal baclofen for treating spasticity in children with cerebral palsy | 1 |
| Hoving 2009a 0000 | Intrathecal baclofen for treating spasticity in children with cerebral palsy | 1 |
| Ballmann 2014 | Insulin and oral agents for managing cystic fibrosis-related diabetes | 1 |
| Grover 2008 | Insulin and oral agents for managing cystic fibrosis-related diabetes | 1 |
| Moran 2001 | Insulin and oral agents for managing cystic fibrosis-related diabetes | 1 |
| Moran 2009 | Insulin and oral agents for managing cystic fibrosis-related diabetes | 1 |
| FRISC-II 0000 | Routine invasive strategies versus selective invasive strategies for unstable angina and non-ST elevation myocardial infarction in the stent era | 1 |
| ICTUS 0000 | Routine invasive strategies versus selective invasive strategies for unstable angina and non-ST elevation myocardial infarction in the stent era | 1 |
| Italian Elderly ACS 0000 | Routine invasive strategies versus selective invasive strategies for unstable angina and non-ST elevation myocardial infarction in the stent era | 1 |
| LIPSIA-NSTEMI 0000 | Routine invasive strategies versus selective invasive strategies for unstable angina and non-ST elevation myocardial infarction in the stent era | 1 |
| OASIS 5 0000 | Routine invasive strategies versus selective invasive strategies for unstable angina and non-ST elevation myocardial infarction in the stent era | 1 |
| RITA-3 0000 | Routine invasive strategies versus selective invasive strategies for unstable angina and non-ST elevation myocardial infarction in the stent era | 1 |
| TACTICS-TIMI 18 0000 | Routine invasive strategies versus selective invasive strategies for unstable angina and non-ST elevation myocardial infarction in the stent era | 1 |
| VINO 0000 | Routine invasive strategies versus selective invasive strategies for unstable angina and non-ST elevation myocardial infarction in the stent era | 1 |
| Dekleva 2004 | Hyperbaric oxygen therapy for acute coronary syndrome | 1 |
| Dotsenko 2007 | Hyperbaric oxygen therapy for acute coronary syndrome | 1 |
| Hot MI 1998 | Hyperbaric oxygen therapy for acute coronary syndrome | 1 |
| Sharifi 2004 | Hyperbaric oxygen therapy for acute coronary syndrome | 1 |
| Swift 1992 | Hyperbaric oxygen therapy for acute coronary syndrome | 1 |
| Thurston 1973 | Hyperbaric oxygen therapy for acute coronary syndrome | 1 |
| Arvola 1999 | Probiotics for the prevention of pediatric antibiotic-associated diarrhea | 1 |
| Benhamou 1999 | Probiotics for the prevention of pediatric antibiotic-associated diarrhea | 1 |
| Contardi 1991 | Probiotics for the prevention of pediatric antibiotic-associated diarrhea | 1 |
| Conway 2007 | Probiotics for the prevention of pediatric antibiotic-associated diarrhea | 1 |
| Correa 2005 | Probiotics for the prevention of pediatric antibiotic-associated diarrhea | 1 |
| Destura unpublished 0000 | Probiotics for the prevention of pediatric antibiotic-associated diarrhea | 1 |
| Erdeve 2004 | Probiotics for the prevention of pediatric antibiotic-associated diarrhea | 1 |
| Fox 2015 | Probiotics for the prevention of pediatric antibiotic-associated diarrhea | 1 |
| Georgieva unpublished 0000 | Probiotics for the prevention of pediatric antibiotic-associated diarrhea | 1 |
| Jirapinyo 2002 | Probiotics for the prevention of pediatric antibiotic-associated diarrhea | 1 |
| Kodadad 2013 | Probiotics for the prevention of pediatric antibiotic-associated diarrhea | 1 |
| Kotowska 2005 | Probiotics for the prevention of pediatric antibiotic-associated diarrhea | 1 |
| LaRosa 2003 | Probiotics for the prevention of pediatric antibiotic-associated diarrhea | 1 |
| Merenstein 2009 | Probiotics for the prevention of pediatric antibiotic-associated diarrhea | 1 |
| Ruszczynski 2008 | Probiotics for the prevention of pediatric antibiotic-associated diarrhea | 1 |
| Saneeyan 2011 | Probiotics for the prevention of pediatric antibiotic-associated diarrhea | 1 |
| Shan 2013 | Probiotics for the prevention of pediatric antibiotic-associated diarrhea | 1 |
| Sykora 2005 | Probiotics for the prevention of pediatric antibiotic-associated diarrhea | 1 |
| Szajewska 2009 | Probiotics for the prevention of pediatric antibiotic-associated diarrhea | 1 |
| Szymanski 2008 | Probiotics for the prevention of pediatric antibiotic-associated diarrhea | 1 |
| Tankanow 1990 | Probiotics for the prevention of pediatric antibiotic-associated diarrhea | 1 |
| Vanderhoof 1999 | Probiotics for the prevention of pediatric antibiotic-associated diarrhea | 1 |
| Zheng 2012 | Probiotics for the prevention of pediatric antibiotic-associated diarrhea | 1 |
| Aviram 1992 | Chest physiotherapy for acute bronchiolitis in paediatric patients between 0 and 24 months old | 1 |
| Bohe 2004 | Chest physiotherapy for acute bronchiolitis in paediatric patients between 0 and 24 months old | 1 |
| De Córdoba 2008 | Chest physiotherapy for acute bronchiolitis in paediatric patients between 0 and 24 months old | 1 |
| Gajdos 2010 | Chest physiotherapy for acute bronchiolitis in paediatric patients between 0 and 24 months old | 1 |
| Gomes 2012 | Chest physiotherapy for acute bronchiolitis in paediatric patients between 0 and 24 months old | 1 |
| Lopez Galbany 2004 | Chest physiotherapy for acute bronchiolitis in paediatric patients between 0 and 24 months old | 1 |
| Nicholas 1999 | Chest physiotherapy for acute bronchiolitis in paediatric patients between 0 and 24 months old | 1 |
| Postiaux 2011 | Chest physiotherapy for acute bronchiolitis in paediatric patients between 0 and 24 months old | 1 |
| Remondini 2014 | Chest physiotherapy for acute bronchiolitis in paediatric patients between 0 and 24 months old | 1 |
| Rochat 2010 | Chest physiotherapy for acute bronchiolitis in paediatric patients between 0 and 24 months old | 1 |
| Sanchez Bayle 2012 | Chest physiotherapy for acute bronchiolitis in paediatric patients between 0 and 24 months old | 1 |
| Webb 1985 | Chest physiotherapy for acute bronchiolitis in paediatric patients between 0 and 24 months old | 1 |
| El-Chimi 2017 | Sustained versus standard inflations during neonatal resuscitation to prevent mortality and improve respiratory outcomes | 1 |
| Jiravisitkul 2017 | Sustained versus standard inflations during neonatal resuscitation to prevent mortality and improve respiratory outcomes | 1 |
| Lindner 2005 | Sustained versus standard inflations during neonatal resuscitation to prevent mortality and improve respiratory outcomes | 1 |
| Lista 2015 | Sustained versus standard inflations during neonatal resuscitation to prevent mortality and improve respiratory outcomes | 1 |
| Mercadante 2016 | Sustained versus standard inflations during neonatal resuscitation to prevent mortality and improve respiratory outcomes | 1 |
| Ngan 2017 | Sustained versus standard inflations during neonatal resuscitation to prevent mortality and improve respiratory outcomes | 1 |
| Schmölzer 2015 | Sustained versus standard inflations during neonatal resuscitation to prevent mortality and improve respiratory outcomes | 1 |
| Schwaberger 2015 | Sustained versus standard inflations during neonatal resuscitation to prevent mortality and improve respiratory outcomes | 1 |
| Nagle 2000 | Antibiotic use for irreversible pulpitis | 1 |
| Annane 2004 | Hyperbaric oxygen therapy for late radiation tissue injury | 1 |
| Clarke 2008 | Hyperbaric oxygen therapy for late radiation tissue injury | 1 |
| Gothard 2010 | Hyperbaric oxygen therapy for late radiation tissue injury | 1 |
| Hulshof 2002 | Hyperbaric oxygen therapy for late radiation tissue injury | 1 |
| Marx 1985 | Hyperbaric oxygen therapy for late radiation tissue injury | 1 |
| Marx 1999a 0000 | Hyperbaric oxygen therapy for late radiation tissue injury | 1 |
| Marx 1999b 0000 | Hyperbaric oxygen therapy for late radiation tissue injury | 1 |
| Oton Sanchez 2013 | Hyperbaric oxygen therapy for late radiation tissue injury | 1 |
| Pritchard 2001 | Hyperbaric oxygen therapy for late radiation tissue injury | 1 |
| Schoen 2007 | Hyperbaric oxygen therapy for late radiation tissue injury | 1 |
| Shao 2011 | Hyperbaric oxygen therapy for late radiation tissue injury | 1 |
| Sidik 2007 | Hyperbaric oxygen therapy for late radiation tissue injury | 1 |
| Svalestad 2014 | Hyperbaric oxygen therapy for late radiation tissue injury | 1 |
| Teguh 2009 | Hyperbaric oxygen therapy for late radiation tissue injury | 1 |
| Constantino 2005 | Advocacy interventions to reduce or eliminate violence and promote the physical and psychosocial well-being of women who experience intimate partner abuse | 1 |
| Cripe 2010 | Advocacy interventions to reduce or eliminate violence and promote the physical and psychosocial well-being of women who experience intimate partner abuse | 1 |
| Gillum 2009 | Advocacy interventions to reduce or eliminate violence and promote the physical and psychosocial well-being of women who experience intimate partner abuse | 1 |
| Hyman 2001 | Advocacy interventions to reduce or eliminate violence and promote the physical and psychosocial well-being of women who experience intimate partner abuse | 1 |
| Kiely 2010 | Advocacy interventions to reduce or eliminate violence and promote the physical and psychosocial well-being of women who experience intimate partner abuse | 1 |
| McFarlane 2000 | Advocacy interventions to reduce or eliminate violence and promote the physical and psychosocial well-being of women who experience intimate partner abuse | 1 |
| McFarlane 2006 | Advocacy interventions to reduce or eliminate violence and promote the physical and psychosocial well-being of women who experience intimate partner abuse | 1 |
| Sullivan 1991 | Advocacy interventions to reduce or eliminate violence and promote the physical and psychosocial well-being of women who experience intimate partner abuse | 1 |
| Sullivan 1992 | Advocacy interventions to reduce or eliminate violence and promote the physical and psychosocial well-being of women who experience intimate partner abuse | 1 |
| Sullivan 2002 | Advocacy interventions to reduce or eliminate violence and promote the physical and psychosocial well-being of women who experience intimate partner abuse | 1 |
| Taft 2011 | Advocacy interventions to reduce or eliminate violence and promote the physical and psychosocial well-being of women who experience intimate partner abuse | 1 |
| Tiwari 2005 | Advocacy interventions to reduce or eliminate violence and promote the physical and psychosocial well-being of women who experience intimate partner abuse | 1 |
| Tiwari 2010 | Advocacy interventions to reduce or eliminate violence and promote the physical and psychosocial well-being of women who experience intimate partner abuse | 1 |
| Gottschlich 2002 | Nutritional support for critically ill children | 1 |
| Carman 2000 | Influenza vaccination for healthcare workers who care for people aged 60 or older living in long-term care institutions | 1 |
| Hayward 2006 | Influenza vaccination for healthcare workers who care for people aged 60 or older living in long-term care institutions | 1 |
| Lemaitre 2009 | Influenza vaccination for healthcare workers who care for people aged 60 or older living in long-term care institutions | 1 |
| Oshitani 2000 | Influenza vaccination for healthcare workers who care for people aged 60 or older living in long-term care institutions | 1 |
| Potter 1997 | Influenza vaccination for healthcare workers who care for people aged 60 or older living in long-term care institutions | 1 |
| Aarons 2000 | Interventions for preventing unintended pregnancies among adolescents | 1 |
| Allen 1997 | Interventions for preventing unintended pregnancies among adolescents | 1 |
| Baird 2010 | Interventions for preventing unintended pregnancies among adolescents | 1 |
| Basen-Engquist 2001 | Interventions for preventing unintended pregnancies among adolescents | 1 |
| Black 2006 | Interventions for preventing unintended pregnancies among adolescents | 1 |
| Blake 2001 | Interventions for preventing unintended pregnancies among adolescents | 1 |
| Bonell 2013 | Interventions for preventing unintended pregnancies among adolescents | 1 |
| Borgia 2005 | Interventions for preventing unintended pregnancies among adolescents | 1 |
| Cabezon 2005 | Interventions for preventing unintended pregnancies among adolescents | 1 |
| Clark 2005 | Interventions for preventing unintended pregnancies among adolescents | 1 |
| Coyle 1999 | Interventions for preventing unintended pregnancies among adolescents | 1 |
| Coyle 2004 | Interventions for preventing unintended pregnancies among adolescents | 1 |
| Coyle 2006 | Interventions for preventing unintended pregnancies among adolescents | 1 |
| Diclemente 2004 | Interventions for preventing unintended pregnancies among adolescents | 1 |
| Dilorio 2006 | Interventions for preventing unintended pregnancies among adolescents | 1 |
| Dilorio 2007 | Interventions for preventing unintended pregnancies among adolescents | 1 |
| Downs 2004 | Interventions for preventing unintended pregnancies among adolescents | 1 |
| Eisen 1990 | Interventions for preventing unintended pregnancies among adolescents | 1 |
| Fawole 1999 | Interventions for preventing unintended pregnancies among adolescents | 1 |
| Ferguson 1998 | Interventions for preventing unintended pregnancies among adolescents | 1 |
| Graham 2002 | Interventions for preventing unintended pregnancies among adolescents | 1 |
| Guilamo-Ramos 2011b 0000 | Interventions for preventing unintended pregnancies among adolescents | 1 |
| Henderson 2007 | Interventions for preventing unintended pregnancies among adolescents | 1 |
| Herceg-Brown 1986 | Interventions for preventing unintended pregnancies among adolescents | 1 |
| Howard 1990 | Interventions for preventing unintended pregnancies among adolescents | 1 |
| Jemmott 1998 | Interventions for preventing unintended pregnancies among adolescents | 1 |
| Jemmott 2005 | Interventions for preventing unintended pregnancies among adolescents | 1 |
| Jemmott 2010 | Interventions for preventing unintended pregnancies among adolescents | 1 |
| Kirby 1997a 0000 | Interventions for preventing unintended pregnancies among adolescents | 1 |
| Kirby 1997b 0000 | Interventions for preventing unintended pregnancies among adolescents | 1 |
| Kirby 2004 | Interventions for preventing unintended pregnancies among adolescents | 1 |
| Kogan 2012 | Interventions for preventing unintended pregnancies among adolescents | 1 |
| Markham 2012 | Interventions for preventing unintended pregnancies among adolescents | 1 |
| Mba 2007 | Interventions for preventing unintended pregnancies among adolescents | 1 |
| Minnis 2014 | Interventions for preventing unintended pregnancies among adolescents | 1 |
| Mitchell-DiCenso 1997 | Interventions for preventing unintended pregnancies among adolescents | 1 |
| Morberg 1998 | Interventions for preventing unintended pregnancies among adolescents | 1 |
| Morrison-Beedy 2013 | Interventions for preventing unintended pregnancies among adolescents | 1 |
| Norton 2012 | Interventions for preventing unintended pregnancies among adolescents | 1 |
| O'Donnell 1999 | Interventions for preventing unintended pregnancies among adolescents | 1 |
| O'Donnell 2002 | Interventions for preventing unintended pregnancies among adolescents | 1 |
| Okonofua 2003 | Interventions for preventing unintended pregnancies among adolescents | 1 |
| Perskin 2015 | Interventions for preventing unintended pregnancies among adolescents | 1 |
| Philliber 2002 | Interventions for preventing unintended pregnancies among adolescents | 1 |
| Raine 2005 | Interventions for preventing unintended pregnancies among adolescents | 1 |
| Raymond 2006 | Interventions for preventing unintended pregnancies among adolescents | 1 |
| Shrier 2001 | Interventions for preventing unintended pregnancies among adolescents | 1 |
| Sieving 2011 | Interventions for preventing unintended pregnancies among adolescents | 1 |
| Smith 1994 | Interventions for preventing unintended pregnancies among adolescents | 1 |
| Stephenson 2004 | Interventions for preventing unintended pregnancies among adolescents | 1 |
| Villarruel 2006 | Interventions for preventing unintended pregnancies among adolescents | 1 |
| Walker 2006 | Interventions for preventing unintended pregnancies among adolescents | 1 |
| Wight 2002 | Interventions for preventing unintended pregnancies among adolescents | 1 |
| Ahonen 2004 | Drugs for the acute treatment of migraine in children and adolescents | 1 |
| Ahonen 2006 | Drugs for the acute treatment of migraine in children and adolescents | 1 |
| Callenbach 2007 | Drugs for the acute treatment of migraine in children and adolescents | 1 |
| Derosier 2012 | Drugs for the acute treatment of migraine in children and adolescents | 1 |
| Evers 2006 | Drugs for the acute treatment of migraine in children and adolescents | 1 |
| Fujita 2014 | Drugs for the acute treatment of migraine in children and adolescents | 1 |
| Ho 2012 | Drugs for the acute treatment of migraine in children and adolescents | 1 |
| Hainen 1997a 0000 | Drugs for the acute treatment of migraine in children and adolescents | 1 |
| Hainen 1997b 0000 | Drugs for the acute treatment of migraine in children and adolescents | 1 |
| Hainen 1997c 0000 | Drugs for the acute treatment of migraine in children and adolescents | 1 |
| Hainen 2002 | Drugs for the acute treatment of migraine in children and adolescents | 1 |
| Lewis 2002 | Drugs for the acute treatment of migraine in children and adolescents | 1 |
| Lewis 2007 | Drugs for the acute treatment of migraine in children and adolescents | 1 |
| Linder 2008 | Drugs for the acute treatment of migraine in children and adolescents | 1 |
| NCT01211145 | Drugs for the acute treatment of migraine in children and adolescents | 1 |
| Rothner 1997 | Drugs for the acute treatment of migraine in children and adolescents | 1 |
| Rothner 1999a 0000 | Drugs for the acute treatment of migraine in children and adolescents | 1 |
| Rothner 1999b 0000 | Drugs for the acute treatment of migraine in children and adolescents | 1 |
| Rothner 1999c 0000 | Drugs for the acute treatment of migraine in children and adolescents | 1 |
| Rothner 2006 | Drugs for the acute treatment of migraine in children and adolescents | 1 |
| Ueberall 1999 | Drugs for the acute treatment of migraine in children and adolescents | 1 |
| Visser 2004a 0000 | Drugs for the acute treatment of migraine in children and adolescents | 1 |
| Winner 1997 | Drugs for the acute treatment of migraine in children and adolescents | 1 |
| Winner 2000 | Drugs for the acute treatment of migraine in children and adolescents | 1 |
| Winner 2002 | Drugs for the acute treatment of migraine in children and adolescents | 1 |
| Winner 2006 | Drugs for the acute treatment of migraine in children and adolescents | 1 |
| Winner 2007 | Drugs for the acute treatment of migraine in children and adolescents | 1 |
| Bennion 1991 | Intravenous in-line filters for preventing morbidity and mortality in neonates | 1 |
| Thomas 1989 | Intravenous in-line filters for preventing morbidity and mortality in neonates | 1 |
| van den Hoogen 2006 | Intravenous in-line filters for preventing morbidity and mortality in neonates | 1 |
| van Lingen 2004 | Intravenous in-line filters for preventing morbidity and mortality in neonates | 1 |
| Collinge 1982 | Responsive versus scheduled feeding for preterm infants | 1 |
| Kansas 2004 | Responsive versus scheduled feeding for preterm infants | 1 |
| McCain 2001 | Responsive versus scheduled feeding for preterm infants | 1 |
| McCain 2012 | Responsive versus scheduled feeding for preterm infants | 1 |
| Pridham 1999 | Responsive versus scheduled feeding for preterm infants | 1 |
| Pridham 2001 | Responsive versus scheduled feeding for preterm infants | 1 |
| Puckett 2008 | Responsive versus scheduled feeding for preterm infants | 1 |
| Saunders 1991 | Responsive versus scheduled feeding for preterm infants | 1 |
| Waber 1998 | Responsive versus scheduled feeding for preterm infants | 1 |
| APIP 1998 | Early developmental intervention programmes provided post hospital discharge to prevent motor and cognitive impairment in preterm infants | 1 |
| Dusing 2015 | Early developmental intervention programmes provided post hospital discharge to prevent motor and cognitive impairment in preterm infants | 1 |
| Koldewijn 2009 | Early developmental intervention programmes provided post hospital discharge to prevent motor and cognitive impairment in preterm infants | 1 |
| Kyno 2012 | Early developmental intervention programmes provided post hospital discharge to prevent motor and cognitive impairment in preterm infants | 1 |
| Lekskulchai 2001 | Early developmental intervention programmes provided post hospital discharge to prevent motor and cognitive impairment in preterm infants | 1 |
| Melnyk 2001 | Early developmental intervention programmes provided post hospital discharge to prevent motor and cognitive impairment in preterm infants | 1 |
| Nelson 2001 | Early developmental intervention programmes provided post hospital discharge to prevent motor and cognitive impairment in preterm infants | 1 |
| Nurcombe 1984 | Early developmental intervention programmes provided post hospital discharge to prevent motor and cognitive impairment in preterm infants | 1 |
| Ohgi 2004 | Early developmental intervention programmes provided post hospital discharge to prevent motor and cognitive impairment in preterm infants | 1 |
| Resnick 1988 | Early developmental intervention programmes provided post hospital discharge to prevent motor and cognitive impairment in preterm infants | 1 |
| Rice 1979 | Early developmental intervention programmes provided post hospital discharge to prevent motor and cognitive impairment in preterm infants | 1 |
| Sajaniemi 2001 | Early developmental intervention programmes provided post hospital discharge to prevent motor and cognitive impairment in preterm infants | 1 |
| Spittle 2009 | Early developmental intervention programmes provided post hospital discharge to prevent motor and cognitive impairment in preterm infants | 1 |
| Teti 2009 | Early developmental intervention programmes provided post hospital discharge to prevent motor and cognitive impairment in preterm infants | 1 |
| Wu 2014 | Early developmental intervention programmes provided post hospital discharge to prevent motor and cognitive impairment in preterm infants | 1 |
| Arnold 2008 | Aquatic exercise for the treatment of knee and hip osteoarthritis | 1 |
| Cochrane 2005 | Aquatic exercise for the treatment of knee and hip osteoarthritis | 1 |
| Foley 2003 | Aquatic exercise for the treatment of knee and hip osteoarthritis | 1 |
| Fransen 2007 | Aquatic exercise for the treatment of knee and hip osteoarthritis | 1 |
| Hale 2012 | Aquatic exercise for the treatment of knee and hip osteoarthritis | 1 |
| Hinman 2007 | Aquatic exercise for the treatment of knee and hip osteoarthritis | 1 |
| Kim 2012 | Aquatic exercise for the treatment of knee and hip osteoarthritis | 1 |
| Lim 2010 | Aquatic exercise for the treatment of knee and hip osteoarthritis | 1 |
| Lund 2008 | Aquatic exercise for the treatment of knee and hip osteoarthritis | 1 |
| Patrick 2001 | Aquatic exercise for the treatment of knee and hip osteoarthritis | 1 |
| Stener-Victorin 2004 | Aquatic exercise for the treatment of knee and hip osteoarthritis | 1 |
| Wang 2006 | Aquatic exercise for the treatment of knee and hip osteoarthritis | 1 |
| Wang 2011 | Aquatic exercise for the treatment of knee and hip osteoarthritis | 1 |
| Alton 1999 | Topical cystic fibrosis transmembrane conductance regulator gene replacement for cystic fibrosis-related lung disease | 1 |
| Alton 2015 | Topical cystic fibrosis transmembrane conductance regulator gene replacement for cystic fibrosis-related lung disease | 1 |
| Moss 2004 | Topical cystic fibrosis transmembrane conductance regulator gene replacement for cystic fibrosis-related lung disease | 1 |
| Moss 2007 | Topical cystic fibrosis transmembrane conductance regulator gene replacement for cystic fibrosis-related lung disease | 1 |
| Redman 1994 | Interval debulking surgery for advanced epithelial ovarian cancer | 1 |
| Rose 2004 | Interval debulking surgery for advanced epithelial ovarian cancer | 1 |
| Van der Burg 1995 | Interval debulking surgery for advanced epithelial ovarian cancer | 1 |
| Bloechle 1999 | Pylorus-preserving pancreaticoduodenectomy (pp Whipple) versus pancreaticoduodenectomy (classic Whipple) for surgical treatment of periampullary and pancreatic carcinoma | 1 |
| Lin 1999 | Pylorus-preserving pancreaticoduodenectomy (pp Whipple) versus pancreaticoduodenectomy (classic Whipple) for surgical treatment of periampullary and pancreatic carcinoma | 1 |
| Paquet 1998 | Pylorus-preserving pancreaticoduodenectomy (pp Whipple) versus pancreaticoduodenectomy (classic Whipple) for surgical treatment of periampullary and pancreatic carcinoma | 1 |
| Seiler 2005 | Pylorus-preserving pancreaticoduodenectomy (pp Whipple) versus pancreaticoduodenectomy (classic Whipple) for surgical treatment of periampullary and pancreatic carcinoma | 1 |
| Srinarmwong 2008 | Pylorus-preserving pancreaticoduodenectomy (pp Whipple) versus pancreaticoduodenectomy (classic Whipple) for surgical treatment of periampullary and pancreatic carcinoma | 1 |
| Taher 2015 | Pylorus-preserving pancreaticoduodenectomy (pp Whipple) versus pancreaticoduodenectomy (classic Whipple) for surgical treatment of periampullary and pancreatic carcinoma | 1 |
| Tran 2004 | Pylorus-preserving pancreaticoduodenectomy (pp Whipple) versus pancreaticoduodenectomy (classic Whipple) for surgical treatment of periampullary and pancreatic carcinoma | 1 |
| Wenger 1999 | Pylorus-preserving pancreaticoduodenectomy (pp Whipple) versus pancreaticoduodenectomy (classic Whipple) for surgical treatment of periampullary and pancreatic carcinoma | 1 |
| Armijos 0000 | Nutritional supplements for people being treated for active tuberculosis | 1 |
| Daley 0000 | Nutritional supplements for people being treated for active tuberculosis | 1 |
| Farazi 2015 | Nutritional supplements for people being treated for active tuberculosis | 1 |
| Ginawi 2013 | Nutritional supplements for people being treated for active tuberculosis | 1 |
| Hanekom 1997 | Nutritional supplements for people being treated for active tuberculosis | 1 |
| Jahnavi 2010 | Nutritional supplements for people being treated for active tuberculosis | 1 |
| Jeremiah 2014 | Nutritional supplements for people being treated for active tuberculosis | 1 |
| Karyadi 2002 | Nutritional supplements for people being treated for active tuberculosis | 1 |
| Kota 2011 | Nutritional supplements for people being treated for active tuberculosis | 1 |
| Lawson 2010 | Nutritional supplements for people being treated for active tuberculosis | 1 |
| Lodha 2014 | Nutritional supplements for people being treated for active tuberculosis | 1 |
| Martineau 2011 | Nutritional supplements for people being treated for active tuberculosis | 1 |
| Martins 2009 | Nutritional supplements for people being treated for active tuberculosis | 1 |
| Mehta 2011 | Nutritional supplements for people being treated for active tuberculosis | 1 |
| Mily 2015 | Nutritional supplements for people being treated for active tuberculosis | 1 |
| Morcos 1998 | Nutritional supplements for people being treated for active tuberculosis | 1 |
| Nursyam 2006 | Nutritional supplements for people being treated for active tuberculosis | 1 |
| Pakasi 2010 | Nutritional supplements for people being treated for active tuberculosis | 1 |
| Paliliewu 2013 | Nutritional supplements for people being treated for active tuberculosis | 1 |
| Paton 2004 | Nutritional supplements for people being treated for active tuberculosis | 1 |
| Praygod 2011 | Nutritional supplements for people being treated for active tuberculosis | 1 |
| Praygod 2011 | Nutritional supplements for people being treated for active tuberculosis | 1 |
| Pérez-Guzmán 2005 | Nutritional supplements for people being treated for active tuberculosis | 1 |
| Ralph 2013 | Nutritional supplements for people being treated for active tuberculosis | 1 |
| Range 2005 | Nutritional supplements for people being treated for active tuberculosis | 1 |
| Schön 2003 | Nutritional supplements for people being treated for active tuberculosis | 1 |
| Schön 2011 | Nutritional supplements for people being treated for active tuberculosis | 1 |
| Semba 2007 | Nutritional supplements for people being treated for active tuberculosis | 1 |
| Seyedrezazadeh 2006 | Nutritional supplements for people being treated for active tuberculosis | 1 |
| Singh 2013 | Nutritional supplements for people being treated for active tuberculosis | 1 |
| Sudarsanam 2010 | Nutritional supplements for people being treated for active tuberculosis | 1 |
| Tukvadze 2015 | Nutritional supplements for people being treated for active tuberculosis | 1 |
| Villamor 2008 | Nutritional supplements for people being treated for active tuberculosis | 1 |
| Visser 2011 | Nutritional supplements for people being treated for active tuberculosis | 1 |
| Wejse 2008 | Nutritional supplements for people being treated for active tuberculosis | 1 |
| Scharfenberg 1971 | Nicotine receptor partial agonists for smoking cessation | 1 |
| Vinnikov 2008 | Nicotine receptor partial agonists for smoking cessation | 1 |
| West 2011 | Nicotine receptor partial agonists for smoking cessation | 1 |
| Walker 2014 | Nicotine receptor partial agonists for smoking cessation | 1 |
| Tonstad 2011 | Nicotine receptor partial agonists for smoking cessation | 1 |
| Anthenelli 2013 | Nicotine receptor partial agonists for smoking cessation | 1 |
| Aubin 2008 | Nicotine receptor partial agonists for smoking cessation | 1 |
| Baker 2016 | Nicotine receptor partial agonists for smoking cessation | 1 |
| Bolliger 2011 | Nicotine receptor partial agonists for smoking cessation | 1 |
| Carson 2014 | Nicotine receptor partial agonists for smoking cessation | 1 |
| Chengappa 2014 | Nicotine receptor partial agonists for smoking cessation | 1 |
| Cinciripini 2013 | Nicotine receptor partial agonists for smoking cessation | 1 |
| De Dios 2012 | Nicotine receptor partial agonists for smoking cessation | 1 |
| EAGLES 2016 | Nicotine receptor partial agonists for smoking cessation | 1 |
| Ebbert 2015 | Nicotine receptor partial agonists for smoking cessation | 1 |
| Eisenberg 2016 | Nicotine receptor partial agonists for smoking cessation | 1 |
| Evins 2014 | Nicotine receptor partial agonists for smoking cessation | 1 |
| Gonzales 2006 | Nicotine receptor partial agonists for smoking cessation | 1 |
| Gonzales 2014 | Nicotine receptor partial agonists for smoking cessation | 1 |
| Hajek 2015 | Nicotine receptor partial agonists for smoking cessation | 1 |
| Heydari 2012 | Nicotine receptor partial agonists for smoking cessation | 1 |
| Jorenby 2006 | Nicotine receptor partial agonists for smoking cessation | 1 |
| Nahvi 2014a 0000 | Nicotine receptor partial agonists for smoking cessation | 1 |
| Nakamura 2007 | Nicotine receptor partial agonists for smoking cessation | 1 |
| NCT00828113 | Nicotine receptor partial agonists for smoking cessation | 1 |
| NCT01347112 | Nicotine receptor partial agonists for smoking cessation | 1 |
| Niaura 2008 | Nicotine receptor partial agonists for smoking cessation | 1 |
| Nides 2006 | Nicotine receptor partial agonists for smoking cessation | 1 |
| Oncken 2006 | Nicotine receptor partial agonists for smoking cessation | 1 |
| Rennard 2012 | Nicotine receptor partial agonists for smoking cessation | 1 |
| Rigotti 2010 | Nicotine receptor partial agonists for smoking cessation | 1 |
| Rose 2013 | Nicotine receptor partial agonists for smoking cessation | 1 |
| Stein 2013 | Nicotine receptor partial agonists for smoking cessation | 1 |
| Steinberg 2011 | Nicotine receptor partial agonists for smoking cessation | 1 |
| Tashkin 2011 | Nicotine receptor partial agonists for smoking cessation | 1 |
| Tønnesen 2013 | Nicotine receptor partial agonists for smoking cessation | 1 |
| Tonstad 2006 | Nicotine receptor partial agonists for smoking cessation | 1 |
| Tsai 2007 | Nicotine receptor partial agonists for smoking cessation | 1 |
| Tsukahara 2010 | Nicotine receptor partial agonists for smoking cessation | 1 |
| Wang 2009 | Nicotine receptor partial agonists for smoking cessation | 1 |
| Westergaard 2015 | Nicotine receptor partial agonists for smoking cessation | 1 |
| Williams 2007 | Nicotine receptor partial agonists for smoking cessation | 1 |
| Wong 2012 | Nicotine receptor partial agonists for smoking cessation | 1 |
| Anserini 2008 | Pain relief in hysterosalpingography | 1 |
| Arnau 2014 | Pain relief in hysterosalpingography | 1 |
| Bachman 2014 | Pain relief in hysterosalpingography | 1 |
| Bello 2008 | Pain relief in hysterosalpingography | 1 |
| Cengiz 2006 | Pain relief in hysterosalpingography | 1 |
| Chauhan 2013 | Pain relief in hysterosalpingography | 1 |
| Costello 2002 | Pain relief in hysterosalpingography | 1 |
| de Mello 2006 | Pain relief in hysterosalpingography | 1 |
| Elson 2000 | Pain relief in hysterosalpingography | 1 |
| Frishman 2004 | Pain relief in hysterosalpingography | 1 |
| Gupta 2008 | Pain relief in hysterosalpingography | 1 |
| Hacivelioglu 2014 | Pain relief in hysterosalpingography | 1 |
| Hassa 2014 | Pain relief in hysterosalpingography | 1 |
| Jacobs 1991 | Pain relief in hysterosalpingography | 1 |
| Kafali 2003 | Pain relief in hysterosalpingography | 1 |
| Kalantari 2014 | Pain relief in hysterosalpingography | 1 |
| Karasahin 2009 | Pain relief in hysterosalpingography | 1 |
| Liberty 2007 | Pain relief in hysterosalpingography | 1 |
| Owens 1985 | Pain relief in hysterosalpingography | 1 |
| Peters 1996 | Pain relief in hysterosalpingography | 1 |
| Robinson 2007 | Pain relief in hysterosalpingography | 1 |
| Stoop 2010 | Pain relief in hysterosalpingography | 1 |
| Unlu 2015 | Pain relief in hysterosalpingography | 1 |
| Adamolekun 1999 | Care delivery and self management strategies for adults with epilepsy | 1 |
| Aliasgharpour 2013 | Care delivery and self management strategies for adults with epilepsy | 1 |
| Davis 2004 | Care delivery and self management strategies for adults with epilepsy | 1 |
| DiIorio 2011 | Care delivery and self management strategies for adults with epilepsy | 1 |
| Gilliam 2004 | Care delivery and self management strategies for adults with epilepsy | 1 |
| Helde 2005 | Care delivery and self management strategies for adults with epilepsy | 1 |
| Helgeson 1990 | Care delivery and self management strategies for adults with epilepsy | 1 |
| 37377 | Care delivery and self management strategies for adults with epilepsy | 1 |
| McAuley 2001 | Care delivery and self management strategies for adults with epilepsy | 1 |
| Mills 1999a 0000 | Care delivery and self management strategies for adults with epilepsy | 1 |
| Mills 1999b 0000 | Care delivery and self management strategies for adults with epilepsy | 1 |
| Morrow 1990 | Care delivery and self management strategies for adults with epilepsy | 1 |
| Peterson 1984 | Care delivery and self management strategies for adults with epilepsy | 1 |
| Ridsdale 1997 | Care delivery and self management strategies for adults with epilepsy | 1 |
| Ridsdale 1999 | Care delivery and self management strategies for adults with epilepsy | 1 |
| Ridsdale 2000 | Care delivery and self management strategies for adults with epilepsy | 1 |
| Thapar 2002 | Care delivery and self management strategies for adults with epilepsy | 1 |
| Warren 1998 | Care delivery and self management strategies for adults with epilepsy | 1 |
| Glueckauf 2002 | Care delivery and self management strategies for children with epilepsy | 1 |
| Jantzen 2009 | Care delivery and self management strategies for children with epilepsy | 1 |
| Lewis 1990 | Care delivery and self management strategies for children with epilepsy | 1 |
| Lewis 1991 | Care delivery and self management strategies for children with epilepsy | 1 |
| Pf[a WITH DIAERESIS]fflin 2012 | Care delivery and self management strategies for children with epilepsy | 1 |
| Rau 2006 | Care delivery and self management strategies for children with epilepsy | 1 |
| Tieffenberg 2000 | Care delivery and self management strategies for children with epilepsy | 1 |
| Katz 1995 | Mitomycin C versus 5-Fluorouracil for wound healing in glaucoma surgery | 1 |
| Kitazawa 1991 | Mitomycin C versus 5-Fluorouracil for wound healing in glaucoma surgery | 1 |
| Lamping 1995 | Mitomycin C versus 5-Fluorouracil for wound healing in glaucoma surgery | 1 |
| Mostafaei 2011 | Mitomycin C versus 5-Fluorouracil for wound healing in glaucoma surgery | 1 |
| Singh 1997 | Mitomycin C versus 5-Fluorouracil for wound healing in glaucoma surgery | 1 |
| Singh 2000 | Mitomycin C versus 5-Fluorouracil for wound healing in glaucoma surgery | 1 |
| Sisto 2007 | Mitomycin C versus 5-Fluorouracil for wound healing in glaucoma surgery | 1 |
| Uva 1996 | Mitomycin C versus 5-Fluorouracil for wound healing in glaucoma surgery | 1 |
| WuDunn 2002 | Mitomycin C versus 5-Fluorouracil for wound healing in glaucoma surgery | 1 |
| Xinyu 2001 | Mitomycin C versus 5-Fluorouracil for wound healing in glaucoma surgery | 1 |
| Zadok 1995 | Mitomycin C versus 5-Fluorouracil for wound healing in glaucoma surgery | 1 |
| Graham 1990 | Non-steroidal anti-inflammatory drugs for the common cold | 1 |
| Goto 2007 | Non-steroidal anti-inflammatory drugs for the common cold | 1 |
| Itoh 1980 | Non-steroidal anti-inflammatory drugs for the common cold | 1 |
| Katsu 1993 | Non-steroidal anti-inflammatory drugs for the common cold | 1 |
| Nagaoka 1980 | Non-steroidal anti-inflammatory drugs for the common cold | 1 |
| Ryan 1987 | Non-steroidal anti-inflammatory drugs for the common cold | 1 |
| Sperber 1989 | Non-steroidal anti-inflammatory drugs for the common cold | 1 |
| Sperber 1992 | Non-steroidal anti-inflammatory drugs for the common cold | 1 |
| Winther 2001 | Non-steroidal anti-inflammatory drugs for the common cold | 1 |
| Brooker 2002 | Chromoscopy versus conventional endoscopy for the detection of polyps in the colon and rectum | 1 |
| Hurlstone 2004 | Chromoscopy versus conventional endoscopy for the detection of polyps in the colon and rectum | 1 |
| Kahi 2010 | Chromoscopy versus conventional endoscopy for the detection of polyps in the colon and rectum | 1 |
| Lapalus 2006 | Chromoscopy versus conventional endoscopy for the detection of polyps in the colon and rectum | 1 |
| Le Rhun 2006 | Chromoscopy versus conventional endoscopy for the detection of polyps in the colon and rectum | 1 |
| Pohl 2011 | Chromoscopy versus conventional endoscopy for the detection of polyps in the colon and rectum | 1 |
| Stoffel 2008 | Chromoscopy versus conventional endoscopy for the detection of polyps in the colon and rectum | 1 |
| Angeli 2012 | Stem cell treatment for acute myocardial infarction | 1 |
| Cao 2009 | Stem cell treatment for acute myocardial infarction | 1 |
| Chen 2004 | Stem cell treatment for acute myocardial infarction | 1 |
| Colombo 2011 | Stem cell treatment for acute myocardial infarction | 1 |
| Gao 2013 | Stem cell treatment for acute myocardial infarction | 1 |
| Ge 2006 | Stem cell treatment for acute myocardial infarction | 1 |
| Grajek 2010 | Stem cell treatment for acute myocardial infarction | 1 |
| Hirsch 2011 | Stem cell treatment for acute myocardial infarction | 1 |
| Huang 2006 | Stem cell treatment for acute myocardial infarction | 1 |
| Huang 2007 | Stem cell treatment for acute myocardial infarction | 1 |
| Huikuri 2008 | Stem cell treatment for acute myocardial infarction | 1 |
| Janssens 2006 | Stem cell treatment for acute myocardial infarction | 1 |
| Jazi 2012 | Stem cell treatment for acute myocardial infarction | 1 |
| Jin 2008 | Stem cell treatment for acute myocardial infarction | 1 |
| Karpov 2005 | Stem cell treatment for acute myocardial infarction | 1 |
| Lee 2014 | Stem cell treatment for acute myocardial infarction | 1 |
| Lunde 2006 | Stem cell treatment for acute myocardial infarction | 1 |
| Meluzin 2008 | Stem cell treatment for acute myocardial infarction | 1 |
| Nogueira 2009 | Stem cell treatment for acute myocardial infarction | 1 |
| Penicka 2007 | Stem cell treatment for acute myocardial infarction | 1 |
| Piepoli 2010 | Stem cell treatment for acute myocardial infarction | 1 |
| Plewka 2009 | Stem cell treatment for acute myocardial infarction | 1 |
| Quyyumi 2011 | Stem cell treatment for acute myocardial infarction | 1 |
| Roncalli 2010 | Stem cell treatment for acute myocardial infarction | 1 |
| Ruan 2005 | Stem cell treatment for acute myocardial infarction | 1 |
| Schachinger 2006 | Stem cell treatment for acute myocardial infarction | 1 |
| Suarez de Lezo 2007 | Stem cell treatment for acute myocardial infarction | 1 |
| Sürder 2013 | Stem cell treatment for acute myocardial infarction | 1 |
| Tendera 2009 | Stem cell treatment for acute myocardial infarction | 1 |
| Traverse 2010 | Stem cell treatment for acute myocardial infarction | 1 |
| Traverse 2011 | Stem cell treatment for acute myocardial infarction | 1 |
| Traverse 2012 | Stem cell treatment for acute myocardial infarction | 1 |
| Turan 2012 | Stem cell treatment for acute myocardial infarction | 1 |
| Wang 2014 | Stem cell treatment for acute myocardial infarction | 1 |
| Wohrle 2010 | Stem cell treatment for acute myocardial infarction | 1 |
| Wollert 2004 | Stem cell treatment for acute myocardial infarction | 1 |
| Xiao 2012 | Stem cell treatment for acute myocardial infarction | 1 |
| Yao 2006 | Stem cell treatment for acute myocardial infarction | 1 |
| Yao 2009 | Stem cell treatment for acute myocardial infarction | 1 |
| You 2008 | Stem cell treatment for acute myocardial infarction | 1 |
| Zhukova 2009 | Stem cell treatment for acute myocardial infarction | 1 |
| CAPT 0000 | Laser treatment of drusen to prevent progression to advanced age-related macular degeneration | 2 |
| CNVPT 0000 | Laser treatment of drusen to prevent progression to advanced age-related macular degeneration | 2 |
| DLS 0000 | Laser treatment of drusen to prevent progression to advanced age-related macular degeneration | 2 |
| Figueroa 1994 | Laser treatment of drusen to prevent progression to advanced age-related macular degeneration | 2 |
| Frennesson 1995 | Laser treatment of drusen to prevent progression to advanced age-related macular degeneration | 2 |
| Frennesson 2009 | Laser treatment of drusen to prevent progression to advanced age-related macular degeneration | 2 |
| Laser to Drusen Study 1995 | Laser treatment of drusen to prevent progression to advanced age-related macular degeneration | 2 |
| Little 1995 | Laser treatment of drusen to prevent progression to advanced age-related macular degeneration | 2 |
| Olk 1999 | Laser treatment of drusen to prevent progression to advanced age-related macular degeneration | 2 |
| PTAMD bilateral 2009 | Laser treatment of drusen to prevent progression to advanced age-related macular degeneration | 2 |
| PTAMD unilateral 2002 | Laser treatment of drusen to prevent progression to advanced age-related macular degeneration | 2 |
| Barley 2014 | Interventions for improving outcomes in patients with multimorbidity in primary care and community settings | 1 |
| Bogner 2008 | Interventions for improving outcomes in patients with multimorbidity in primary care and community settings | 1 |
| Boult 2011 | Interventions for improving outcomes in patients with multimorbidity in primary care and community settings | 1 |
| Coventry 2015 | Interventions for improving outcomes in patients with multimorbidity in primary care and community settings | 1 |
| Eakin 2007 | Interventions for improving outcomes in patients with multimorbidity in primary care and community settings | 1 |
| Garvey 2015 | Interventions for improving outcomes in patients with multimorbidity in primary care and community settings | 1 |
| Gitlin 2009 | Interventions for improving outcomes in patients with multimorbidity in primary care and community settings | 1 |
| Hochhalter 2010 | Interventions for improving outcomes in patients with multimorbidity in primary care and community settings | 1 |
| Hogg 2009 | Interventions for improving outcomes in patients with multimorbidity in primary care and community settings | 1 |
| Katon 2010 | Interventions for improving outcomes in patients with multimorbidity in primary care and community settings | 1 |
| Kennedy 2013 | Interventions for improving outcomes in patients with multimorbidity in primary care and community settings | 1 |
| Krska 2001 | Interventions for improving outcomes in patients with multimorbidity in primary care and community settings | 1 |
| Lorig 1999 | Interventions for improving outcomes in patients with multimorbidity in primary care and community settings | 1 |
| Lynch 2014 | Interventions for improving outcomes in patients with multimorbidity in primary care and community settings | 1 |
| Martin 2013 | Interventions for improving outcomes in patients with multimorbidity in primary care and community settings | 1 |
| Morgan 2013 | Interventions for improving outcomes in patients with multimorbidity in primary care and community settings | 1 |
| Sommers 2000 | Interventions for improving outcomes in patients with multimorbidity in primary care and community settings | 1 |
| Wakefield 2012 | Interventions for improving outcomes in patients with multimorbidity in primary care and community settings | 1 |
| Agresta 2004 | Laparoscopic entry techniques | 1 |
| Akbar 2008 | Laparoscopic entry techniques | 1 |
| Angioli 1 2013 | Laparoscopic entry techniques | 1 |
| Angioli 2 2013 | Laparoscopic entry techniques | 1 |
| Angioli 3 2013 | Laparoscopic entry techniques | 1 |
| Bemelman 1 2000 | Laparoscopic entry techniques | 1 |
| Bemelman 2 2000 | Laparoscopic entry techniques | 1 |
| Bemelman 3 2000 | Laparoscopic entry techniques | 1 |
| Bhoyrul 2000 | Laparoscopic entry techniques | 1 |
| Bisgaard 2007 | Laparoscopic entry techniques | 1 |
| Borgatta 1990 | Laparoscopic entry techniques | 1 |
| Briel 2000 | Laparoscopic entry techniques | 1 |
| Byron 1993 | Laparoscopic entry techniques | 1 |
| Carter 2013 | Laparoscopic entry techniques | 1 |
| Channa 2009 | Laparoscopic entry techniques | 1 |
| Cogliandolo 1998 | Laparoscopic entry techniques | 1 |
| Cravello 1999 | Laparoscopic entry techniques | 1 |
| Deveci 2013 | Laparoscopic entry techniques | 1 |
| Feste 2000 | Laparoscopic entry techniques | 1 |
| Fonollosa 2012 | Laparoscopic entry techniques | 1 |
| Ghezzi 2005 | Laparoscopic entry techniques | 1 |
| Gunenc 2005 | Laparoscopic entry techniques | 1 |
| Hamade 2007 | Laparoscopic entry techniques | 1 |
| Huang 2012 | Laparoscopic entry techniques | 1 |
| Imran 2014 | Laparoscopic entry techniques | 1 |
| Johnson 1997 | Laparoscopic entry techniques | 1 |
| Karaca 2014 | Laparoscopic entry techniques | 1 |
| Kitano 1993 | Laparoscopic entry techniques | 1 |
| Lai 2011 | Laparoscopic entry techniques | 1 |
| Lam 2000 | Laparoscopic entry techniques | 1 |
| Luna 2013 | Laparoscopic entry techniques | 1 |
| Mettle 2000 | Laparoscopic entry techniques | 1 |
| Minervini 2008 | Laparoscopic entry techniques | 1 |
| Ostrzenski 1999 | Laparoscopic entry techniques | 1 |
| Peitgen 1997 | Laparoscopic entry techniques | 1 |
| Perez 2013 | Laparoscopic entry techniques | 1 |
| Phillips 2012 | Laparoscopic entry techniques | 1 |
| Prieto-Díaz-Chávez 2006 | Laparoscopic entry techniques | 1 |
| Santala 1999 | Laparoscopic entry techniques | 1 |
| Schulze 1999 | Laparoscopic entry techniques | 1 |
| Tansatit 1 2006 | Laparoscopic entry techniques | 1 |
| Tansatit 2 2006 | Laparoscopic entry techniques | 1 |
| Tansatit 3 2006 | Laparoscopic entry techniques | 1 |
| Tinelli 2010 | Laparoscopic entry techniques | 1 |
| Tinelli 2011 | Laparoscopic entry techniques | 1 |
| Tinelli 2013 | Laparoscopic entry techniques | 1 |
| Tsimoyiannis 2009 | Laparoscopic entry techniques | 1 |
| Venkatesh 2007 | Laparoscopic entry techniques | 1 |
| Vilallonga 2012 | Laparoscopic entry techniques | 1 |
| Villalobos 2014 | Laparoscopic entry techniques | 1 |
| Yim 2001 | Laparoscopic entry techniques | 1 |
| Zakerah 2010 | Laparoscopic entry techniques | 1 |
| Abroms 2014 | Mobile phone-based interventions for smoking cessation | 1 |
| Bock 2013 | Mobile phone-based interventions for smoking cessation | 1 |
| Borland 2013 | Mobile phone-based interventions for smoking cessation | 1 |
| Ferguson 2015 | Mobile phone-based interventions for smoking cessation | 1 |
| Free 2009 | Mobile phone-based interventions for smoking cessation | 1 |
| Free 2011 | Mobile phone-based interventions for smoking cessation | 1 |
| Gritz 2013 | Mobile phone-based interventions for smoking cessation | 1 |
| Haug 2013 | Mobile phone-based interventions for smoking cessation | 1 |
| Naughton 2014 | Mobile phone-based interventions for smoking cessation | 1 |
| Rodgers 2005 | Mobile phone-based interventions for smoking cessation | 1 |
| Shelley 2015 | Mobile phone-based interventions for smoking cessation | 1 |
| Whittaker 2011 | Mobile phone-based interventions for smoking cessation | 1 |
| Walley 2000 | Pharmaceutical policies: effects of financial incentives for prescribers | 1 |
| ORBIT 2005 | Biphasic versus monophasic waveforms for transthoracic defibrillation in out-of-hospital cardiac arrest | 1 |
| ORCA 2000 | Biphasic versus monophasic waveforms for transthoracic defibrillation in out-of-hospital cardiac arrest | 1 |
| TIMBER 2006 | Biphasic versus monophasic waveforms for transthoracic defibrillation in out-of-hospital cardiac arrest | 1 |
| van Alem 2003 | Biphasic versus monophasic waveforms for transthoracic defibrillation in out-of-hospital cardiac arrest | 1 |
| Bloom 2004 | Unfractionated or low-molecular weight heparin for induction of remission in ulcerative colitis | 1 |
| Celasco 2010 | Unfractionated or low-molecular weight heparin for induction of remission in ulcerative colitis | 1 |
| de Bievre 2007 | Unfractionated or low-molecular weight heparin for induction of remission in ulcerative colitis | 1 |
| Panes 2000 | Unfractionated or low-molecular weight heparin for induction of remission in ulcerative colitis | 1 |
| Zezos 2006 | Unfractionated or low-molecular weight heparin for induction of remission in ulcerative colitis | 1 |
| Abu-Laban 2006 | Aminophylline for bradyasystolic cardiac arrest in adults | 1 |
| Dirks 1999 | Aminophylline for bradyasystolic cardiac arrest in adults | 1 |
| Mader 1997 | Aminophylline for bradyasystolic cardiac arrest in adults | 1 |
| Mader 1999 | Aminophylline for bradyasystolic cardiac arrest in adults | 1 |
| Mader 2003 | Aminophylline for bradyasystolic cardiac arrest in adults | 1 |
| Madsen 2001 | Type I interferons for induction of remission in ulcerative colitis | 1 |
| Mannon 2010 | Type I interferons for induction of remission in ulcerative colitis | 1 |
| Musch 2005 | Type I interferons for induction of remission in ulcerative colitis | 1 |
| Nikolaus 2003 | Type I interferons for induction of remission in ulcerative colitis | 1 |
| Pena-Rossi 2008 | Type I interferons for induction of remission in ulcerative colitis | 1 |
| Tilg 2003 | Type I interferons for induction of remission in ulcerative colitis | 1 |
| Baum 1997 | High initial concentration versus low initial concentration sevoflurane for inhalational induction of anaesthesia | 1 |
| Dubois 1999 | High initial concentration versus low initial concentration sevoflurane for inhalational induction of anaesthesia | 1 |
| Epstein 1998 | High initial concentration versus low initial concentration sevoflurane for inhalational induction of anaesthesia | 1 |
| Green 2000 | High initial concentration versus low initial concentration sevoflurane for inhalational induction of anaesthesia | 1 |
| Hall 2000 | High initial concentration versus low initial concentration sevoflurane for inhalational induction of anaesthesia | 1 |
| Hsu 2000 | High initial concentration versus low initial concentration sevoflurane for inhalational induction of anaesthesia | 1 |
| Martin-Larrauri 2004 | High initial concentration versus low initial concentration sevoflurane for inhalational induction of anaesthesia | 1 |
| Mendonca 2001 | High initial concentration versus low initial concentration sevoflurane for inhalational induction of anaesthesia | 1 |
| Pancaro 2005 | High initial concentration versus low initial concentration sevoflurane for inhalational induction of anaesthesia | 1 |
| Singh 2014 | High initial concentration versus low initial concentration sevoflurane for inhalational induction of anaesthesia | 1 |
| Yurino 1995 | High initial concentration versus low initial concentration sevoflurane for inhalational induction of anaesthesia | 1 |
| Allen 1996 | Psychosocial interventions for smoking cessation in patients with coronary heart disease | 1 |
| Benner 2008 | Psychosocial interventions for smoking cessation in patients with coronary heart disease | 1 |
| Blasco 2012 | Psychosocial interventions for smoking cessation in patients with coronary heart disease | 1 |
| Bolman 2002a 0000 | Psychosocial interventions for smoking cessation in patients with coronary heart disease | 1 |
| Burt 1974 | Psychosocial interventions for smoking cessation in patients with coronary heart disease | 1 |
| Carlsson 1997 | Psychosocial interventions for smoking cessation in patients with coronary heart disease | 1 |
| CASIS 1992 | Psychosocial interventions for smoking cessation in patients with coronary heart disease | 1 |
| Chan 2012 | Psychosocial interventions for smoking cessation in patients with coronary heart disease | 1 |
| Cossette 2011 | Psychosocial interventions for smoking cessation in patients with coronary heart disease | 1 |
| Costa e Silva 2008 | Psychosocial interventions for smoking cessation in patients with coronary heart disease | 1 |
| DeBusk 1994 | Psychosocial interventions for smoking cessation in patients with coronary heart disease | 1 |
| Dornelas 2000 | Psychosocial interventions for smoking cessation in patients with coronary heart disease | 1 |
| Feeney 2001 | Psychosocial interventions for smoking cessation in patients with coronary heart disease | 1 |
| Froelicher 2004 | Psychosocial interventions for smoking cessation in patients with coronary heart disease | 1 |
| Gao 2011 | Psychosocial interventions for smoking cessation in patients with coronary heart disease | 1 |
| Hajek 2002 | Psychosocial interventions for smoking cessation in patients with coronary heart disease | 1 |
| Han 2011 | Psychosocial interventions for smoking cessation in patients with coronary heart disease | 1 |
| Hanssen 2007 | Psychosocial interventions for smoking cessation in patients with coronary heart disease | 1 |
| Hanssen 2009 | Psychosocial interventions for smoking cessation in patients with coronary heart disease | 1 |
| Heller 1993 | Psychosocial interventions for smoking cessation in patients with coronary heart disease | 1 |
| Holmes-Rovner 2008 | Psychosocial interventions for smoking cessation in patients with coronary heart disease | 1 |
| Jiang 2007 | Psychosocial interventions for smoking cessation in patients with coronary heart disease | 1 |
| Kubilius 2012 | Psychosocial interventions for smoking cessation in patients with coronary heart disease | 1 |
| Lisspers 1999 | Psychosocial interventions for smoking cessation in patients with coronary heart disease | 1 |
| Mildestvedt 2007 | Psychosocial interventions for smoking cessation in patients with coronary heart disease | 1 |
| Mitsibounas 1992 | Psychosocial interventions for smoking cessation in patients with coronary heart disease | 1 |
| Mosca 2010 | Psychosocial interventions for smoking cessation in patients with coronary heart disease | 1 |
| Naser 2008 | Psychosocial interventions for smoking cessation in patients with coronary heart disease | 1 |
| Neubeck 2011 | Psychosocial interventions for smoking cessation in patients with coronary heart disease | 1 |
| Ortigosa 2000 | Psychosocial interventions for smoking cessation in patients with coronary heart disease | 1 |
| Otterstad 2003 | Psychosocial interventions for smoking cessation in patients with coronary heart disease | 1 |
| Pedersen 2005 | Psychosocial interventions for smoking cessation in patients with coronary heart disease | 1 |
| Quist-Paulsen 2003 | Psychosocial interventions for smoking cessation in patients with coronary heart disease | 1 |
| Quist-Paulsen 2005 | Psychosocial interventions for smoking cessation in patients with coronary heart disease | 1 |
| Quist-Paulsen 2006a 0000 | Psychosocial interventions for smoking cessation in patients with coronary heart disease | 1 |
| Reid 2003 | Psychosocial interventions for smoking cessation in patients with coronary heart disease | 1 |
| Rigotti 1994 | Psychosocial interventions for smoking cessation in patients with coronary heart disease | 1 |
| Sivarajan 1983 | Psychosocial interventions for smoking cessation in patients with coronary heart disease | 1 |
| Smith 2009 | Psychosocial interventions for smoking cessation in patients with coronary heart disease | 1 |
| Taylor 1990 | Psychosocial interventions for smoking cessation in patients with coronary heart disease | 1 |
| van Elderen (group) 0000 | Psychosocial interventions for smoking cessation in patients with coronary heart disease | 1 |
| van Elderen (phone) 0000 | Psychosocial interventions for smoking cessation in patients with coronary heart disease | 1 |
| Zwisler 2008 | Psychosocial interventions for smoking cessation in patients with coronary heart disease | 1 |
| Anitua 2008 | Autologous platelet-rich plasma for treating chronic wounds | 2 |
| Driver 2006 | Autologous platelet-rich plasma for treating chronic wounds | 2 |
| Kakagia 2007 | Autologous platelet-rich plasma for treating chronic wounds | 2 |
| Knighton 1990 | Autologous platelet-rich plasma for treating chronic wounds | 2 |
| Krupski 1991 | Autologous platelet-rich plasma for treating chronic wounds | 2 |
| Li 2012 | Autologous platelet-rich plasma for treating chronic wounds | 2 |
| Planinsek Rucigaj 2007 | Autologous platelet-rich plasma for treating chronic wounds | 2 |
| Senet 2003 | Autologous platelet-rich plasma for treating chronic wounds | 2 |
| Stacey 2000 | Autologous platelet-rich plasma for treating chronic wounds | 2 |
| Weed 2004 | Autologous platelet-rich plasma for treating chronic wounds | 2 |
| Cambonie 2006 | Heliox inhalation therapy for bronchiolitis in infants | 1 |
| Chowdhury 2013 | Heliox inhalation therapy for bronchiolitis in infants | 1 |
| Hollmann 1998 | Heliox inhalation therapy for bronchiolitis in infants | 1 |
| Kim 2011 | Heliox inhalation therapy for bronchiolitis in infants | 1 |
| Kneyber 2009 | Heliox inhalation therapy for bronchiolitis in infants | 1 |
| Liet 2005 | Heliox inhalation therapy for bronchiolitis in infants | 1 |
| Martinón-Torres 2008 | Heliox inhalation therapy for bronchiolitis in infants | 1 |
| Acharya 1992 | Gonadotrophin-releasing hormone agonist protocols for pituitary suppression in assisted reproduction | 1 |
| Berker 2010 | Gonadotrophin-releasing hormone agonist protocols for pituitary suppression in assisted reproduction | 1 |
| Cedrin-Durnerin 2000 | Gonadotrophin-releasing hormone agonist protocols for pituitary suppression in assisted reproduction | 1 |
| Chatillon-Boissier 2012 | Gonadotrophin-releasing hormone agonist protocols for pituitary suppression in assisted reproduction | 1 |
| Chen 1992 | Gonadotrophin-releasing hormone agonist protocols for pituitary suppression in assisted reproduction | 1 |
| Dal Prato 2001 | Gonadotrophin-releasing hormone agonist protocols for pituitary suppression in assisted reproduction | 1 |
| De Placido 1991 | Gonadotrophin-releasing hormone agonist protocols for pituitary suppression in assisted reproduction | 1 |
| Ding 2013 | Gonadotrophin-releasing hormone agonist protocols for pituitary suppression in assisted reproduction | 1 |
| Dirnfeld 1991 | Gonadotrophin-releasing hormone agonist protocols for pituitary suppression in assisted reproduction | 1 |
| Dirnfeld 1999 | Gonadotrophin-releasing hormone agonist protocols for pituitary suppression in assisted reproduction | 1 |
| Fenichel 1988 | Gonadotrophin-releasing hormone agonist protocols for pituitary suppression in assisted reproduction | 1 |
| Foulot 1988 | Gonadotrophin-releasing hormone agonist protocols for pituitary suppression in assisted reproduction | 1 |
| Frydman 1988 | Gonadotrophin-releasing hormone agonist protocols for pituitary suppression in assisted reproduction | 1 |
| Fábregues 2005 | Gonadotrophin-releasing hormone agonist protocols for pituitary suppression in assisted reproduction | 1 |
| Garcia-Velasco 2000 | Gonadotrophin-releasing hormone agonist protocols for pituitary suppression in assisted reproduction | 1 |
| Hazout 1993 | Gonadotrophin-releasing hormone agonist protocols for pituitary suppression in assisted reproduction | 1 |
| Hedon 1988 | Gonadotrophin-releasing hormone agonist protocols for pituitary suppression in assisted reproduction | 1 |
| Isikoglu 2007 | Gonadotrophin-releasing hormone agonist protocols for pituitary suppression in assisted reproduction | 1 |
| Kingsland 1992 | Gonadotrophin-releasing hormone agonist protocols for pituitary suppression in assisted reproduction | 1 |
| Kondaveeti-Gordon 1996 | Gonadotrophin-releasing hormone agonist protocols for pituitary suppression in assisted reproduction | 1 |
| Lin 2013 | Gonadotrophin-releasing hormone agonist protocols for pituitary suppression in assisted reproduction | 1 |
| Loumaye 1989 | Gonadotrophin-releasing hormone agonist protocols for pituitary suppression in assisted reproduction | 1 |
| Pellicer 1989 | Gonadotrophin-releasing hormone agonist protocols for pituitary suppression in assisted reproduction | 1 |
| Ron-El 1990 | Gonadotrophin-releasing hormone agonist protocols for pituitary suppression in assisted reproduction | 1 |
| San Roman 1992 | Gonadotrophin-releasing hormone agonist protocols for pituitary suppression in assisted reproduction | 1 |
| Sarhan 2013 | Gonadotrophin-releasing hormone agonist protocols for pituitary suppression in assisted reproduction | 1 |
| Simon 1994 | Gonadotrophin-releasing hormone agonist protocols for pituitary suppression in assisted reproduction | 1 |
| Simons 2005 | Gonadotrophin-releasing hormone agonist protocols for pituitary suppression in assisted reproduction | 1 |
| Sunkara 2014 | Gonadotrophin-releasing hormone agonist protocols for pituitary suppression in assisted reproduction | 1 |
| Tan 1992 | Gonadotrophin-releasing hormone agonist protocols for pituitary suppression in assisted reproduction | 1 |
| Tasdemir 1995 | Gonadotrophin-releasing hormone agonist protocols for pituitary suppression in assisted reproduction | 1 |
| Urbancsek 1996 | Gonadotrophin-releasing hormone agonist protocols for pituitary suppression in assisted reproduction | 1 |
| van de-Helder 1990 | Gonadotrophin-releasing hormone agonist protocols for pituitary suppression in assisted reproduction | 1 |
| Weissman 2003 | Gonadotrophin-releasing hormone agonist protocols for pituitary suppression in assisted reproduction | 1 |
| Yang 1996 | Gonadotrophin-releasing hormone agonist protocols for pituitary suppression in assisted reproduction | 1 |
| Ye 2001 | Gonadotrophin-releasing hormone agonist protocols for pituitary suppression in assisted reproduction | 1 |
| Zhang 2009 | Gonadotrophin-releasing hormone agonist protocols for pituitary suppression in assisted reproduction | 1 |
| Aaron 2005 | Combination antimicrobial susceptibility testing for acute exacerbations in chronic infection of Pseudomonas aeruginosa in cystic fibrosis | 1 |
| Coppola 2004 | Melatonin as add-on treatment for epilepsy | 1 |
| Goldberg-Stern 2012 | Melatonin as add-on treatment for epilepsy | 1 |
| Gupta 2004a 0000 | Melatonin as add-on treatment for epilepsy | 1 |
| Gupta 2004b 0000 | Melatonin as add-on treatment for epilepsy | 1 |
| Hancock 2005 | Melatonin as add-on treatment for epilepsy | 1 |
| Jain 2015 | Melatonin as add-on treatment for epilepsy | 1 |
| Belanoff 2001 | Antiglucocorticoid and related treatments for psychosis | 1 |
| DeBattista 2006 | Antiglucocorticoid and related treatments for psychosis | 1 |
| Flores 2006 | Antiglucocorticoid and related treatments for psychosis | 1 |
| Gallagher 2005 | Antiglucocorticoid and related treatments for psychosis | 1 |
| Marco 2002 | Antiglucocorticoid and related treatments for psychosis | 1 |
| Nachshoni 2005 | Antiglucocorticoid and related treatments for psychosis | 1 |
| Newcomer 1998 | Antiglucocorticoid and related treatments for psychosis | 1 |
| Ritsner 2006 | Antiglucocorticoid and related treatments for psychosis | 1 |
| Ritsner 2010 | Antiglucocorticoid and related treatments for psychosis | 1 |
| Strous 2003 | Antiglucocorticoid and related treatments for psychosis | 1 |
| Strous 2007 | Antiglucocorticoid and related treatments for psychosis | 1 |
| Ahuja 2013 | Beta-blockers for congestive heart failure in children | 1 |
| Azeka 2002 | Beta-blockers for congestive heart failure in children | 1 |
| Buchhorn 2001 | Beta-blockers for congestive heart failure in children | 1 |
| Ghader 2009 | Beta-blockers for congestive heart failure in children | 1 |
| Huang 2013 | Beta-blockers for congestive heart failure in children | 1 |
| Ontoseno 2014 | Beta-blockers for congestive heart failure in children | 1 |
| Shaddy 2007 | Beta-blockers for congestive heart failure in children | 1 |
| Gilani 2005 | First-line chemotherapy in low-risk gestational trophoblastic neoplasia | 1 |
| Lertkhachonsuk 2009 | First-line chemotherapy in low-risk gestational trophoblastic neoplasia | 1 |
| Mousavi 2012 | First-line chemotherapy in low-risk gestational trophoblastic neoplasia | 1 |
| Osborne 2011 | First-line chemotherapy in low-risk gestational trophoblastic neoplasia | 1 |
| Shobeiri 2014 | First-line chemotherapy in low-risk gestational trophoblastic neoplasia | 1 |
| Yarandi 2008 | First-line chemotherapy in low-risk gestational trophoblastic neoplasia | 1 |
| Yarandi 2015 | First-line chemotherapy in low-risk gestational trophoblastic neoplasia | 1 |
| Abdulla 2012a 0000 | Single dose intravenous paracetamol or intravenous propacetamol for postoperative pain | 1 |
| Abdulla 2012b 0000 | Single dose intravenous paracetamol or intravenous propacetamol for postoperative pain | 1 |
| Akarsu 2010 | Single dose intravenous paracetamol or intravenous propacetamol for postoperative pain | 1 |
| Akil 2014 | Single dose intravenous paracetamol or intravenous propacetamol for postoperative pain | 1 |
| Arici 2009 | Single dose intravenous paracetamol or intravenous propacetamol for postoperative pain | 1 |
| Arslan 2011 | Single dose intravenous paracetamol or intravenous propacetamol for postoperative pain | 1 |
| Arslan 2013 | Single dose intravenous paracetamol or intravenous propacetamol for postoperative pain | 1 |
| Atef 2008 | Single dose intravenous paracetamol or intravenous propacetamol for postoperative pain | 1 |
| Aubrun 2003 | Single dose intravenous paracetamol or intravenous propacetamol for postoperative pain | 1 |
| Beaussier 2005 | Single dose intravenous paracetamol or intravenous propacetamol for postoperative pain | 1 |
| Brodner 2011 | Single dose intravenous paracetamol or intravenous propacetamol for postoperative pain | 1 |
| Cakan 2008 | Single dose intravenous paracetamol or intravenous propacetamol for postoperative pain | 1 |
| Chen 2011 | Single dose intravenous paracetamol or intravenous propacetamol for postoperative pain | 1 |
| Dejonckheere 2001 | Single dose intravenous paracetamol or intravenous propacetamol for postoperative pain | 1 |
| Delbos 1995 | Single dose intravenous paracetamol or intravenous propacetamol for postoperative pain | 1 |
| Eremenko 2008 | Single dose intravenous paracetamol or intravenous propacetamol for postoperative pain | 1 |
| Faiz 2014 | Single dose intravenous paracetamol or intravenous propacetamol for postoperative pain | 1 |
| Farkas 1992 | Single dose intravenous paracetamol or intravenous propacetamol for postoperative pain | 1 |
| Fletcher 1997 | Single dose intravenous paracetamol or intravenous propacetamol for postoperative pain | 1 |
| Hahn 2003 | Single dose intravenous paracetamol or intravenous propacetamol for postoperative pain | 1 |
| Hans 1993 | Single dose intravenous paracetamol or intravenous propacetamol for postoperative pain | 1 |
| Hiller 2004 | Single dose intravenous paracetamol or intravenous propacetamol for postoperative pain | 1 |
| Hiller 2012 | Single dose intravenous paracetamol or intravenous propacetamol for postoperative pain | 1 |
| Hynes 2006 | Single dose intravenous paracetamol or intravenous propacetamol for postoperative pain | 1 |
| Inal 2006 | Single dose intravenous paracetamol or intravenous propacetamol for postoperative pain | 1 |
| Jahr 2012 Study 2, 65+ 0000 | Single dose intravenous paracetamol or intravenous propacetamol for postoperative pain | 1 |
| Jahr 2012 Study 2, 65- 0000 | Single dose intravenous paracetamol or intravenous propacetamol for postoperative pain | 1 |
| Jahr 2012 Study 3, 65+ 0000 | Single dose intravenous paracetamol or intravenous propacetamol for postoperative pain | 1 |
| Jahr 2012 Study 3, 65- 0000 | Single dose intravenous paracetamol or intravenous propacetamol for postoperative pain | 1 |
| Jarde 1997 | Single dose intravenous paracetamol or intravenous propacetamol for postoperative pain | 1 |
| Juhl 2006 | Single dose intravenous paracetamol or intravenous propacetamol for postoperative pain | 1 |
| Kamath 2014 | Single dose intravenous paracetamol or intravenous propacetamol for postoperative pain | 1 |
| Kampe 2006 | Single dose intravenous paracetamol or intravenous propacetamol for postoperative pain | 1 |
| Kara 2010 | Single dose intravenous paracetamol or intravenous propacetamol for postoperative pain | 1 |
| Karaman 2010 | Single dose intravenous paracetamol or intravenous propacetamol for postoperative pain | 1 |
| Kemppainen 2006 | Single dose intravenous paracetamol or intravenous propacetamol for postoperative pain | 1 |
| Khajavi 2007 | Single dose intravenous paracetamol or intravenous propacetamol for postoperative pain | 1 |
| Khalili 2013 | Single dose intravenous paracetamol or intravenous propacetamol for postoperative pain | 1 |
| Khan 2007 | Single dose intravenous paracetamol or intravenous propacetamol for postoperative pain | 1 |
| Kilicaslan 2010 | Single dose intravenous paracetamol or intravenous propacetamol for postoperative pain | 1 |
| Koppert 2006 | Single dose intravenous paracetamol or intravenous propacetamol for postoperative pain | 1 |
| Korkmaz 2010 | Single dose intravenous paracetamol or intravenous propacetamol for postoperative pain | 1 |
| Lahtinen 2002 | Single dose intravenous paracetamol or intravenous propacetamol for postoperative pain | 1 |
| Landwehr 2005 | Single dose intravenous paracetamol or intravenous propacetamol for postoperative pain | 1 |
| Lee 2010 | Single dose intravenous paracetamol or intravenous propacetamol for postoperative pain | 1 |
| Leykin 2008 | Single dose intravenous paracetamol or intravenous propacetamol for postoperative pain | 1 |
| Ma 2003 | Single dose intravenous paracetamol or intravenous propacetamol for postoperative pain | 1 |
| Maghsoudi 2014 | Single dose intravenous paracetamol or intravenous propacetamol for postoperative pain | 1 |
| Marty 2005 | Single dose intravenous paracetamol or intravenous propacetamol for postoperative pain | 1 |
| Mimoz 2001 | Single dose intravenous paracetamol or intravenous propacetamol for postoperative pain | 1 |
| Mitra 2012 | Single dose intravenous paracetamol or intravenous propacetamol for postoperative pain | 1 |
| Moller 2005a 0000 | Single dose intravenous paracetamol or intravenous propacetamol for postoperative pain | 1 |
| Moller 2005b 0000 | Single dose intravenous paracetamol or intravenous propacetamol for postoperative pain | 1 |
| Mowafi 2012 | Single dose intravenous paracetamol or intravenous propacetamol for postoperative pain | 1 |
| Ohnesorge 2009 | Single dose intravenous paracetamol or intravenous propacetamol for postoperative pain | 1 |
| Omar 2011 | Single dose intravenous paracetamol or intravenous propacetamol for postoperative pain | 1 |
| Oncul 2011 | Single dose intravenous paracetamol or intravenous propacetamol for postoperative pain | 1 |
| Oreskovic 2014 | Single dose intravenous paracetamol or intravenous propacetamol for postoperative pain | 1 |
| Paech 2014 | Single dose intravenous paracetamol or intravenous propacetamol for postoperative pain | 1 |
| Peduto 1998 | Single dose intravenous paracetamol or intravenous propacetamol for postoperative pain | 1 |
| Salonen 2009 | Single dose intravenous paracetamol or intravenous propacetamol for postoperative pain | 1 |
| Sanyal 2014 | Single dose intravenous paracetamol or intravenous propacetamol for postoperative pain | 1 |
| Shimia 2014 | Single dose intravenous paracetamol or intravenous propacetamol for postoperative pain | 1 |
| Siddik 2001 | Single dose intravenous paracetamol or intravenous propacetamol for postoperative pain | 1 |
| Sinatra 2005 | Single dose intravenous paracetamol or intravenous propacetamol for postoperative pain | 1 |
| Tiippana 2008 | Single dose intravenous paracetamol or intravenous propacetamol for postoperative pain | 1 |
| Togrul 2011 | Single dose intravenous paracetamol or intravenous propacetamol for postoperative pain | 1 |
| Tunali 2013 | Single dose intravenous paracetamol or intravenous propacetamol for postoperative pain | 1 |
| Tuncel 2012 | Single dose intravenous paracetamol or intravenous propacetamol for postoperative pain | 1 |
| Unal 2013 | Single dose intravenous paracetamol or intravenous propacetamol for postoperative pain | 1 |
| Van Aken 2004 | Single dose intravenous paracetamol or intravenous propacetamol for postoperative pain | 1 |
| Varrassi 1999 | Single dose intravenous paracetamol or intravenous propacetamol for postoperative pain | 1 |
| Vuilleumier 1998 | Single dose intravenous paracetamol or intravenous propacetamol for postoperative pain | 1 |
| Wininger 2010 | Single dose intravenous paracetamol or intravenous propacetamol for postoperative pain | 1 |
| Zhou 2001 | Single dose intravenous paracetamol or intravenous propacetamol for postoperative pain | 1 |
| Berti Ceroni 2002 | Consultation liaison in primary care for people with mental disorders | 1 |
| Datto 2003 | Consultation liaison in primary care for people with mental disorders | 1 |
| De Cruppe 2005 | Consultation liaison in primary care for people with mental disorders | 1 |
| Dobscha 2006 | Consultation liaison in primary care for people with mental disorders | 1 |
| Drummond 1990 | Consultation liaison in primary care for people with mental disorders | 1 |
| Hedrick 2003 | Consultation liaison in primary care for people with mental disorders | 1 |
| Katon 1992 | Consultation liaison in primary care for people with mental disorders | 1 |
| Katon 1995 | Consultation liaison in primary care for people with mental disorders | 1 |
| Katon 1999 | Consultation liaison in primary care for people with mental disorders | 1 |
| Schrader 2005 | Consultation liaison in primary care for people with mental disorders | 1 |
| Van der Feltz 2006 | Consultation liaison in primary care for people with mental disorders | 1 |
| Worrall 1999 | Consultation liaison in primary care for people with mental disorders | 1 |
| Chan 2007 | Levonorgestrel intrauterine system for endometrial protection in women with breast cancer on adjuvant tamoxifen | 1 |
| Gardner 2000 | Levonorgestrel intrauterine system for endometrial protection in women with breast cancer on adjuvant tamoxifen | 1 |
| Kesim 2008 | Levonorgestrel intrauterine system for endometrial protection in women with breast cancer on adjuvant tamoxifen | 1 |
| Omar 2010 | Levonorgestrel intrauterine system for endometrial protection in women with breast cancer on adjuvant tamoxifen | 1 |
| Collis 2005 | Wound drainage after plastic and reconstructive surgery of the breast | 1 |
| Corion 2009 | Wound drainage after plastic and reconstructive surgery of the breast | 1 |
| Wrye 2003 | Wound drainage after plastic and reconstructive surgery of the breast | 1 |
| Mehlsen 1995 | Padma 28 for intermittent claudication | 1 |
| Sallon 1998 | Padma 28 for intermittent claudication | 1 |
| Samochowiec 1987 | Padma 28 for intermittent claudication | 1 |
| Schr[a WITH DIAERESIS]der 1985 | Padma 28 for intermittent claudication | 1 |
| Smulski 1995 | Padma 28 for intermittent claudication | 1 |
| 102-93-1 0000 | Topical NSAIDs for chronic musculoskeletal pain in adults | 1 |
| 108-97 0000 | Topical NSAIDs for chronic musculoskeletal pain in adults | 1 |
| Altman 2009 | Topical NSAIDs for chronic musculoskeletal pain in adults | 1 |
| Baer 2005 | Topical NSAIDs for chronic musculoskeletal pain in adults | 1 |
| Balthazar-Letawe 1987 | Topical NSAIDs for chronic musculoskeletal pain in adults | 1 |
| Baraf 2011 | Topical NSAIDs for chronic musculoskeletal pain in adults | 1 |
| Bolten 1991 | Topical NSAIDs for chronic musculoskeletal pain in adults | 1 |
| Bookman 2004 | Topical NSAIDs for chronic musculoskeletal pain in adults | 1 |
| Bruhlmann 2003 | Topical NSAIDs for chronic musculoskeletal pain in adults | 1 |
| Burgos 2001 | Topical NSAIDs for chronic musculoskeletal pain in adults | 1 |
| Conaghan 2013 | Topical NSAIDs for chronic musculoskeletal pain in adults | 1 |
| Dickson 1991 | Topical NSAIDs for chronic musculoskeletal pain in adults | 1 |
| Dreiser 1993 | Topical NSAIDs for chronic musculoskeletal pain in adults | 1 |
| Ergun 2007 | Topical NSAIDs for chronic musculoskeletal pain in adults | 1 |
| Galeazzi 1993 | Topical NSAIDs for chronic musculoskeletal pain in adults | 1 |
| Grace 1999 | Topical NSAIDs for chronic musculoskeletal pain in adults | 1 |
| Gui 1982 | Topical NSAIDs for chronic musculoskeletal pain in adults | 1 |
| Hohmeister 1983 | Topical NSAIDs for chronic musculoskeletal pain in adults | 1 |
| Kneer 2013 | Topical NSAIDs for chronic musculoskeletal pain in adults | 1 |
| Link 1996 | Topical NSAIDs for chronic musculoskeletal pain in adults | 1 |
| McCleane 2000 | Topical NSAIDs for chronic musculoskeletal pain in adults | 1 |
| NCT01980940 | Topical NSAIDs for chronic musculoskeletal pain in adults | 1 |
| Niethard 2005 | Topical NSAIDs for chronic musculoskeletal pain in adults | 1 |
| Ottillinger 2001 | Topical NSAIDs for chronic musculoskeletal pain in adults | 1 |
| Poul 1993 | Topical NSAIDs for chronic musculoskeletal pain in adults | 1 |
| Rose 1991 | Topical NSAIDs for chronic musculoskeletal pain in adults | 1 |
| Roth 1995 | Topical NSAIDs for chronic musculoskeletal pain in adults | 1 |
| Roth 2004 | Topical NSAIDs for chronic musculoskeletal pain in adults | 1 |
| Rother 2007 | Topical NSAIDs for chronic musculoskeletal pain in adults | 1 |
| Rother 2013 | Topical NSAIDs for chronic musculoskeletal pain in adults | 1 |
| Sandelin 1997 | Topical NSAIDs for chronic musculoskeletal pain in adults | 1 |
| Simon 2009 | Topical NSAIDs for chronic musculoskeletal pain in adults | 1 |
| Tugwell 2004 | Topical NSAIDs for chronic musculoskeletal pain in adults | 1 |
| van Haselen 2000 | Topical NSAIDs for chronic musculoskeletal pain in adults | 1 |
| Varadi 2013 | Topical NSAIDs for chronic musculoskeletal pain in adults | 1 |
| Widrig 2007 | Topical NSAIDs for chronic musculoskeletal pain in adults | 1 |
| Zacher 2001 | Topical NSAIDs for chronic musculoskeletal pain in adults | 1 |
| Digiesi 1990 | Blood pressure lowering efficacy of coenzyme Q10 for primary hypertension | 1 |
| Yamagami 1986 | Blood pressure lowering efficacy of coenzyme Q10 for primary hypertension | 1 |
| Young 2012 | Blood pressure lowering efficacy of coenzyme Q10 for primary hypertension | 1 |
| Coelho 1993 | Organising healthcare services for persons with an intellectual disability | 1 |
| Dowling 2006 | Organising healthcare services for persons with an intellectual disability | 1 |
| Hassiotis 2001 | Organising healthcare services for persons with an intellectual disability | 1 |
| Hassiotis 2009 | Organising healthcare services for persons with an intellectual disability | 1 |
| Martin 2005 | Organising healthcare services for persons with an intellectual disability | 1 |
| Oliver 2005 | Organising healthcare services for persons with an intellectual disability | 1 |
| van Minnen 1997 | Organising healthcare services for persons with an intellectual disability | 1 |
| Setcos 1999 | Adhesively bonded versus non-bonded amalgam restorations for dental caries | 1 |
| Fitzgerald 2004 | Increased versus stable doses of inhaled corticosteroids for exacerbations of chronic asthma in adults and children | 1 |
| Foresi 2000 | Increased versus stable doses of inhaled corticosteroids for exacerbations of chronic asthma in adults and children | 1 |
| Garrett 1998 | Increased versus stable doses of inhaled corticosteroids for exacerbations of chronic asthma in adults and children | 1 |
| Harrison 2004 | Increased versus stable doses of inhaled corticosteroids for exacerbations of chronic asthma in adults and children | 1 |
| Martinez 2011 | Increased versus stable doses of inhaled corticosteroids for exacerbations of chronic asthma in adults and children | 1 |
| Oborne 2009 | Increased versus stable doses of inhaled corticosteroids for exacerbations of chronic asthma in adults and children | 1 |
| Rice-McDonald 2005 | Increased versus stable doses of inhaled corticosteroids for exacerbations of chronic asthma in adults and children | 1 |
| Wainwright 2009 | Increased versus stable doses of inhaled corticosteroids for exacerbations of chronic asthma in adults and children | 1 |
| Mate-Jimenez 2000 | Methotrexate for maintenance of remission in ulcerative colitis | 1 |
| Onuk 1996 | Methotrexate for maintenance of remission in ulcerative colitis | 1 |
| Oren 1996 | Methotrexate for maintenance of remission in ulcerative colitis | 1 |
| Ackerstaff 2009 | Interventions to enhance return-to-work for cancer patients | 1 |
| Berglund 1994 | Interventions to enhance return-to-work for cancer patients | 1 |
| Burgio 2006 | Interventions to enhance return-to-work for cancer patients | 1 |
| Emmanouilidis 2009 | Interventions to enhance return-to-work for cancer patients | 1 |
| Friedrichs 2010 | Interventions to enhance return-to-work for cancer patients | 1 |
| Hillman 1998 | Interventions to enhance return-to-work for cancer patients | 1 |
| Hubbard 2013 | Interventions to enhance return-to-work for cancer patients | 1 |
| Johnsson 2007 | Interventions to enhance return-to-work for cancer patients | 1 |
| Kornblith 2009 | Interventions to enhance return-to-work for cancer patients | 1 |
| Lee 1992 | Interventions to enhance return-to-work for cancer patients | 1 |
| Lepore 2003 | Interventions to enhance return-to-work for cancer patients | 1 |
| Maguire 1983 | Interventions to enhance return-to-work for cancer patients | 1 |
| Purcell 2011 | Interventions to enhance return-to-work for cancer patients | 1 |
| Rogers 2009 | Interventions to enhance return-to-work for cancer patients | 1 |
| Tamminga 2013 | Interventions to enhance return-to-work for cancer patients | 1 |
| Kitchener 2009 | Lymphadenectomy for the management of endometrial cancer | 1 |
| Panici 2008 | Lymphadenectomy for the management of endometrial cancer | 1 |
| Ahonen 1984 | Acupuncture for the prevention of tension-type headache | 1 |
| Carlsson 1990 | Acupuncture for the prevention of tension-type headache | 1 |
| Endres 2007 | Acupuncture for the prevention of tension-type headache | 1 |
| Jena 2008 | Acupuncture for the prevention of tension-type headache | 1 |
| Karst 2001 | Acupuncture for the prevention of tension-type headache | 1 |
| Kwak 2007 | Acupuncture for the prevention of tension-type headache | 1 |
| Melchart 2005 | Acupuncture for the prevention of tension-type headache | 1 |
| Söderberg 2006 | Acupuncture for the prevention of tension-type headache | 1 |
| Tavola 1992 | Acupuncture for the prevention of tension-type headache | 1 |
| White 1996 | Acupuncture for the prevention of tension-type headache | 1 |
| White 2000 | Acupuncture for the prevention of tension-type headache | 1 |
| Wylie 1997 | Acupuncture for the prevention of tension-type headache | 1 |
| Birnbaum 2009 | Immunosuppressants for the prophylaxis of corneal graft rejection after penetrating keratoplasty | 1 |
| Javadi 2010 | Immunosuppressants for the prophylaxis of corneal graft rejection after penetrating keratoplasty | 1 |
| Reinhard 2001 | Immunosuppressants for the prophylaxis of corneal graft rejection after penetrating keratoplasty | 1 |
| Reinhard 2005 | Immunosuppressants for the prophylaxis of corneal graft rejection after penetrating keratoplasty | 1 |
| Sinha 2010 | Immunosuppressants for the prophylaxis of corneal graft rejection after penetrating keratoplasty | 1 |
| Zhang 2009 | Immunosuppressants for the prophylaxis of corneal graft rejection after penetrating keratoplasty | 1 |
| Donohoe 2000 | Complex interventions for preventing diabetic foot ulceration | 1 |
| Liang 2012 | Complex interventions for preventing diabetic foot ulceration | 1 |
| Litzelman 1993 | Complex interventions for preventing diabetic foot ulceration | 1 |
| McCabe 1998 | Complex interventions for preventing diabetic foot ulceration | 1 |
| McMurray 2002 | Complex interventions for preventing diabetic foot ulceration | 1 |
| Rönnemaa 1997 | Complex interventions for preventing diabetic foot ulceration | 1 |
| Bakris 2002 | Long-term effects of weight-reducing drugs in people with hypertension | 1 |
| Cocco 2005 | Long-term effects of weight-reducing drugs in people with hypertension | 1 |
| Guy-Grand 2004 | Long-term effects of weight-reducing drugs in people with hypertension | 1 |
| XENDOS 2001-2006 | Long-term effects of weight-reducing drugs in people with hypertension | 1 |
| Fangh[a WITH DIAERESIS]nel 2003 | Long-term effects of weight-reducing drugs in people with hypertension | 1 |
| Faria 2002 | Long-term effects of weight-reducing drugs in people with hypertension | 1 |
| McMahon 2000 | Long-term effects of weight-reducing drugs in people with hypertension | 1 |
| McMahon 2002 | Long-term effects of weight-reducing drugs in people with hypertension | 1 |
| CONQUER 2013 | Long-term effects of weight-reducing drugs in people with hypertension | 1 |
| D'Haens 2010 | Oral budesonide for induction of remission in ulcerative colitis | 1 |
| Gross 2011 | Oral budesonide for induction of remission in ulcerative colitis | 1 |
| Löfberg 1996 | Oral budesonide for induction of remission in ulcerative colitis | 1 |
| Rubin 2014 | Oral budesonide for induction of remission in ulcerative colitis | 1 |
| Sandborn 2012 | Oral budesonide for induction of remission in ulcerative colitis | 1 |
| Travis 2014 | Oral budesonide for induction of remission in ulcerative colitis | 1 |
| Farahmand 2011 | Warfarin initiation nomograms for venous thromboembolism | 1 |
| Kovacs 1998 | Warfarin initiation nomograms for venous thromboembolism | 1 |
| Kovacs 2003 | Warfarin initiation nomograms for venous thromboembolism | 1 |
| Quiroz 2006 | Warfarin initiation nomograms for venous thromboembolism | 1 |
| Gao 2003b 0000 | Ganoderma lucidum (Reishi mushroom) for cancer treatment | 1 |
| He 1997 | Ganoderma lucidum (Reishi mushroom) for cancer treatment | 1 |
| Leng 2003 | Ganoderma lucidum (Reishi mushroom) for cancer treatment | 1 |
| Yan 1998 | Ganoderma lucidum (Reishi mushroom) for cancer treatment | 1 |
| Zhang 2000 | Ganoderma lucidum (Reishi mushroom) for cancer treatment | 1 |
| Alimi 2003 | Acupuncture for cancer pain in adults | 1 |
| Chen 2008 | Acupuncture for cancer pain in adults | 1 |
| Chen 2013 | Acupuncture for cancer pain in adults | 1 |
| Dang 1998 | Acupuncture for cancer pain in adults | 1 |
| Lu 2012 | Acupuncture for cancer pain in adults | 1 |
| Alvarez 1995 | Antiemetic medication for prevention and treatment of chemotherapy-induced nausea and vomiting in childhood | 3 |
| Basade 1996 | Antiemetic medication for prevention and treatment of chemotherapy-induced nausea and vomiting in childhood | 3 |
| Berrak 2007 | Antiemetic medication for prevention and treatment of chemotherapy-induced nausea and vomiting in childhood | 3 |
| Brock 1996 | Antiemetic medication for prevention and treatment of chemotherapy-induced nausea and vomiting in childhood | 3 |
| Chan 1987 | Antiemetic medication for prevention and treatment of chemotherapy-induced nausea and vomiting in childhood | 3 |
| Dalzell 1986 | Antiemetic medication for prevention and treatment of chemotherapy-induced nausea and vomiting in childhood | 3 |
| Dick 1995 | Antiemetic medication for prevention and treatment of chemotherapy-induced nausea and vomiting in childhood | 3 |
| Ekert 1979 | Antiemetic medication for prevention and treatment of chemotherapy-induced nausea and vomiting in childhood | 3 |
| Ekert 1979a 0000 | Antiemetic medication for prevention and treatment of chemotherapy-induced nausea and vomiting in childhood | 3 |
| Emir 2013 | Antiemetic medication for prevention and treatment of chemotherapy-induced nausea and vomiting in childhood | 3 |
| Graham-Pole 1986 | Antiemetic medication for prevention and treatment of chemotherapy-induced nausea and vomiting in childhood | 3 |
| Hahlen 1995 | Antiemetic medication for prevention and treatment of chemotherapy-induced nausea and vomiting in childhood | 3 |
| Hirota 1993 | Antiemetic medication for prevention and treatment of chemotherapy-induced nausea and vomiting in childhood | 3 |
| Jaing 2004 | Antiemetic medication for prevention and treatment of chemotherapy-induced nausea and vomiting in childhood | 3 |
| Komada 1999 | Antiemetic medication for prevention and treatment of chemotherapy-induced nausea and vomiting in childhood | 3 |
| Kurucu 2012 | Antiemetic medication for prevention and treatment of chemotherapy-induced nausea and vomiting in childhood | 3 |
| Mabro 2000 | Antiemetic medication for prevention and treatment of chemotherapy-induced nausea and vomiting in childhood | 3 |
| Marshall 1989 | Antiemetic medication for prevention and treatment of chemotherapy-induced nausea and vomiting in childhood | 3 |
| Mehta 1986 | Antiemetic medication for prevention and treatment of chemotherapy-induced nausea and vomiting in childhood | 3 |
| Mehta 1997 | Antiemetic medication for prevention and treatment of chemotherapy-induced nausea and vomiting in childhood | 3 |
| Nagel 2008 | Antiemetic medication for prevention and treatment of chemotherapy-induced nausea and vomiting in childhood | 3 |
| Noguera 2001 | Antiemetic medication for prevention and treatment of chemotherapy-induced nausea and vomiting in childhood | 3 |
| Orchard 1999 | Antiemetic medication for prevention and treatment of chemotherapy-induced nausea and vomiting in childhood | 3 |
| Parker 2001 | Antiemetic medication for prevention and treatment of chemotherapy-induced nausea and vomiting in childhood | 3 |
| Safonova 1999 | Antiemetic medication for prevention and treatment of chemotherapy-induced nausea and vomiting in childhood | 3 |
| Sandoval 1999 | Antiemetic medication for prevention and treatment of chemotherapy-induced nausea and vomiting in childhood | 3 |
| Sepulveda-Vildosola 2008 | Antiemetic medication for prevention and treatment of chemotherapy-induced nausea and vomiting in childhood | 3 |
| Shi 2012 | Antiemetic medication for prevention and treatment of chemotherapy-induced nausea and vomiting in childhood | 3 |
| Siddique 2011 | Antiemetic medication for prevention and treatment of chemotherapy-induced nausea and vomiting in childhood | 3 |
| Suarez 1994 | Antiemetic medication for prevention and treatment of chemotherapy-induced nausea and vomiting in childhood | 3 |
| Swann 1979 | Antiemetic medication for prevention and treatment of chemotherapy-induced nausea and vomiting in childhood | 3 |
| Tejedor 1999 | Antiemetic medication for prevention and treatment of chemotherapy-induced nausea and vomiting in childhood | 3 |
| Tsuchida 1999 | Antiemetic medication for prevention and treatment of chemotherapy-induced nausea and vomiting in childhood | 3 |
| White 2000 | Antiemetic medication for prevention and treatment of chemotherapy-induced nausea and vomiting in childhood | 3 |
| Barker 2004 | Clinically-indicated replacement versus routine replacement of peripheral venous catheters | 1 |
| Nishanth 2009 | Clinically-indicated replacement versus routine replacement of peripheral venous catheters | 1 |
| Rickard 2010 | Clinically-indicated replacement versus routine replacement of peripheral venous catheters | 1 |
| Rickard 2012 | Clinically-indicated replacement versus routine replacement of peripheral venous catheters | 1 |
| Van Donk 2009 | Clinically-indicated replacement versus routine replacement of peripheral venous catheters | 1 |
| Webster 2007 | Clinically-indicated replacement versus routine replacement of peripheral venous catheters | 1 |
| Webster 2008 | Clinically-indicated replacement versus routine replacement of peripheral venous catheters | 1 |
| Aghamiri 2005 | Acupuncture for dysmenorrhoea | 1 |
| Bazarganipour 2010 | Acupuncture for dysmenorrhoea | 1 |
| Bu 2011 | Acupuncture for dysmenorrhoea | 1 |
| Cao 2011 | Acupuncture for dysmenorrhoea | 1 |
| Charandabi 2011 | Acupuncture for dysmenorrhoea | 1 |
| Chen 2004 | Acupuncture for dysmenorrhoea | 1 |
| Chen 2010 | Acupuncture for dysmenorrhoea | 1 |
| Darabi 2010 | Acupuncture for dysmenorrhoea | 1 |
| Han 2012 | Acupuncture for dysmenorrhoea | 1 |
| Helms 1987 | Acupuncture for dysmenorrhoea | 1 |
| Hu 2005 | Acupuncture for dysmenorrhoea | 1 |
| Kashefi 2010 | Acupuncture for dysmenorrhoea | 1 |
| Lee 2007 | Acupuncture for dysmenorrhoea | 1 |
| Li 2008 | Acupuncture for dysmenorrhoea | 1 |
| Li 2012b 0000 | Acupuncture for dysmenorrhoea | 1 |
| Ma 2010 | Acupuncture for dysmenorrhoea | 1 |
| Ma 2013 | Acupuncture for dysmenorrhoea | 1 |
| Mirbagher-Ajorpaz 2011 | Acupuncture for dysmenorrhoea | 1 |
| Peng 2012 | Acupuncture for dysmenorrhoea | 1 |
| Qiao 2013 | Acupuncture for dysmenorrhoea | 1 |
| Ruan 2011 | Acupuncture for dysmenorrhoea | 1 |
| Shi 2011 | Acupuncture for dysmenorrhoea | 1 |
| Smith 2010 | Acupuncture for dysmenorrhoea | 1 |
| Song 2013 | Acupuncture for dysmenorrhoea | 1 |
| Sriprasert 2015 | Acupuncture for dysmenorrhoea | 1 |
| Sun 2011 | Acupuncture for dysmenorrhoea | 1 |
| Wang 2013b 0000 | Acupuncture for dysmenorrhoea | 1 |
| Wang 2005b 0000 | Acupuncture for dysmenorrhoea | 1 |
| Wang 2009a 0000 | Acupuncture for dysmenorrhoea | 1 |
| Wang 2014a 0000 | Acupuncture for dysmenorrhoea | 1 |
| Witt 2008 | Acupuncture for dysmenorrhoea | 1 |
| Wong 2010 | Acupuncture for dysmenorrhoea | 1 |
| Xu 2013 | Acupuncture for dysmenorrhoea | 1 |
| Xu 2014 | Acupuncture for dysmenorrhoea | 1 |
| Yeh 2013a 0000 | Acupuncture for dysmenorrhoea | 1 |
| Youn 2008 | Acupuncture for dysmenorrhoea | 1 |
| Yu 2014 | Acupuncture for dysmenorrhoea | 1 |
| Zafari 2011 | Acupuncture for dysmenorrhoea | 1 |
| Zhang 2012 | Acupuncture for dysmenorrhoea | 1 |
| Zhang 2013a 0000 | Acupuncture for dysmenorrhoea | 1 |
| Zhang 2013b 0000 | Acupuncture for dysmenorrhoea | 1 |
| Zhi 2007 | Acupuncture for dysmenorrhoea | 1 |
| Arenas 2007 | Culture media for human pre-implantation embryos in assisted reproductive technology cycles | 1 |
| Artini 2004 | Culture media for human pre-implantation embryos in assisted reproductive technology cycles | 1 |
| Barak 1998 | Culture media for human pre-implantation embryos in assisted reproductive technology cycles | 1 |
| Baum 2004 | Culture media for human pre-implantation embryos in assisted reproductive technology cycles | 1 |
| Bird 2012 | Culture media for human pre-implantation embryos in assisted reproductive technology cycles | 1 |
| Bungum 2003 | Culture media for human pre-implantation embryos in assisted reproductive technology cycles | 1 |
| Campo 2010 | Culture media for human pre-implantation embryos in assisted reproductive technology cycles | 1 |
| Cano 2001 | Culture media for human pre-implantation embryos in assisted reproductive technology cycles | 1 |
| Carrasco 2013 | Culture media for human pre-implantation embryos in assisted reproductive technology cycles | 1 |
| Chatziioannou 2010 | Culture media for human pre-implantation embryos in assisted reproductive technology cycles | 1 |
| Chen 2009 | Culture media for human pre-implantation embryos in assisted reproductive technology cycles | 1 |
| Fechtali 2004 | Culture media for human pre-implantation embryos in assisted reproductive technology cycles | 1 |
| Findikli 2004 | Culture media for human pre-implantation embryos in assisted reproductive technology cycles | 1 |
| Gimeno 2006 | Culture media for human pre-implantation embryos in assisted reproductive technology cycles | 1 |
| Hazlett 2003 | Culture media for human pre-implantation embryos in assisted reproductive technology cycles | 1 |
| Jamieson 1997 | Culture media for human pre-implantation embryos in assisted reproductive technology cycles | 1 |
| Khan 2004 | Culture media for human pre-implantation embryos in assisted reproductive technology cycles | 1 |
| Kyono 2000 | Culture media for human pre-implantation embryos in assisted reproductive technology cycles | 1 |
| Lambert 2005 | Culture media for human pre-implantation embryos in assisted reproductive technology cycles | 1 |
| Mauri 2001 | Culture media for human pre-implantation embryos in assisted reproductive technology cycles | 1 |
| Mayer 2003 | Culture media for human pre-implantation embryos in assisted reproductive technology cycles | 1 |
| Mendoza 2003 | Culture media for human pre-implantation embryos in assisted reproductive technology cycles | 1 |
| Paternot 2010 | Culture media for human pre-implantation embryos in assisted reproductive technology cycles | 1 |
| Rubino 2004 | Culture media for human pre-implantation embryos in assisted reproductive technology cycles | 1 |
| Sepulveda 2009 | Culture media for human pre-implantation embryos in assisted reproductive technology cycles | 1 |
| Shih 2014 | Culture media for human pre-implantation embryos in assisted reproductive technology cycles | 1 |
| Sifer 2009 | Culture media for human pre-implantation embryos in assisted reproductive technology cycles | 1 |
| Stevens 2000 | Culture media for human pre-implantation embryos in assisted reproductive technology cycles | 1 |
| Tedesco 1990 | Culture media for human pre-implantation embryos in assisted reproductive technology cycles | 1 |
| Von During 2004 | Culture media for human pre-implantation embryos in assisted reproductive technology cycles | 1 |
| Yamamoto 2006 | Culture media for human pre-implantation embryos in assisted reproductive technology cycles | 1 |
| Zollner 2004 | Culture media for human pre-implantation embryos in assisted reproductive technology cycles | 1 |
| Alam 2012 | Drug therapy for treating post-dural puncture headache | 1 |
| Camann 1990 | Drug therapy for treating post-dural puncture headache | 1 |
| Connelly 2000 | Drug therapy for treating post-dural puncture headache | 1 |
| Dogan 2006 | Drug therapy for treating post-dural puncture headache | 1 |
| Erol 2011 | Drug therapy for treating post-dural puncture headache | 1 |
| Feuerstein 1986 | Drug therapy for treating post-dural puncture headache | 1 |
| Huseyinoglu 2011 | Drug therapy for treating post-dural puncture headache | 1 |
| Mahoori 2013 | Drug therapy for treating post-dural puncture headache | 1 |
| Noyan 2007 | Drug therapy for treating post-dural puncture headache | 1 |
| Rucklidge 2004 | Drug therapy for treating post-dural puncture headache | 1 |
| Sechzer 1978 | Drug therapy for treating post-dural puncture headache | 1 |
| Sen 2014 | Drug therapy for treating post-dural puncture headache | 1 |
| Zeger 2012 | Drug therapy for treating post-dural puncture headache | 1 |
| Mathiesen 2007 | Medical interventions for high-grade vulval intraepithelial neoplasia | 1 |
| Naik 2006 | Medical interventions for high-grade vulval intraepithelial neoplasia | 1 |
| Sterling 2005 | Medical interventions for high-grade vulval intraepithelial neoplasia | 1 |
| Tristram 2014 | Medical interventions for high-grade vulval intraepithelial neoplasia | 1 |
| Van Seters 2008 | Medical interventions for high-grade vulval intraepithelial neoplasia | 1 |
| Akpinarli 1999 | Addition of long-acting beta2-agonists to inhaled corticosteroids for chronic asthma in children | 1 |
| Berger 2010 | Addition of long-acting beta2-agonists to inhaled corticosteroids for chronic asthma in children | 1 |
| Bisgaard 2006 | Addition of long-acting beta2-agonists to inhaled corticosteroids for chronic asthma in children | 1 |
| Carroll 2010 | Addition of long-acting beta2-agonists to inhaled corticosteroids for chronic asthma in children | 1 |
| De Blic 2009 | Addition of long-acting beta2-agonists to inhaled corticosteroids for chronic asthma in children | 1 |
| Eid 2010a 0000 | Addition of long-acting beta2-agonists to inhaled corticosteroids for chronic asthma in children | 1 |
| Eid 2010b 0000 | Addition of long-acting beta2-agonists to inhaled corticosteroids for chronic asthma in children | 1 |
| Gappa 2009 | Addition of long-acting beta2-agonists to inhaled corticosteroids for chronic asthma in children | 1 |
| Heuck 2000 | Addition of long-acting beta2-agonists to inhaled corticosteroids for chronic asthma in children | 1 |
| Langton Hewer 1995 | Addition of long-acting beta2-agonists to inhaled corticosteroids for chronic asthma in children | 1 |
| Lemanske 2010 | Addition of long-acting beta2-agonists to inhaled corticosteroids for chronic asthma in children | 1 |
| Lenney 2013 | Addition of long-acting beta2-agonists to inhaled corticosteroids for chronic asthma in children | 1 |
| Malone 2005 | Addition of long-acting beta2-agonists to inhaled corticosteroids for chronic asthma in children | 1 |
| Meijer 1995 | Addition of long-acting beta2-agonists to inhaled corticosteroids for chronic asthma in children | 1 |
| Morice 2008a 0000 | Addition of long-acting beta2-agonists to inhaled corticosteroids for chronic asthma in children | 1 |
| Morice 2008b 0000 | Addition of long-acting beta2-agonists to inhaled corticosteroids for chronic asthma in children | 1 |
| Murray 2010 | Addition of long-acting beta2-agonists to inhaled corticosteroids for chronic asthma in children | 1 |
| Murray 2011 | Addition of long-acting beta2-agonists to inhaled corticosteroids for chronic asthma in children | 1 |
| Ortega-Cisneros 1998 | Addition of long-acting beta2-agonists to inhaled corticosteroids for chronic asthma in children | 1 |
| Pearlman 2009 | Addition of long-acting beta2-agonists to inhaled corticosteroids for chronic asthma in children | 1 |
| Pohunek 2006a 0000 | Addition of long-acting beta2-agonists to inhaled corticosteroids for chronic asthma in children | 1 |
| Pohunek 2006b 0000 | Addition of long-acting beta2-agonists to inhaled corticosteroids for chronic asthma in children | 1 |
| Russell 1995 | Addition of long-acting beta2-agonists to inhaled corticosteroids for chronic asthma in children | 1 |
| Rutkowski 2009 | Addition of long-acting beta2-agonists to inhaled corticosteroids for chronic asthma in children | 1 |
| SAM40012a 0000 | Addition of long-acting beta2-agonists to inhaled corticosteroids for chronic asthma in children | 1 |
| SAM40012b 0000 | Addition of long-acting beta2-agonists to inhaled corticosteroids for chronic asthma in children | 1 |
| SAM40100 | Addition of long-acting beta2-agonists to inhaled corticosteroids for chronic asthma in children | 1 |
| SD 039 0714 | Addition of long-acting beta2-agonists to inhaled corticosteroids for chronic asthma in children | 1 |
| SD 039 0718 | Addition of long-acting beta2-agonists to inhaled corticosteroids for chronic asthma in children | 1 |
| Simons 1997 | Addition of long-acting beta2-agonists to inhaled corticosteroids for chronic asthma in children | 1 |
| Stelmach 2007 | Addition of long-acting beta2-agonists to inhaled corticosteroids for chronic asthma in children | 1 |
| Stelmach 2008 | Addition of long-acting beta2-agonists to inhaled corticosteroids for chronic asthma in children | 1 |
| Tal 2002 | Addition of long-acting beta2-agonists to inhaled corticosteroids for chronic asthma in children | 1 |
| Teper 2005 | Addition of long-acting beta2-agonists to inhaled corticosteroids for chronic asthma in children | 1 |
| Vaessen-Verberne 2010 | Addition of long-acting beta2-agonists to inhaled corticosteroids for chronic asthma in children | 1 |
| Verberne 1998a 0000 | Addition of long-acting beta2-agonists to inhaled corticosteroids for chronic asthma in children | 1 |
| Verberne 1998b 0000 | Addition of long-acting beta2-agonists to inhaled corticosteroids for chronic asthma in children | 1 |
| Zimmerman 2004a 0000 | Addition of long-acting beta2-agonists to inhaled corticosteroids for chronic asthma in children | 1 |
| Zimmerman 2004b 0000 | Addition of long-acting beta2-agonists to inhaled corticosteroids for chronic asthma in children | 1 |
| Chaisson 2001 | Incentives and enablers to improve adherence in tuberculosis | 1 |
| Lutge 2013 | Incentives and enablers to improve adherence in tuberculosis | 1 |
| Malotte 1998 | Incentives and enablers to improve adherence in tuberculosis | 1 |
| Malotte 1999 | Incentives and enablers to improve adherence in tuberculosis | 1 |
| Malotte 2001 | Incentives and enablers to improve adherence in tuberculosis | 1 |
| Martins 2009 | Incentives and enablers to improve adherence in tuberculosis | 1 |
| Morisky 2001 | Incentives and enablers to improve adherence in tuberculosis | 1 |
| Pilote 1996 | Incentives and enablers to improve adherence in tuberculosis | 1 |
| Tulsky 2000 | Incentives and enablers to improve adherence in tuberculosis | 1 |
| Tulsky 2004 | Incentives and enablers to improve adherence in tuberculosis | 1 |
| White 1998 | Incentives and enablers to improve adherence in tuberculosis | 1 |
| White 2002 | Incentives and enablers to improve adherence in tuberculosis | 1 |
| Puhakka 1998 | Corticosteroids for the common cold | 1 |
| Qvarnberg 2001 | Corticosteroids for the common cold | 1 |
| Rahmati 2013 | Corticosteroids for the common cold | 1 |
| Muenzer 2006 | Enzyme replacement therapy with idursulfase for mucopolysaccharidosis type II (Hunter syndrome) | 1 |
| Anon 2000 | Amitriptyline for neuropathic pain in adults | 1 |
| Biesbroeck 1995 | Amitriptyline for neuropathic pain in adults | 1 |
| Boyle 2012 | Amitriptyline for neuropathic pain in adults | 1 |
| Cardenas 2002 | Amitriptyline for neuropathic pain in adults | 1 |
| Graff-Radford 2000 | Amitriptyline for neuropathic pain in adults | 1 |
| Jose 2007 | Amitriptyline for neuropathic pain in adults | 1 |
| Kautio 2008 | Amitriptyline for neuropathic pain in adults | 1 |
| Leijon 1989 | Amitriptyline for neuropathic pain in adults | 1 |
| Max 1988 | Amitriptyline for neuropathic pain in adults | 1 |
| Max 1992 | Amitriptyline for neuropathic pain in adults | 1 |
| Mishra 2012 | Amitriptyline for neuropathic pain in adults | 1 |
| Rintala 2007 | Amitriptyline for neuropathic pain in adults | 1 |
| Rowbotham 2005 | Amitriptyline for neuropathic pain in adults | 1 |
| Shlay 1998 | Amitriptyline for neuropathic pain in adults | 1 |
| Vrethem 1997 | Amitriptyline for neuropathic pain in adults | 1 |
| Watson 1992 | Amitriptyline for neuropathic pain in adults | 1 |
| Watson 1998 | Amitriptyline for neuropathic pain in adults | 1 |
| Cohen 1991 | Long-term effects of weight-reducing diets in people with hypertension | 1 |
| Croft 1986 | Long-term effects of weight-reducing diets in people with hypertension | 1 |
| DISH 1985 | Long-term effects of weight-reducing diets in people with hypertension | 1 |
| Jalkanen 1991 | Long-term effects of weight-reducing diets in people with hypertension | 1 |
| ODES 1995 | Long-term effects of weight-reducing diets in people with hypertension | 1 |
| Ruvolo 1994 | Long-term effects of weight-reducing diets in people with hypertension | 1 |
| TAIM 1992 | Long-term effects of weight-reducing diets in people with hypertension | 1 |
| TONE 1998 | Long-term effects of weight-reducing diets in people with hypertension | 1 |
| Alten 2011 | Biologic interventions for fatigue in rheumatoid arthritis | 1 |
| Bae 2013 | Biologic interventions for fatigue in rheumatoid arthritis | 1 |
| Choy 2012 | Biologic interventions for fatigue in rheumatoid arthritis | 1 |
| Cohen 2006 | Biologic interventions for fatigue in rheumatoid arthritis | 1 |
| Emery 2006 | Biologic interventions for fatigue in rheumatoid arthritis | 1 |
| Emery 2008 | Biologic interventions for fatigue in rheumatoid arthritis | 1 |
| Emery 2009 | Biologic interventions for fatigue in rheumatoid arthritis | 1 |
| Fleischmann 2009 | Biologic interventions for fatigue in rheumatoid arthritis | 1 |
| Genovese 2005 | Biologic interventions for fatigue in rheumatoid arthritis | 1 |
| Genovese 2008 | Biologic interventions for fatigue in rheumatoid arthritis | 1 |
| Hørslev-Petersen 2014 | Biologic interventions for fatigue in rheumatoid arthritis | 1 |
| Keystone 2004 | Biologic interventions for fatigue in rheumatoid arthritis | 1 |
| Keystone 2009 | Biologic interventions for fatigue in rheumatoid arthritis | 1 |
| Kremer 2003 | Biologic interventions for fatigue in rheumatoid arthritis | 1 |
| Kremer 2006 | Biologic interventions for fatigue in rheumatoid arthritis | 1 |
| Li 2013 | Biologic interventions for fatigue in rheumatoid arthritis | 1 |
| Lukina 1998 | Biologic interventions for fatigue in rheumatoid arthritis | 1 |
| Maini 1999 | Biologic interventions for fatigue in rheumatoid arthritis | 1 |
| Mittendorf 2007 | Biologic interventions for fatigue in rheumatoid arthritis | 1 |
| Moreland 1999 | Biologic interventions for fatigue in rheumatoid arthritis | 1 |
| Pope 2012 | Biologic interventions for fatigue in rheumatoid arthritis | 1 |
| Rigby 2011 | Biologic interventions for fatigue in rheumatoid arthritis | 1 |
| Schiff 2008 | Biologic interventions for fatigue in rheumatoid arthritis | 1 |
| Smolen 2008 | Biologic interventions for fatigue in rheumatoid arthritis | 1 |
| Smolen 2009a 0000 | Biologic interventions for fatigue in rheumatoid arthritis | 1 |
| Smolen 2009b 0000 | Biologic interventions for fatigue in rheumatoid arthritis | 1 |
| Soubrier 2009 | Biologic interventions for fatigue in rheumatoid arthritis | 1 |
| Strand 2009 | Biologic interventions for fatigue in rheumatoid arthritis | 1 |
| Strand 2012a 0000 | Biologic interventions for fatigue in rheumatoid arthritis | 1 |
| Strand 2012b 0000 | Biologic interventions for fatigue in rheumatoid arthritis | 1 |
| Weinblatt 2003 | Biologic interventions for fatigue in rheumatoid arthritis | 1 |
| Weinblatt 2013 | Biologic interventions for fatigue in rheumatoid arthritis | 1 |
| Chouhan 2006 | Intraoperative mild hypothermia for postoperative neurological deficits in people with intracranial aneurysm | 1 |
| Hindman 1999 | Intraoperative mild hypothermia for postoperative neurological deficits in people with intracranial aneurysm | 1 |
| Todd 2005 | Intraoperative mild hypothermia for postoperative neurological deficits in people with intracranial aneurysm | 1 |
| Aaron 2007 | Combination inhaled steroid and long-acting beta2-agonist in addition to tiotropium versus tiotropium or combination alone for chronic obstructive pulmonary disease | 1 |
| Cazzola 2007 | Combination inhaled steroid and long-acting beta2-agonist in addition to tiotropium versus tiotropium or combination alone for chronic obstructive pulmonary disease | 1 |
| Hanania 2011 | Combination inhaled steroid and long-acting beta2-agonist in addition to tiotropium versus tiotropium or combination alone for chronic obstructive pulmonary disease | 1 |
| Hoshino 2011 | Combination inhaled steroid and long-acting beta2-agonist in addition to tiotropium versus tiotropium or combination alone for chronic obstructive pulmonary disease | 1 |
| Jung 2012 | Combination inhaled steroid and long-acting beta2-agonist in addition to tiotropium versus tiotropium or combination alone for chronic obstructive pulmonary disease | 1 |
| Welte 2009 | Combination inhaled steroid and long-acting beta2-agonist in addition to tiotropium versus tiotropium or combination alone for chronic obstructive pulmonary disease | 1 |
| Brandes 2007 Study 1 0000 | Sumatriptan plus naproxen for the treatment of acute migraine attacks in adults | 1 |
| Brandes 2007 Study 2 0000 | Sumatriptan plus naproxen for the treatment of acute migraine attacks in adults | 1 |
| Calhoun 2014 | Sumatriptan plus naproxen for the treatment of acute migraine attacks in adults | 1 |
| Lipton 2009 Study 1 0000 | Sumatriptan plus naproxen for the treatment of acute migraine attacks in adults | 1 |
| Lipton 2009 Study 2 0000 | Sumatriptan plus naproxen for the treatment of acute migraine attacks in adults | 1 |
| Mannix 2009 Study 1 0000 | Sumatriptan plus naproxen for the treatment of acute migraine attacks in adults | 1 |
| Mannix 2009 Study 2 0000 | Sumatriptan plus naproxen for the treatment of acute migraine attacks in adults | 1 |
| Mathew 2009 Study 1 0000 | Sumatriptan plus naproxen for the treatment of acute migraine attacks in adults | 1 |
| Mathew 2009 Study 2 0000 | Sumatriptan plus naproxen for the treatment of acute migraine attacks in adults | 1 |
| Silberstein 2008 Study 1 0000 | Sumatriptan plus naproxen for the treatment of acute migraine attacks in adults | 1 |
| Silberstein 2008 Study 2 0000 | Sumatriptan plus naproxen for the treatment of acute migraine attacks in adults | 1 |
| Smith 2005 | Sumatriptan plus naproxen for the treatment of acute migraine attacks in adults | 1 |
| TRX109011/13 0000 | Sumatriptan plus naproxen for the treatment of acute migraine attacks in adults | 1 |
| Alvarez 2012 | Debridement for venous leg ulcers | 4 |
| Gethin 2007 | Debridement for venous leg ulcers | 4 |
| Groenewald 1980 | Debridement for venous leg ulcers | 4 |
| Hansson 1998 | Debridement for venous leg ulcers | 4 |
| Jasiel 1996 | Debridement for venous leg ulcers | 4 |
| Konig 2005 | Debridement for venous leg ulcers | 4 |
| Skog 1983 | Debridement for venous leg ulcers | 4 |
| Wayman 2000 | Debridement for venous leg ulcers | 4 |
| Westerhof 1990 | Debridement for venous leg ulcers | 4 |
| Wild 2010 | Debridement for venous leg ulcers | 4 |
| Carr 2014 | Memory rehabilitation for people with multiple sclerosis | 1 |
| Chiaravalloti 2005 | Memory rehabilitation for people with multiple sclerosis | 1 |
| Chiaravalloti 2013 | Memory rehabilitation for people with multiple sclerosis | 1 |
| das Nair 2012 | Memory rehabilitation for people with multiple sclerosis | 1 |
| Gich 2015 | Memory rehabilitation for people with multiple sclerosis | 1 |
| Hancock 2015 | Memory rehabilitation for people with multiple sclerosis | 1 |
| Hanssen 2015 | Memory rehabilitation for people with multiple sclerosis | 1 |
| Hildebrandt 2007 | Memory rehabilitation for people with multiple sclerosis | 1 |
| Jønsson 1993 | Memory rehabilitation for people with multiple sclerosis | 1 |
| Lincoln 2002 | Memory rehabilitation for people with multiple sclerosis | 1 |
| Mendozzi 1998 | Memory rehabilitation for people with multiple sclerosis | 1 |
| Pusswald 2014 | Memory rehabilitation for people with multiple sclerosis | 1 |
| Solari 2004 | Memory rehabilitation for people with multiple sclerosis | 1 |
| Stuifbergen 2012 | Memory rehabilitation for people with multiple sclerosis | 1 |
| Tesar 2005 | Memory rehabilitation for people with multiple sclerosis | 1 |
| Berger 1998 | Vaccines for preventing herpes zoster in older adults | 1 |
| Chlibek 2013 | Vaccines for preventing herpes zoster in older adults | 1 |
| Chlibek 2014 | Vaccines for preventing herpes zoster in older adults | 1 |
| Diez-Domingo 2015 | Vaccines for preventing herpes zoster in older adults | 1 |
| Gilderman 2008 | Vaccines for preventing herpes zoster in older adults | 1 |
| Lal 2015 | Vaccines for preventing herpes zoster in older adults | 1 |
| Levin 2000 | Vaccines for preventing herpes zoster in older adults | 1 |
| Mills 2010 | Vaccines for preventing herpes zoster in older adults | 1 |
| Murray 2011 | Vaccines for preventing herpes zoster in older adults | 1 |
| Oxman 2005 | Vaccines for preventing herpes zoster in older adults | 1 |
| Tyring 2007 | Vaccines for preventing herpes zoster in older adults | 1 |
| Vermeulen 2012 | Vaccines for preventing herpes zoster in older adults | 1 |
| Vesikari 2013 | Vaccines for preventing herpes zoster in older adults | 1 |
| Crohn's III 1997 | Aminosalicylates for induction of remission or response in Crohn's disease | 1 |
| Gross 1995 | Aminosalicylates for induction of remission or response in Crohn's disease | 1 |
| Mahida 1990 | Aminosalicylates for induction of remission or response in Crohn's disease | 1 |
| Maier 1985 | Aminosalicylates for induction of remission or response in Crohn's disease | 1 |
| Maier 1990 | Aminosalicylates for induction of remission or response in Crohn's disease | 1 |
| Malchow 1984 | Aminosalicylates for induction of remission or response in Crohn's disease | 1 |
| Martin 1990 | Aminosalicylates for induction of remission or response in Crohn's disease | 1 |
| Prantera 1999 | Aminosalicylates for induction of remission or response in Crohn's disease | 1 |
| Rasmussen 1987 | Aminosalicylates for induction of remission or response in Crohn's disease | 1 |
| Rijk 1991 | Aminosalicylates for induction of remission or response in Crohn's disease | 1 |
| Saverymuttu 1986 | Aminosalicylates for induction of remission or response in Crohn's disease | 1 |
| Scholmerich 1990 | Aminosalicylates for induction of remission or response in Crohn's disease | 1 |
| Singleton 1993 | Aminosalicylates for induction of remission or response in Crohn's disease | 1 |
| Singleton 1994 | Aminosalicylates for induction of remission or response in Crohn's disease | 1 |
| Summers 1979 | Aminosalicylates for induction of remission or response in Crohn's disease | 1 |
| Thomsen 1998 | Aminosalicylates for induction of remission or response in Crohn's disease | 1 |
| Tremaine 1994 | Aminosalicylates for induction of remission or response in Crohn's disease | 1 |
| Tromm 2011 | Aminosalicylates for induction of remission or response in Crohn's disease | 1 |
| Van Hees 1981 | Aminosalicylates for induction of remission or response in Crohn's disease | 1 |
| Wright 1995 | Aminosalicylates for induction of remission or response in Crohn's disease | 1 |
| Kamoda 2008 | Stents for the prevention of pancreatic fistula following pancreaticoduodenectomy | 1 |
| Motoi 2012 | Stents for the prevention of pancreatic fistula following pancreaticoduodenectomy | 1 |
| Pessaux 2011 | Stents for the prevention of pancreatic fistula following pancreaticoduodenectomy | 1 |
| Poon 2007 | Stents for the prevention of pancreatic fistula following pancreaticoduodenectomy | 1 |
| Tani 2010 | Stents for the prevention of pancreatic fistula following pancreaticoduodenectomy | 1 |
| Wang 2014 | Stents for the prevention of pancreatic fistula following pancreaticoduodenectomy | 1 |
| Winter 2006 | Stents for the prevention of pancreatic fistula following pancreaticoduodenectomy | 1 |
| Yokoyama 2014 | Stents for the prevention of pancreatic fistula following pancreaticoduodenectomy | 1 |
| Brumley 2007 | Hospital at home: home-based end-of-life care | 1 |
| Grande 2000 | Hospital at home: home-based end-of-life care | 1 |
| Hughes 1992 | Hospital at home: home-based end-of-life care | 1 |
| Jordhøy 2000 | Hospital at home: home-based end-of-life care | 1 |
| Jia 2009a0000 | Periodontal therapy as adjunctive treatment for gastric Helicobacter pylori infection | 1 |
| Jin 2003 | Periodontal therapy as adjunctive treatment for gastric Helicobacter pylori infection | 1 |
| Jin 2007 | Periodontal therapy as adjunctive treatment for gastric Helicobacter pylori infection | 1 |
| Liu 2012 | Periodontal therapy as adjunctive treatment for gastric Helicobacter pylori infection | 1 |
| Lv 2006 | Periodontal therapy as adjunctive treatment for gastric Helicobacter pylori infection | 1 |
| Wang 2014 | Periodontal therapy as adjunctive treatment for gastric Helicobacter pylori infection | 1 |
| Zaric 2009 | Periodontal therapy as adjunctive treatment for gastric Helicobacter pylori infection | 1 |
| Auerbach 2004a 0000 | The role of iron in the management of chemotherapy-induced anemia in cancer patients receiving erythropoiesis-stimulating agents | 1 |
| Auerbach 2004b 0000 | The role of iron in the management of chemotherapy-induced anemia in cancer patients receiving erythropoiesis-stimulating agents | 1 |
| Auerbach 2004c 0000 | The role of iron in the management of chemotherapy-induced anemia in cancer patients receiving erythropoiesis-stimulating agents | 1 |
| Auerbach 2010 | The role of iron in the management of chemotherapy-induced anemia in cancer patients receiving erythropoiesis-stimulating agents | 1 |
| Bastit 2008 | The role of iron in the management of chemotherapy-induced anemia in cancer patients receiving erythropoiesis-stimulating agents | 1 |
| Beguin 2008 | The role of iron in the management of chemotherapy-induced anemia in cancer patients receiving erythropoiesis-stimulating agents | 1 |
| Bellet 2007 | The role of iron in the management of chemotherapy-induced anemia in cancer patients receiving erythropoiesis-stimulating agents | 1 |
| Henry 2007a 0000 | The role of iron in the management of chemotherapy-induced anemia in cancer patients receiving erythropoiesis-stimulating agents | 1 |
| Henry 2007b 0000 | The role of iron in the management of chemotherapy-induced anemia in cancer patients receiving erythropoiesis-stimulating agents | 1 |
| Pedrazzoli 2008 | The role of iron in the management of chemotherapy-induced anemia in cancer patients receiving erythropoiesis-stimulating agents | 1 |
| Steensma 2011a 0000 | The role of iron in the management of chemotherapy-induced anemia in cancer patients receiving erythropoiesis-stimulating agents | 1 |
| Steensma 2011b 0000 | The role of iron in the management of chemotherapy-induced anemia in cancer patients receiving erythropoiesis-stimulating agents | 1 |
| Godfrey 2010 | Hormonal and intrauterine methods for contraception for women aged 25 years and younger | 1 |
| Kaunitz 2013 | Hormonal and intrauterine methods for contraception for women aged 25 years and younger | 1 |
| Stewart 2007 | Hormonal and intrauterine methods for contraception for women aged 25 years and younger | 1 |
| Stuart 2005 | Hormonal and intrauterine methods for contraception for women aged 25 years and younger | 1 |
| Suhonen 2004 | Hormonal and intrauterine methods for contraception for women aged 25 years and younger | 1 |
| Barcelo 1993 | Citrate salts for preventing and treating calcium containing kidney stones in adults | 1 |
| Cicerello 1994 | Citrate salts for preventing and treating calcium containing kidney stones in adults | 1 |
| Ettinger 1997 | Citrate salts for preventing and treating calcium containing kidney stones in adults | 1 |
| Hofbauer 1994 | Citrate salts for preventing and treating calcium containing kidney stones in adults | 1 |
| Jimenez Verdejo 2001 | Citrate salts for preventing and treating calcium containing kidney stones in adults | 1 |
| Lojanapiwat 2011 | Citrate salts for preventing and treating calcium containing kidney stones in adults | 1 |
| Soygur 2002 | Citrate salts for preventing and treating calcium containing kidney stones in adults | 1 |
| Amundsen 2000 | Surgical versus non-surgical treatment for lumbar spinal stenosis | 1 |
| Brown 2012 | Surgical versus non-surgical treatment for lumbar spinal stenosis | 1 |
| Malmivaara 2007 | Surgical versus non-surgical treatment for lumbar spinal stenosis | 1 |
| Weinstein 2008 | Surgical versus non-surgical treatment for lumbar spinal stenosis | 1 |
| Zucherman 2004 | Surgical versus non-surgical treatment for lumbar spinal stenosis | 1 |
| Novartis 2008 | Anti-IgE therapy for allergic bronchopulmonary aspergillosis in people with cystic fibrosis | 1 |
| Konstadoulakis 2006 | Splenectomy for people with thalassaemia major or intermedia | 1 |
| Auckland reduced fat 1999 | Effects of total fat intake on body weight | 1 |
| BDIT Pilot Studies 1996 | Effects of total fat intake on body weight | 1 |
| beFIT 1997 | Effects of total fat intake on body weight | 1 |
| Bloemberg 1991 | Effects of total fat intake on body weight | 1 |
| BRIDGES 2001 | Effects of total fat intake on body weight | 1 |
| Canadian DBCP 1997 | Effects of total fat intake on body weight | 1 |
| de Bont 1981 non-obese 0000 | Effects of total fat intake on body weight | 1 |
| de Bont 1981 obese 0000 | Effects of total fat intake on body weight | 1 |
| DEER 1998 exercise men 0000 | Effects of total fat intake on body weight | 1 |
| DEER 1998 exercise women 0000 | Effects of total fat intake on body weight | 1 |
| DEER 1998 no exercise men 0000 | Effects of total fat intake on body weight | 1 |
| DEER 1998 no exercise wom 0000 | Effects of total fat intake on body weight | 1 |
| Diet and Hormone Study 2003 | Effects of total fat intake on body weight | 1 |
| Kentucky Low Fat 1990 | Effects of total fat intake on body weight | 1 |
| Kuopio Reduced & Mod 1993 | Effects of total fat intake on body weight | 1 |
| Kuopio Reduced Fat 1993 | Effects of total fat intake on body weight | 1 |
| Mastopathy Diet 1988 | Effects of total fat intake on body weight | 1 |
| MeDiet 2006 | Effects of total fat intake on body weight | 1 |
| Moy 2001 | Effects of total fat intake on body weight | 1 |
| MSFAT 1995 | Effects of total fat intake on body weight | 1 |
| NDHS Open 1st L&M 1968 | Effects of total fat intake on body weight | 1 |
| NDHS Open 2nd L&M 1968 | Effects of total fat intake on body weight | 1 |
| Nutrition & Breast Health 0000 | Effects of total fat intake on body weight | 1 |
| Pilkington 1960 | Effects of total fat intake on body weight | 1 |
| Polyp Prevention 1996 | Effects of total fat intake on body weight | 1 |
| Rivellese 1994 | Effects of total fat intake on body weight | 1 |
| Simon Low Fat Breast CA 0000 | Effects of total fat intake on body weight | 1 |
| Sondergaard 2003 | Effects of total fat intake on body weight | 1 |
| Strychar 2009 | Effects of total fat intake on body weight | 1 |
| Swedish Breast CA 1990 | Effects of total fat intake on body weight | 1 |
| Veterans Dermatology 1994 | Effects of total fat intake on body weight | 1 |
| VYRONAS 2009 | Effects of total fat intake on body weight | 1 |
| WHEL 2007 | Effects of total fat intake on body weight | 1 |
| WHI 2006 | Effects of total fat intake on body weight | 1 |
| WHT Feasibility 1990 | Effects of total fat intake on body weight | 1 |
| WHT:FSMP 2003 | Effects of total fat intake on body weight | 1 |
| WINS 1993 | Effects of total fat intake on body weight | 1 |
