# Supplementary material for: Assessing the risk of performance and detection bias in Cochrane reviews as a joint domain is less accurate compared to two separate domains
Source: BMC Med Res Methodol. 2021 Jul 18;21:149. doi: 10.1186/s12874-021-01339-1 (PMC8286598; doi:10.1186/s12874-021-01339-1)
Supplement: Supplementary file 2 — Additional file 2. Table overview of the hypotheses, outcome measures, statistical testsused and results. [file 12874_2021_1339_MOESM2_ESM.docx]

**Supplementary file 2:** Table overview of the hypotheses, outcome measures, statistical tests used and results.

| **Variable/Outcome** | **Null-hypothesis** | **Method of analysis** | **Result** | **Conclusion** |
| --- | --- | --- | --- | --- |
| *Main analysis: Adequacy of judgments for risk of bias associated with performance and detection bias* | Judgments assigned by Cochrane review authors are concordant to our calculation according to the Cochrane Handbook | Wilcoxon test (paired samples) | p<0.05 | Null hypothesis rejected: significant difference between groups. |
|  | The overall proportion of adequate RoB judgments is the same in joint domain (58.7%) as in performance bias domain (73.6%) | Chi-squared test | p<0.0001 | Null hypothesis rejected: significant difference between groups. |
|  | The overall proportion of adequate RoB judgments is the same in joint domain (58.7%) as in detection bias domain (77.9%) | Chi-squared test | p<0.0001 | Null hypothesis rejected: significant difference between groups. |
|  | The overall proportion of adequate RoB judgments is the same in performance bias domain (73.6%) as in detection bias domain (77.9%) | Chi-squared test | p<0.0001 | Null hypothesis rejected: significant difference between groups. |
| *Secondary analysis: Distribution of types of outcomes in observed domains* | Distribution of judged outcomes into three categories of outcomes is the same in joint domain for performance and detection bias as in domain for performance bias | Mann-Whitney | p<0.05 | Null hypothesis rejected: significant difference between groups. |
|  | Distribution of judged outcomes into three categories of outcomes is the same in joint domain for performance and detection bias as in domain for detection bias | Mann-Whitney | p=0.3582 | Null hypothesis accepted: no significant difference between groups. |
| *Secondary analysis: Distribution of reassessed judgments in observed domains* | Distribution of judgments reassessed by our team is the same in joint domain for performance and detection bias as in domain for performance bias | Mann-Whitney | p<0.05 | Null hypothesis rejected: significant difference between groups. |
|  | Distribution of judgments reassessed by our team is the same in joint domain for performance and detection bias as in domain for detection bias | Mann-Whitney | p<0.05 | Null hypothesis rejected: significant difference between groups. |
|  | Distribution of judgments reassessed by our team is the same in domain for performance bias as in domain for detection bias | Mann-Whitney | p<0.05 | Null hypothesis rejected: significant difference between groups. |
| *Secondary analysis: Comparison of adequacy of judgments between domains by categories of outcomes* | Proportion of concordant judgments is the same in the joint domain for performance and detection bias as in the performance bias domain for the subgroup of all or not specified outcomes: 58.1% vs 73.0% | Chi-squared test | p<0.05 | Null hypothesis rejected: significant difference between groups. Alternative hypothesis accepted: subgroup of all or not specified outcomes has higher proportion of adequate judgments in joint domain for performance and detection bias than in the performance bias domain. |
|  | Proportion of concordant judgments is the same in the joint domain for performance and detection bias as in the detection bias domain for the subgroup of all or not specified outcomes: 58.1% vs 76.9% | Chi-squared test | p<0.05 | Null hypothesis rejected: significant difference between groups. Alternative hypothesis accepted: subgroup of all or not specified outcomes has higher proportion of adequate judgments in joint domain for performance and detection bias than in the detection bias domain. |
|  | Proportion of concordant judgments is the same in the joint domain for performance and detection bias as in the performance bias domain for the subgroup of objective outcomes: 81.4% vs 85.9% | Chi-squared test | p=0.4669 | Null hypothesis accepted: no significant difference between groups.  Also, sample too small (β>0.2). |
|  | Proportion of concordant judgments is the same in the joint domain for performance and detection bias as in the detection bias domain for the subgroup of objective outcomes: 81.4% vs 71.6% | Chi-squared test | p=0.0662 | Null hypothesis accepted: no significant difference between groups.  Also, sample too small (β>0.2). |
|  | Proportion of concordant judgments is the same in the joint domain for performance and detection bias as in the performance bias domain for the subgroup of subjective outcomes: 57.3% vs 84.7% | Chi-squared test | p<0.05 | Null hypothesis rejected: significant difference between groups. Alternative hypothesis accepted: subgroup of subjective outcomes has lower proportion of adequate judgments in joint domain for performance and detection bias than in the performance bias domain. |
|  | Proportion of concordant judgments is the same in the joint domain for performance and detection bias as in the detection bias domain for the subgroup of subjective outcomes: 57.3% vs 86.9% | Chi-squared test | p<0.05 | Null hypothesis rejected: significant difference between groups. Alternative hypothesis accepted: subgroup of subjective outcomes has lower proportion of adequate judgments in joint domain for performance and detection bias than in the detection bias domain. |
|  | There are no outside values of far out values for numbers of inadequate judgments throughout subgroups of subjective outcomes and risk of bias judgments: Clinician RRR: High risk – N = 22, Low risk – N = 56, Unclear risk – N = 33; Patient RRR: High risk – N = 17, Low risk – N = 4, Unclear risk 25; Subjective: High risk – N = 0, Low risk – N = 11, Unclear risk – N = 19. | Outlier detection | Outside value detected | According to Tukey, 1977: For Clinician RRR outcomes with judgment of low risk there is an outside value of 56 inadequate judgments. |
| *Secondary analysis: Distribution of outcomes and adequacy of judgments when joint domain for detection and performance bias is split according to various outcomes* | Distribution of categories of outcomes in the whole sample of the joint domain for performance and detection bias is the same as in an overall sub-sample of trials when joint domain is split according to various outcomes | Mann-Whitney | p<0.05 | Null hypothesis rejected: significant difference between groups. |
|  | Proportion of concordant judgments in the whole sample of the joint domain for performance and detection bias is the same as in an overall sub-sample of trials when joint domain is split according to various outcomes: 59% vs 49% | Chi-squared test | p<0.05 | Null hypothesis rejected: significant difference between groups. Alternative hypothesis accepted: proportion of concordant judgments in the whole sample of the joint domain for performance and detection bias is significantly higher than in an overall sub-sample of trials when joint domain is split according to various outcomes. |
|  | Proportion of concordant judgments in the whole sample of the joint domain for performance and detection bias is the same as in an overall sub-sample of trials when joint domain is split according to various outcomes: 59% vs 58% | Chi-squared test | p=0.9778 | Null hypothesis accepted: no significant difference between groups.  Also, sample too small (β>0.2). |
|  | Proportion of concordant judgments in the whole sample of the joint domain for performance and detection bias is the same as in an overall sub-sample of trials when joint domain is split according to various outcomes: 59% vs 44% | Chi-squared test | p<0.05 | Null hypothesis rejected: significant difference between groups. Alternative hypothesis accepted: Proportion of concordant judgments in the whole sample of the joint domain for performance and detection bias is significantly higher than in an overall sub-sample of trials when joint domain is split according to various outcomes. |
